# Brain activity, disruption and connectivity comparisons identify origins of human metacognition in other primates

**DOI:** 10.1038/s41562-026-02473-w

**Published:** 2026-05-18

**Authors:** Kentaro Miyamoto, Simone D’Ambrogio, Caroline Harbison, Nicole Eichert, Urs Schüffelgen, Andrew Emberton, Jerome Sallet, Rogier B. Mars, Nima Khalighinejad, Matthew FS Rushworth

**Affiliations:** 1 Laboratory for Imagination and Executive Functions, RIKEN Center for Brain Science, Wako, Japan; 2 Department of Experimental Psychology, University of Oxford, Oxford, UK; 3 Wellcome Centre for Integrative Neuroimaging, Nuffield Department of Clinical Neurosciences, John Radcliffe Hospital, University of Oxford, Oxford, UK; 4 Donders Institute for Brain, Cognition and Behaviour, Radboud University Nijmegen, Nijmegen, the Netherlands

**Keywords:** Decision, Consciousness

## Abstract

Planning requires anticipating the environmental contingencies that we will encounter and also our own future behaviour in those scenarios. The evolutionary origins of such prospective decision simulations have, however, been difficult to investigate. Moreover, in humans, these metacognitive processes are associated with a distinctively human brain region in the anterior lateral prefrontal cortex. Here we demonstrate these capacities in macaques and their neural bases in two complementary patterns of brain activity in different ventrolateral prefrontal areas: areas 45a and 47/12o. We also examine the impact of ultrasonic disruption of each area. We compare behavioural, brain activity and disruption patterns in humans and macaques. Finally, comparative connectional analysis revealed similarities between the conjunction of the two circuits associated with areas 45a and 47/12o in macaques and the human anterior lateral prefrontal area. In combination, the results suggest behavioural and anatomical origins of metacognitive processes that have become especially sophisticated in humans.

## Abstract (web summary)

Miyamoto et al. show that human metacognition depends on multiple signals in the anterior lateral prefrontal cortex, whereas in macaques it relies on distributed processing in multiple prefrontal areas.

## Main

Planning for the future under uncertainty requires evaluating and comparing the different contingencies we might encounter, as well as assessing how we ourselves might perform in those circumstances. One of the reasons why humans might be such good planners and decision-makers is that they possess a capacity for prospective metacognition and are able to envisage how they will perform in the future^1,2^. Such abilities make it possible to ascertain when mistakes are likely to be made even before they are actually made^3^.

While there has been considerable interest in the nature of human metacognition, it has been difficult to study prospective suppositional thinking in other animals. As a result, our understanding of its broader biological origins is limited. In humans, metacognition and prospective simulation of future decision-making has been linked to the frontal pole and anterior lateral frontal cortex^4,5^. These are brain areas that, more than any other prefrontal areas, are substantially different in humans^6,7^. However, even if these abilities are especially sophisticated in humans, they may still have a link to the cognitive processes and brain mechanisms possessed by other primates^8^.

In the current series of studies, first, we demonstrate a task that makes assessment of prospective metacognition possible in macaques. We show that prospective stimulation of future decision-making is weaker in macaques but present. We then present evidence from recording and causal intervention studies for the existence, in the macaque brain, of two specialized zones, 47/12o^9^ at the border between the ventrolateral prefrontal cortex and the orbitofrontal cortex and area 45a^10,11^ in the ventrolateral prefrontal cortex that are, respectively, concerned with evaluation of future contingencies linked to potential states of the environment and future behaviours that might be performed in those states. We also examine the relationships between these areas of the macaque prefrontal cortex and areas in the human prefrontal cortex linked to related abilities.

**Fig. 1:**
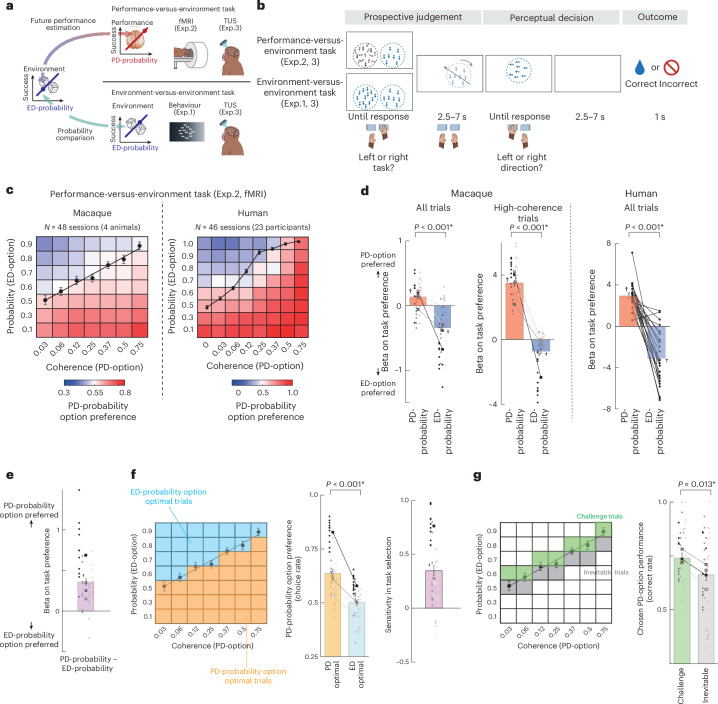
Prospective probability judgement task and behaviour. **a**, During prospective decision-making, animals compared two different contingencies. When both options depended on an environmental contingency, a simple probability comparison was all that was needed (ED-probabilities in the environment-versus-environment task). However, if one option is linked to a performance-dependent contingency, then prospective estimation of PD-probability and its comparison with an ED-probability are required before the decision is attempted (performance-versus-environment task). We first checked that macaque monkeys have the ability to prospectively compare ED-probabilities in the future in the environment-versus-environment task (Exp.1) and then recorded both behaviour and brain activity using fMRI during prospective judgements about future PD- and ED-probabilities (Exp.2). In Exp.3, we applied TUS to the areas that had been found in Exp.2 to carry activity related to task-relevant decision variables and examined the causal impact of perturbing the areas on prospective judgements of both PD- and ED-probabilities (Exp.3). **b**, For prospective judgements, the PD-probability, the probability that one will correctly perform a task (made more or less difficult by modulation of the motion coherence of RDK), and the ED-probability, the probability that a correctly performed task will lead to a reward (defined by the number of dots contained in the RDK), must be considered. Four macaques learned to compare two options that varied in either PD- or ED-probability. Animals made prospective judgements at the first stage of each trial to pursue either a task defined by its PD-probability (left white RDK in the example) or ED-probability (right blue RDK in the example) at the following perceptual decision stage. They did this by moving their hand inside the left or right sensor box. If they correctly classified the motion direction (left or right) of the chosen task (RDK) at the perceptual decision stage, then the animal had a chance of receiving a juice reward. The probability of a reward was indicated by the number of dots in the RDK stimulus. In the other task (environment-versus-environment task, bottom), animals were offered two environment-dependent contingency options with different levels of probability. **c**, Animals’ (left) and humans’ (right) choices of the PD-option in the performance-versus-ED-probability task at the prospective judgement stage at the beginning of the trials increased when either the PD-option’s probability increased, or the environment-dependent contingency option’s probability decreased. The overlaid black line indicates the mean performance at each PD-probability level. Below the black line, animals are more likely to opt for the PD-option, but above it they are more likely to opt for the ED-option. The predominantly red area below the black line indicates that the PD-option was indeed chosen more often in such circumstances. **d**, Beta coefficients of the PD- and ED-probabilities for all trials (left for monkeys and right for humans) and for trials with higher PD-probability (coherence level: 0.25, 0.37, 0.5, 0.75) (middle for monkeys) in logistic multiple regression analysis to predict choices of the PD-option. Monkeys performed the task in a similar manner to humans when a higher PD-probability was offered. **e**, Beta coefficients of the difference of PD- and ED-probabilities (right) in logistic multiple regression analysis to predict choices of the PD-option. **f**, Proportion of trials in which the PD option was chosen for PD-option optimal trials and ED-option optimal trials (middle), classified by the average performance on the PD-option task (left). Right: sensitivity indices summarizing whether, during the first judgement, monkeys chose the optimal option for the second task stage were positive for all animals. **g**, Left: the PD-probability option task when that option had been paired with high-probability (challenge trials) and low-probability (inevitable trials) ED-options earlier in the first stage of the trial. Right: perceptual performance of chosen PD-probability option task was better in challenge trials than inevitable trials consistently across animals. This suggests monkeys assess their own likely performance levels in the first stage of each trial to predict PD-probability that is specific to the second stage of each trial. *N* = 48 sessions (4 animals) for macaques and *N* = 46 sessions (23 participants) for humans. Each grey dot represents single-session data. Data from the same subjects are connected by a line. Two-way ANOVA (PD/ED options and animals) are used for statistical tests. †*P* < 0.001, *t*-test against zero. Error bar: s.e.m. across sessions or participants.

Our approach is based on the fact that some reward contingencies are due to environmental constraints^12^. Such reward contingencies are independent of, or external to, decision-makers themselves. For example, the chances of finding a desirable fruit might sometimes simply depend on which type of tree a monkey is in regardless of how hard the monkey searches. We refer to the probabilities that depend on these contingencies as environment-dependent probabilities (ED-probabilities). Other future reward contingencies, however, depend on how well a given task will be performed in the future by the decision-maker. For example, the chances of finding a different fruit in other trees might, instead, depend on how high or quickly a monkey might climb or how effectively it is able to identify indicators of the fruit’s presence. Such contingencies are dependent on the decision-maker’s own performance levels, and so we refer to them as performance-dependent probabilities (PD-probabilities; Fig. 1a). If a monkey plans for the future, then it should evaluate both types of probabilities but whether and how it does so is unclear. Here, first, we investigated whether macaques estimate future ED-probabilities (Fig. 1b, bottom) or PD-probabilities (Fig. 1b, top) to decide which future states or tasks they should pursue.

Having established that macaques can estimate both types of future probability, we then examine the neural mechanisms on which these abilities depend. By recording across the brain, we identified activity related to ED- and PD-probabilities in areas 47/12o and 45a respectively. We then examined the impact of disrupting activity in each of these areas (Fig. 1b). Finally, we looked at the relationship between these two macaque prefrontal areas and the human anterior lateral prefrontal cortex (alPFC), which has been linked to similar cognitive processes^5,13,14^. We examined the patterns of functional activity in human alPFC and in the macaque areas that we link to estimation of future ED- and PD-probabilities, areas 47/12o and 45a, and identified an intriguing pattern of potential correspondence between species that suggests a common origin.

## Results

### Macaques have a prospective performance estimation ability that allows them to estimate and compare future performance- and environment-dependent contingencies

Four macaques performed a version of tasks that have been described as assessing people’s metacognitive estimations of their own future performance levels as well as future ED-probabilities^5,13,14^. The task required the macaques to estimate future ED-probabilities and future PD-probabilities. We start by explaining the final stage of each trial in the task that used random-dot kinematogram (RDK) stimuli (Fig. 1b). In the final stage of each trial, animals touched either a left or right sensor box depending on the direction in which most dots moved (the ‘coherent’ dot direction). The number of dots indicated the ‘environmental probability’ of receiving a reward when the correct response was made; if there were more (fewer) dots, then the probability of reward was higher (lower). This ED-probability was independent of the animal’s own performance levels. The coherence of the synchronized dot motions, however, determined the animal’s performance levels and, therefore, it determined the PD-probability of whether a reward would be received; monkeys were more likely to perform correctly when coherence levels were high as opposed to low.

We investigated whether monkeys foresaw the value of future task stimuli they might engage with (task options with high or low environment state-dependent probabilities) and if they had insight into how well they themselves were likely to perform tasks varying in coherence before engaging in the tasks (PD-probabilities). In the first stage of each trial (Fig. 1b), the macaques could choose between two options. One of the options always had a very high PD-probability, but its ED-probability varied from trial to trial; its high coherence meant that macaques could almost certainly perform the decision correctly, but its dot number varied from trial to trial so that the option’s ED-probability varied from trial to trial. We therefore refer to this option as the ED-probability option (ED-option). The other option was defined, by contrast, by a very high ED-probability; there was always a high number of dots indicating that a reward would certainly be given to the macaque as long as the decision was performed correctly. However, this second option varied from trial to trial in terms of its PD-probability; trial-to-trial variation in its coherence meant that macaques had to estimate how likely they were to perform the second stage of the task correctly before they chose that option. We therefore refer to this option as the PD-probability option (PD-option). Macaques expressed their preferences about which perceptual decisions to pursue, before actually making the perceptual decision, during an initial first judgement stage that occurred at the beginning of every trial. At that point in the trial, animals had the opportunity to choose one of two decision problems they wanted to attempt in the second stage of the trial.

In summary, each trial comprised an initial judgement phase in which future PD- and ED-probabilities were assessed. This was then followed by the perceptual decision the monkey chose to pursue (Fig. 1b). In the first judgement stage, animals chose one of two simultaneously presented RDK stimuli. Then, at the second stage, they performed a perceptual decision task with the RDK stimulus they had selected (after rotation by either +90° or −90°; the rotation ensured that animals were making an assessment of the decision difficulty in the first judgement period but could not actually make the specific left–right decision that would be required in the second stage of the decision). The animals were rewarded only after the second stage. The first judgement phase is, therefore, an opportunity for animals to select one of two decision tasks to perform in the second stage, and the animal’s aim is to select the decision task through which they are most likely to obtain a reward. A stimulus, which could appear on either the left or right of the screen, presented in white, represented the PD-option (coherence varied between 3% and 75%). A reward outcome would always ensue after correct performance of the PD-probability task (that is, the environment-dependent reward probability was 1). The other stimulus represented the ED-probability task and contained a smaller number of blue dots (ranging from 10 to 90, indicating environmental reward probabilities between 0.1 and 0.9). However, all dots moved in the same direction (100% coherence, ensuring a high PD-probability of success; that is, the PD-probability was close to 1). Thus, in choosing this option, animals had to focus on evaluating the likelihood of success associated with transitioning to this environmental state, with little need to consider their future performance ability because the task was highly easy.

**Extended Data Fig. 1:**
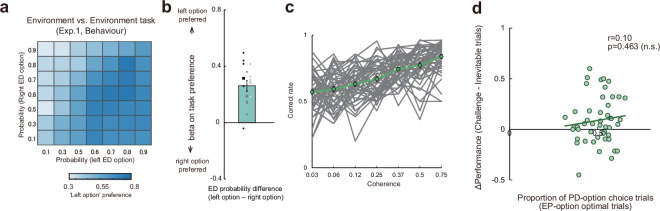
Across-animal and inter-species consistency of prospective judgements about future PD-probabilities and ED-probabilities. (**a**) Animals’ choice of the left option in the environment-vs-ED-probability task at the first, prospective judgment stage of the trials increased when either the left option’s ED-probability increased or the right option’s ED-probability decreased. (**b**) In the environment-vs-environment task the influence of the difference in ED-probabilities between the left and right options on choices of the left option can be summarized by the beta coefficients of the difference in ED-probabilities between the left and right options in a logistic regression predicting left as opposed to right option choice. (**c**) The rate of correct responses made at each coherence level when the PD-probability option was chosen session by session. Correct rate systematically increases as a function of motion coherence. (**d**) Proportion of PD-option choice in ED-option optimal trials in each session does not predict the improvements in perceptual performance seen in challenge trials compared to inevitable trials in the same sessions (see Fig. 1g). This suggests that animals’ general willingness to exert effort in the decision stage could not explain the improvements seen when animals picked the PD option on challenge trials. N = 15 sessions (3 animals). Each grey dot represents single session data. Data from the same sessions are connected by a line. Error bar: s.e.m across sessions.

First, we examined if the animals (*N* = 15 sessions from three animals tested outside the MRI scanner) could compare two options with different levels of future ED-probabilities and choose the better one. In the task, animals were given two ED-probability options that contained different numbers of blue dots moving downwards. After they chose the left or right ED-probability task option, the chosen option moved to the centre of the screen, and all the dots included in the option moved either leftwards or rightwards. If they judged the dot direction correctly (the task was easy, and they rarely failed this perceptual decision), animals were given a reward in accordance with the probability indicated by the number of dots in the chosen ED-option (Extended Data Fig. 1a). The difference in ED-probability between left and right options (left – right option) had a significantly positive effect on the choice of the left task option as opposed to the right (monkey 1, *t*_4_ = 3.23, *P* = 0.031, *r* = 0.85, 95% confidence interval (CI) 0.07 to 0.98; monkey 2, *t*_4_ = 3.69, *P* = 0.020, *r* = 0.88, 95% CI 0.18 to 0.98; monkey 3, *t*_4_ = 6.38, *P* = 0.0031, *r* = 0.95, 95% CI 0.57 to 0.99; Extended Data Fig. 1b), suggesting that macaques learned the linear relationship between the number of dots and ED-probability.

We next investigated whether the animals (*N* = 48 sessions from four animals) could predict the chance of success for a PD-option and compare the estimate with the environment-dependent reward probability associated with the other option (Fig. 1c, left). We also examined humans (*N* = 46 sessions from 23 participants; Fig. 1c, right). Animals’ choices of the PD-option increased when the PD-probability increased or the ED-probability of the other option decreased (red area in the lower right part of Fig. 1c, left) similarly to humans’ choice (Fig. 1c, right). The overlaid black line indicates the mean actual performance level, in the second trial stage, associated with each coherence level for the PD-option (see also Extended Data Fig. 1c). Below the black line, animals in the first judgement stage of the trial should opt for the PD-option, whereas above it they should opt for the environmental probability option. The red area in the lower right of Fig. 1c shows that this is indeed what they did.

We examined animals’ preferences for the PD-probability as a function of PD-probability and the ED-probability when each of these influences was considered separately (Fig. 1d, left; PD-probability: *z* = 3.63, *P* = 2.82 × 10^−4^, *r* = 0.524, 95% CI 0.285 to 0.701; ED-probability: *z* = −5.22, *P* = 1.78 × 10^−7^, *r* = −0.753, 95% CI −0.857 to −0.595). In general, higher PD-probability levels and higher ED-probability levels, respectively, led to the monkeys being more likely and less likely to opt for the PD-option (red and blue bars in Fig. 1d are positive and negative, respectively; the main observation was maintained when the interaction between PD- and ED-probabilities was included in the multiple regression analysis model as a regressor; see Methods for details). However, careful inspection of Fig. 1d reveals that the average effect of the PD-option was not positive in one of the four animals (second column of data in the red bar in Fig. 1d). This negative coefficient indicates limitations in the capacity of one animal to estimate its own future performance levels when it made a selection in the first judgement phase of each trial.

However, if estimation of future performance levels is limited by the difficulty of perceptual performance in the second stage of the task, then it is possible that animals do not always estimate PD-probabilities for all levels of coherence accurately but that they may do so on trials when the second stage of the task is easier. We therefore conducted the same analysis again but focused on the trials with easier stimuli (higher PD-probability (coherence level: 0.25, 0.37, 0.5, 0.75); Fig. 1d, middle). Now the positive modulation of PD-probability on selection of the PD-option choice was uniformly positive across all four animals (and the negative modulation due to the ED-probability remained; PD-probability: *z* = 6.03, *P* = 1.63 × 10^−9^, *r* = 0.870, 95% CI 0.778, to 0.925; ED-probability: *z* = −4.10, *P* = 4.08 × 10^−5^, *r* = −0.592, 95% CI −0.742 to −0.383). It is noteworthy that the clear coexistence of positive modulation of PD-probability and negative modulation of ED-probability for higher PD-probability trials in monkeys was consistent with that for all trials in humans (PD-probability: *z* = 11.03, *P* = 1.96 × 10^−10^; ED-probability: *z* = −5.49, *P* = 1.59 × 10^−5^) (Fig. 1d, right). Monkeys have prospective metacognitive ability comparable to humans, but it is limited to trials with easy PD-options, where the probability of success can be accurately estimated.

To further examine judgements in the first stages of trials, we carried out logistic multiple regression analyses (Fig. 1e) that revealed that preferences for the PD-option increased in proportion to the difference between the PD-probability and ED-probability (summarized as a single index) in all four animals (monkey 1, *t*_11 _= 5.78, *P* = 1.2 × 10⁻^4^, *r* = 0.866, 95% CI 0.582 to 0.964; monkey 2, *t*_11 _= 3.94, *P* = 0.023, *r* = 0.765, 95% CI 0.313 to 0.930; monkey 3, *t*_11 _= 2.36, *P* = 0.037, *r* = 0.580, 95% CI 0.027, to 0.862; monkey 4, *t*_11 _= 7.53, *P* = 1.1 × 10⁻^5^, *r* = 0.915, 95% CI 0.717 to 0.978; see Methods for details on the statistical model; or alternatively Wilcoxon test across all animals: *z* = 5.61, *P* = 2.01 × 10⁻^8^, *r* = 0.810, 95% CI 0.668, to 0.898). Moreover, all four animals chose the PD-option more often when that option was optimal than when the ED-option was optimal (Fig. 1f left and middle; two-way analysis of variance (ANOVA): main effect of PD- versus ED-option optimal trials, *F*_1,44_ = 129.81, *P* = 1.0 × 10⁻^14^, *ηp*^2 ^= 0.747, 95% CI 0.604 to 0.837; monkey 1, *t*_11 _= 19.7, *P* = 2.5×10⁻^9^, *r* = 0.986, 95% CI 0.952 to 0.996; monkey 2, *t*_11 _= 3.88, *P* = 0.010, *r* = 0.760, 95% CI 0.302 to 0.928; monkey 3, *t*_11 _= 3.54, *P* = 0.018, *r* = 0.730, 95% CI 0.241 to 0.916; monkey 4, *t*_11 _= 3.60, *P* = 0.016, *r* = 0.736, 95% CI 0.252 to 0.918).

We also calculated sensitivity indices to summarize whether, when making the first judgement, the monkeys chose the optimal option for the second task stage. This receiver operating characteristic-based index indicates how optimally animals selected the PD-option (it indexes how often animals select the PD-option when that is indeed the optimal choice to take (‘hit’) and how often they select the PD-option when the ED-option would have been the optimal one to take (‘false alarm’)). The sensitivity indices were significantly positive compared with chance level in all four animals (monkey 1, *t*_11 _= 16.7, *P* = 3.5 × 10⁻^9^, *r* = 0.981, 95% CI 0.939 to 0.995; monkey 2, *t*_11 _= 3.87, *P* = 0.026, *r* = 0.759, 95% CI 0.300 to 0.928; monkey 3, *t*_11 _= 2.19, *P* = 0.050, *r* = 0.551, 95% CI −0.014 to 0.851; monkey 4, *t*_11 _= 3.60, *P* = 0.0041, *r* = 0.736, 95% CI 0.252 to 0.918; or alternatively Wilcoxon test across all animals: *z* = 5.41, *P* = 6.11 × 10⁻^8^, *r* = 0.781, 95% CI 0.623 to 0.883, Fig. 1f, right). These observations confirm that macaques possess the ability to prospectively assess both future ED- and PD-probabilities and to compare them in advance of actual decision-making. These abilities were consistent across four animals.

In summary, macaques do appear to be reliably able to make prospective judgements about future PD-probabilities. Nevertheless, they are not as good at doing this as humans. In macaques, the unsigned beta for PD-probability was significantly smaller than the unsigned beta for environmental-dependent probability (*t*_47_ = −3.66, *P* = 0.00063, *r* = 0.471, 95% CI 0.220 to 0.666). By contrast, in humans, the unsigned beta was comparable between PD- and ED-options (*t*_22_ = −0.98, *P* = 0.33, *r* = 0.205, 95% CI −0.220 to 0.567). To formally compare the size of beta coefficients between PD- and ED-options across species, we calculated the ratio of the unsigned beta for the ED-option against the PD-option for each of the 48 sessions performed by macaques and by the 23 human participants. The ratio was significantly larger than 1 (that is, the size of the beta for the ED-option was larger than that for PD-option) in macaques (*t*_47_ = 3.85, *P* = 3.4 × 10⁻^4^, *r* = 0.489, 95% CI 0.241 to 0.680), whereas it was not significantly different from 1 in humans (*t*_22_ = 1.31, *P* = 0.20, *r* = 0.270, 95% CI −0.156 to 0.606). Moreover, the ratio was significantly different between macaques and humans (*t*_69_ = 2.32, *P* = 0.023, *r* = 0.269, 95% CI 0.036 to 0.475). These observations statistically support our argument that the strength of influence of PD-probability is relatively smaller on macaque decisions compared with human decisions.

Finally, we carried out an analysis to determine whether animals genuinely assessed the likelihood of success they would achieve on the PD-probability option on a trial-by-trial basis, rather than simply recognizing different categories of coherence level in the RDK stimuli. To do this, we focused on the second stage of trials and compared animals’ accuracies on the PD-probability option task when that option had been paired with high-probability and low-probability ED-options earlier in the first stage of the trial (Fig. 1g). If the animals are genuinely assessing what their own performance might be capable of achieving from trial to trial, then a particular pattern of decision-making should emerge that has been reported previously in humans and that has been taken as indicative of trial-by-trial assessment of one’s own likely performance^5^. The logic of the analysis is illustrated on the left in Fig. 1g. In each column, there is one green square and one grey square. Any trials that fall into a pair of green and grey squares are equally difficult in terms of the objectively determined coherence level, which in turn should influence the PD-probability assessment (plotted on the abscissa). Each vertically oriented green and grey pair, however, differs in terms of the ED-option that was also presented in the first stage of the trial. The ED-probability of reward is higher in the case of trials from a green square than for the case of trials from each corresponding grey square below. Therefore, animals should choose the PD-option on trials from the green squares only if they are very subjectively certain, as a result of assessment of their own likely future performance levels, that they can perform the decision-making task well. In other words, animals are turning down an option with a high ED-probability of reward if they choose the PD-option on trials illustrated in green, and so they should do this only if they are very confident in their own performance on the other option. We therefore refer to these green trials as ‘challenge’ trials because only the monkey’s subjective confidence about their own performance could have led them to take the challenge of the PD-option when there is another option with a high ED-probability of reward. If this confidence is well placed—if it is based on an accurate assessment of future PD-probability of reward—then animals should do better on the challenge trials they choose to perform in the green cases than they do in corresponding ‘inevitable’ trials shown in grey. This is indeed the pattern of performance that humans produce^5^, and here we now show that it is also the pattern of behaviour that macaques produce (Fig. 1g, right). In brief, macaques produce a surprising pattern of behaviour explainable only in terms of trial-by-trial assessment of their own likely performance levels. Moreover, the pattern is apparent in the data from all four animals (two-way ANOVA: main effect of challenge versus inevitable trials, *F*_1,44_ = 6.69, *P* = 0.013, *ηp*^2^ = 0.132, 95% CI 0.018 to 0.313). This behavioural result suggests that monkeys assess their own likely performance levels in the first stage of each trial to predict PD-probability that is specific to the second stage of each trial. Notably, the proportion of PD-option choices in ED-option optimal trials on a session basis, which reflects the general willingness within a session to intentionally choose the economically suboptimal PD-option, did not predict session-based improvement in PD-option task performance in challenge trials compared with inevitable trials (*r* = 0.10, *P* = 0.463, 95% CI −0.188 to 0.368; Extended Data Fig. 1d). Thus, it is unlikely that animals’ assessments of their general willingness to exert effort in the decision stage fully explain the better performance of the PD-option task in challenge trials. A key component of what drives the PD-option choices must be related to trial-specific prospection regarding subtle variation in their likely ability to perform a PD-option at a specific time.

**Extended Data Fig. 2:**
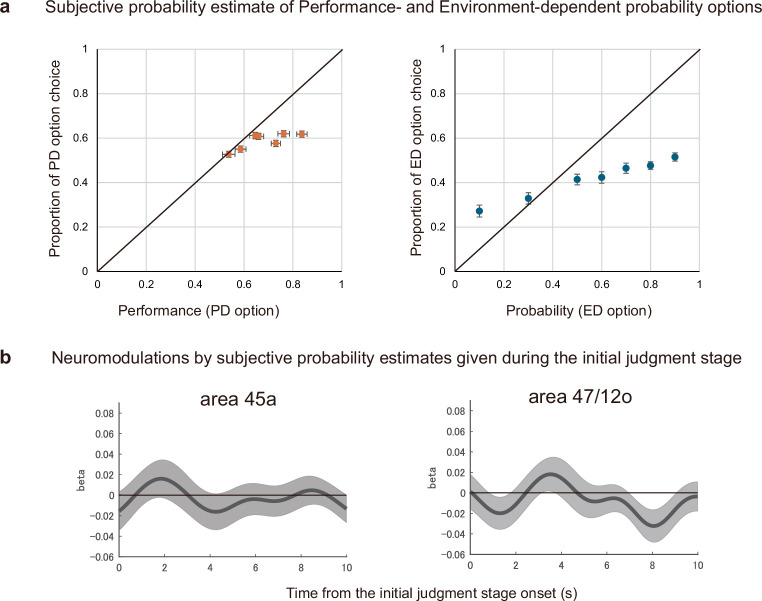
Independent contributions of performance- and ED-options to prospective judgements. (**a**) The proportion of the trials where the animals chose PD- (left panel) and ED- (right panel) options at the initial prospective judgement stage increased systematically with the PD- and environmental probability, respectively. Note, however, that these graphs do not indicate accuracy of decision making. Even when a probability is high (on the right hand of these figures) the corresponding option may not be chosen if the alternative is even better. Accuracy is, however, illustrated instead in Fig. 1c. (**b**) Evolution of regression weights across time, indexing the combined effect of subjective probability estimates of PD- and ED-options which is reflected by the proportion of each option choice, are illustrated for area 45a (left panel) and area 47/12o (right panel). N = 36 sessions (3 animals). Error bar and shade: s.e.m across sessions.

We briefly note one further piece of evidence that PD- and ED-probabilities are processed in different ways. We examined the rates at which animals chose options at each ED-probability level to estimate the animals’ subjective preferences for each environment-dependent level of probability. We then carried out the same analysis for the PD-options. The difference between the two subjective preference curves (Extended Data Fig. 2) indicates that the objective probabilities of reward associated with performance-dependent and environment-dependent contingencies are translated into subjective preferences in different ways (three-way ANOVA: interaction of two probability options and seven probability levels, *F*_6,671_ = 28.44, *P* < 1.0 × 10^−16^, *ηp*^2^ = 0.203, 95% CI 0.162 to 0.243).

### Parallel mechanisms for estimating future PD- and ED-probabilities

**Fig. 2:**
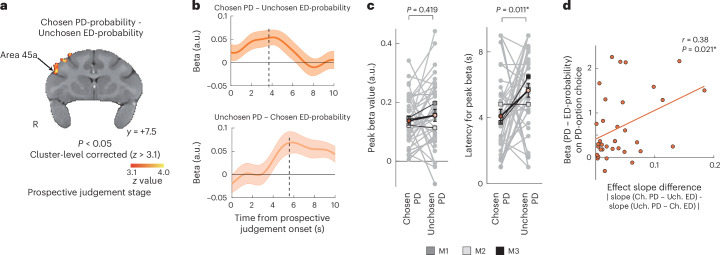
Activity linked to prospective evaluation of PD-probability in area 45a in the first prospective judgement phase of each trial predicts selection of the PD-option for the second perceptual decision-making stage. **a**, Left: activity in area 45a was modulated significantly by the difference between the PD-probability when that option was chosen and the ED-probability associated with the other option during the initial prospective judgement stage of the trial (whole-brain effects FWE cluster corrected with *z* > 3.1 and *P* < 0.05). **b**, The evolution of regression weights across time, indexing the effect of PD-probability versus ED-probability on haemodynamic activity when the PD-option was chosen (top) or rejected (bottom), is illustrated for area 45a. There was a shorter latency before activity peaked in the first case. This is consistent with a faster accumulation of evidence concerning PD-probability for an option that was ultimately chosen than for an option that was ultimately rejected. **c**, The difference in latency for the signal peak in area 45a when the PD-probability option was chosen or unchosen (right) was significant during prospective judgements, but there was no difference in the peak signal height (left). The tick line indicates the mean across sessions. **d**, The difference in the slope of the PD-probability effect in area 45a, between trials where the PD-option was itself ultimately chosen and trials where the other option was ultimately chosen, was correlated with the behavioural influence of the difference between PD- and ED-probabilities (as indexed by the beta coefficient of the difference between PD- and ED-probabilities on PD-option selection; see also Fig. 1e). *N* = 36 sessions (3 animals). Each grey dot and line represents single-session data. The mean values for each animal are connected by a black line. Two-way ANOVA (chosen/unchosen PD options and animals) are used for statistical tests. Error bar and shade: s.e.m. across sessions. Ch., chosen; Uch., unchosen.

**Extended Data Fig. 3:**
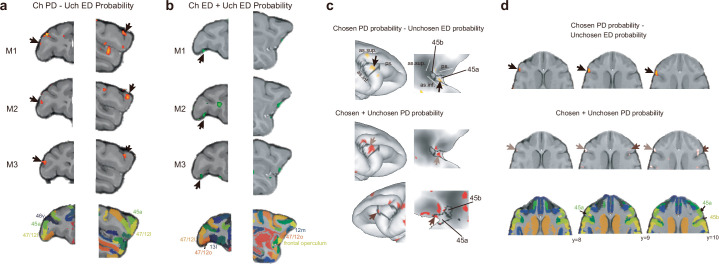
Across-animal consistency of activation peaks in area 45a and 47/12o. (**a**) Activition clusters of individual animals (M1, M2, M3) in response to ‘chosen PD-probability – unchosen ED-probability’ during the prospective judgement (p < 0.05, FWE cluster-level corrected) on the coronal (left column) and sagittal (right column) slices of the right hemisphere (Z > 2.0 for a display purpose) along with the border of anatomical regions (bottom panels). The activation peak was confined to area 45a in all three animals. (**b**) Activition clusters of individual animals (M1, M2, M3) in response to ‘ED-probability’ irrespective of choice during the prospective judgement (p < 0.05, FWE voxel-level corrected) on the coronal (left column) and sagittal (right column) slices of the right hemisphere (p < 0.025 uncorrected for purpose of display) along with the border of anatomical regions (bottom panels). The activation peak was confined to area 47/12o for all three animals. (**c**) Demonstration of group activation clusters on the 3D CARET surface (left column) and flat map (right column) in response to ‘chosen PD-probability – unchosen ED-probability’ (p < 0.05, FWE cluster-level corrected) (top panels) and ‘PD-probability’ irrespective of choice (p < 0.025 uncorrected for a display purpose) (bottom panels) during the prospective judgement. Activation peak was confined to area 45a rather than area 45b. (**d**) Demonstration of group activation clusters in panel **c** on a series of axial slices. N = 12 sessions for each animal for panels **a**–**b**. N = 36 sessions in total (3 animals) for panels **c**–**d**.

**Extended Data Fig. 4:**
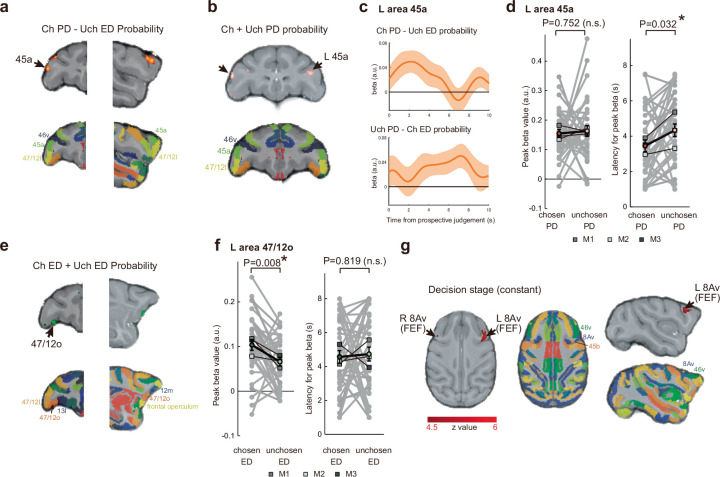
Representation of PD- and ED-probability in bilateral area 45a and 47/12o, respectively during prospective judgements. (**a**) Group activition clusters in response to ‘chosen PD-probability – unchosen ED-probability’ during the prospective judgement (p < 0.05, FWE cluster-level corrected) on the coronal (top left panel) and sagittal (top right panel) slices of the right hemisphere. To clarify the borders of anatomical regions on the same slices, we have registered the ‘Cortical Hierarchy Atlas of the Rhesus Macaque (CHARM)’^68,69^ to our group template in CARET F99 macaque space (bottom panels). The activation peak was confined to area 45a (see also Extended Data Fig. 4c–d). (**b**) Group activition clusters in response to ‘PD-probability’ irrespective of the animal’s choice during the prospective judgement (p < 0.01) (top panel) and the borders of anatomical regions on the same slices (bottom panel). The bilaretal activation peaks were confined into area 45a (see also Extended Data Fig. 4c–d). (**c**) Evolution of regression weights across time, indexing the effect of PD-probability versus ED-probability on hemodynamic activity when the PD-option was chosen (top) or rejected (bottom) for the left area 45a in panel **b**. See also Fig. 2b for the right area 45a. (**d**) The difference in latency for the signal peak in the left area 45a when the PD-probability option was chosen or unchosen (right) was significant during prospective judgments, but there was no difference in the peak signal height (left). The tick line indicates the mean across sessions.

Next, to search for magnetic resonance imaging (MRI)-measured brain activity linked selectively to estimation of future PD- and ED-probabilities (related to Fig. 1d, left) during the first metacognition stage of trials, we examined first-stage judgements on which macaques made choices between the PD- and ED-options (36 sessions from Fig. 1d,f); that is, they chose the PD-probability option as opposed to the ED-option. We sought brain activity covarying significantly with the variable that had guided the first-stage judgement—the chosen PD-probability as opposed to the unchosen ED-probability by recording activity across the macaque brain using functional MRI (fMRI)^15–17^ during task performance (*P* < 0.05, cluster-level family-wise error (FWE)-corrected (*z* > 3.1), Fig. 2a, left). Activity that covaried with changes in the decision variable guiding the first-stage judgement was most prominent in ventrolateral prefrontal area 45a (based on Gerbella et al.^10^, Montreal Neurological Institute coordinates, (*x*, *y*, *z*) = (21.1, 7.5, 12.5), *z* = 3.75). The group activity at area 45a was consistent across three animals when analysed separately (Extended Data Figs. 3 and 4a). We also found an area TAa of activity at (*x*, *y*, *z*) = (−29.2, −7.0, −4.0), but no other activity was detected at this threshold. It was noteworthy that area 45a was active in correlation with PD-probability both when monkeys chose to pursue the PD-option and when they decided not to (Extended Data Fig. 4b). Activity in area 45a increased with the PD-probability in both cases (Fig. 2b) but the increases differed in terms of their timing—the latency until the peak activity was reached. Activity linked to PD-probability increased more quickly when monkeys chose to pursue the PD-probability option as opposed to when they rejected it (latency to peak: two-way ANOVA of the main effects of two PD-probability option choices (chosen, unchosen) and three individual animals (M1, M2, M3); main effect of the PD-option choice, *F*_1,69_ = 6.67, *P* = 0.011, *ηp*^2^ = 0.088, 95% CI 0.017 to 0.199; interaction between the choices and animals, *F*_2,69_ = 1.82, *P* = 0.16, *ηp*^2^ = 0.050, 95% CI 0.000 to 0.138; Fig. 2c). The latency difference was not explained by the difference in maximum peak height; the maximum beta weights at the peak were comparable regardless of whether the PD-option was chosen or unchosen (main effect of PD-probability option choices, *F*_1,69_ = 0.66, *P* = 0.41, *ηp*^2^ = 0.009, 95% CI 0.000 to 0.073; interaction, *F*_2,69_ = 0.57, *P* = 0.56, *ηp*^2^ = 0.016, 95% CI 0.000 to 0.095). These characteristic features of the evolution of regression weights across time were consistent with those in the homotopic region in the left hemisphere (left area 45a) (Extended Data Fig. 4b), whose activity changes in proportion to the PD-probability, irrespective of whether the PD-option is ultimately chosen (Extended Data Fig. 4c). As in the right area 45a, we confirmed that the main effect of the PD-option choice was significant (*F*_1,69_ = 4.74, *P* = 0.032, *ηp*^2^ = 0.064, 95% CI 0.004 to 0.167) in the absence of any interaction with the identity of the individual animal from which the data were recorded (*F*_2,69_ = 0.48, *P* = 0.620, *ηp*^2^ = 0.014, 95% CI 0.000 to 0.089) (Extended Data Fig. 4d, right). Moreover, again as in the right area 45a, there were no significant differences in the height of the peak beta values and this was also consistent across animals (main effect of PD-probability option choices, *F*_1,69_ = 0.10, *P* = 0.752, *ηp*^2^ = 0.001, 95% CI 0.000 to 0.052; interaction, *F*_2,69_ = 0.43, *P* = 0.652, *ηp*^2^ = 0.012, 95% CI 0.000 to 0.085) (Extended Data Figs. 4d, left, and 4e,f).

Not only was the latency of the peak in PD-probability-related activity linked to whether that option was chosen on a trial-by-trial basis, but day-to-day variations in PD-probability-related activity were also predictive of day-to-day variations in behaviour; day-to-day variation in the difference in latencies between trials in which the PD-probability task was chosen and those in which it was rejected predicted day-to-day variation in the influence of PD- and ED-probabilities on task selection (Peason’s *r* = 0.38, *P* = 0.021, 95% CI 0.054 to 0.633; Fig. 2d).

**Fig. 3:**
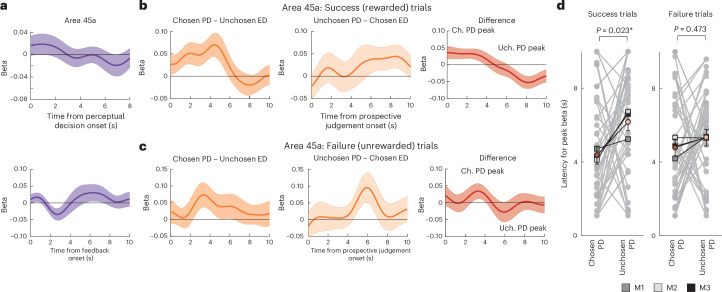
Activity in area 45a during prospective judgement predicts judgements. **a**, Top: the evolution of regression weights across time in area 45a (Fig. 2a), indexing the effect of PD-probability from the onset of perceptual decision-making in the second stage of the trial. Bottom: the evolution of regression weights across time in area 45a, indexing the effect of success (rewarded trial) from the onset of feedback presentation after the perceptual decision. Unlike in the first judgement phase of the trials, no significant task-evoked modulations were observed during the perceptual decision stage itself or after feedback was delivered in the feedback phase of the second, perceptual decision-making stage. **b**, The evolution of regression weights across time in area 45a, indexing the effect of PD-probability versus ED-probability on haemodynamic activity during prospective judgements in the first stage of the trial when the PD-option was chosen (left) or rejected (middle). Illustrations are for the trials in which animals went on to successfully receive reward after the following perceptual decision stage. Examining the difference (right) between the two traces (left − middle) revealed a significant difference at an early time (2.07 s) that was related to choosing the PD-probability option. Later in time (8.17 s), the difference trace (right) contained a late, negative peak. This reflected the fact that activity related to the PD-probability was later in time when that option was unchosen. **c**, The same data as in **b**, collected in area 45a, at the same time—during the prospective judgements in the first stage of each trial—but for trials on which animals failed to receive rewards at the following perceptual decision stage. All conventions are the same as for **b**. Again, we identified an early peak related to when the PD-option was chosen (3.29 s) and a late peak related to the PD-option when it went unchosen (5.92 s). The early positive and late negative deflections were, however, no longer significant, and they occurred later and earlier, respectively (compare the right-hand line plot with that in **b**). **d**, The difference in latency for the signal peak in area 45a when the PD-option was chosen or unchosen (shown in the right-hand line plots of **b** and **c**) was significant for successful trials (left) but not for trials that ended in failure (right). The tick line indicates the mean across sessions. *N* = 36 sessions (3 animals). Each grey dot and line represents single-session data. The mean values for each animal are connected by a black line. Two-way ANOVA (chosen/unchosen PD options and animals) are used for statistical tests. Error bar and shade: s.e.m. across sessions.

Moreover, the difference in activity pattern when choosing and not choosing the PD-option was clearer on those trials when the choice was ultimately successful and the second stage decision led to reward (Fig. 3b) as opposed to unsuccessful trials on which the second stage choice led to no reward (Fig. 3c). As already noted, activity peaked at an early time when the PD-probability option was chosen and at a later time when it was left unchosen. The difference in the latency between chosen and unchosen PD-probability peaks was significant for successful trials (main effect of PD-probability option choices, *F*_1,69_ = 5.41, *P* = 0.023, *ηp*^2^ = 0.073, 95% CI 0.007 to 0.179; interaction between the choices and animals, *F*_2,69_ = 0.70, *P* = 0.50, *ηp*^2^ = 0.020, 95% CI 0.000 to 0.103) but not for unsuccessful trials (main effect of PD-probability option choices, *F*_1,69_ = 0.52, *P* = 0.47, *ηp*^2^ = 0.007, 95% CI 0.000 to 0.069; interaction, *F*_2,69_ = 0.21, *P* = 0.81, *ηp*^2^ = 0.006, 95% CI 0.000 to 0.079; Fig. 3d). These observations suggest that area 45a assesses the PD-probability—the animals’ estimates of how likely they themselves will be to perform a task correctly in the future—and that the rate at which activity encoding this information emerges predicts which choices animals will take in prospective judgements about future PD-probabilities.

**Extended Data Fig. 5:**
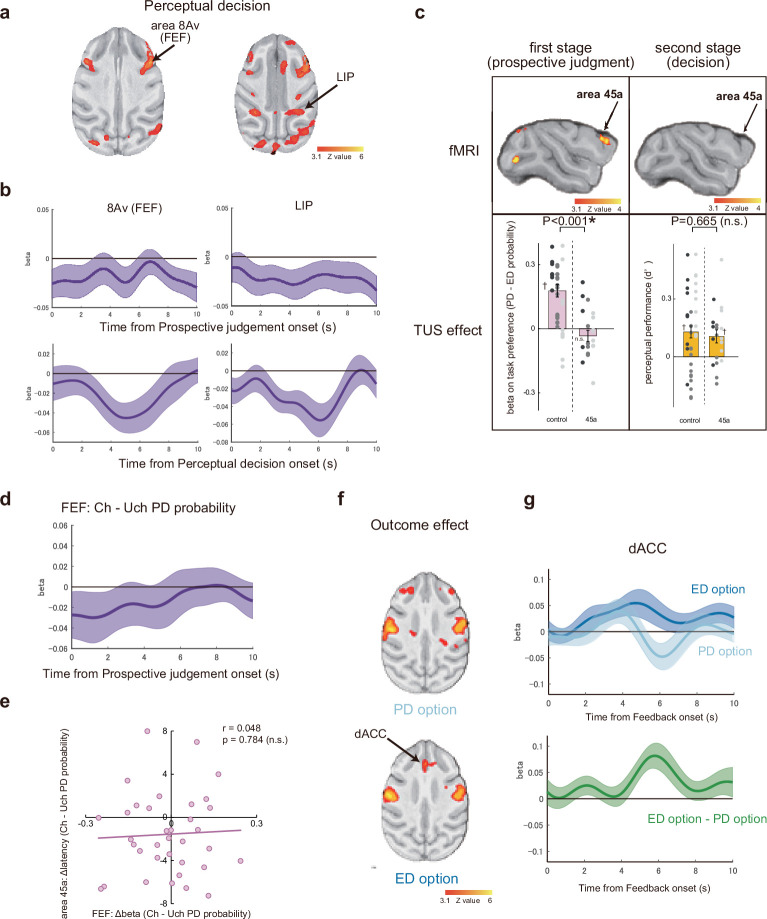
Activity correlated with PD- and ED-probabilities during the second stage of trials when perceptual decisions are made and outcomes (reward / error) occur. (**a**) fMRI activity associated with main effect (constant) of perceptual decision (p < 0.05, FWE cluster-level corrected [z > 3.1]). Bilateral FEF ([x,y,z]=[-19, 8.5, 14.5], [19.5, 10, 13]) and LIP ([x,y,z]=[-14.6, 20.6, 14.6], [14.1, -22.1, 18.6]) were identified. (**b**) Evolution of regression weights across time at left FEF (left) and left LIP (right), indexing the effect of the PD-probability when it was chosen from the onset of the prospective judgement (top) and perceptual decision (bottom) phases at the beginning and end, respectively, of each trial. Significantly negative modulations in relation to PD-probability were salient during the perceptual decision stage but not during the prospective judgement stage. (**c**) The combination of activity in the first stage of each trial, absence of activity in the second stage of each trial, an impact of TUS in the first stage of each trial and an absence of TUS effect in the second stage of each trial suggests a unique role of area 45a for introspective evaluation of performance rather than processes of motion perception involved in FEF. Each dot represents single TUS session data (N = 80 sessions). (**d**) Comparison of FEF modulation during the prospective judgement stage when the PD option was chosen or unchosen. (**e**) Session-by-session difference (N = 36 sessions) in FEF activity (for the effect of PD probability when the option was chosen versus unchosen) could not explain the latency difference in area 45a (r = 0.048, p = 0.78). The result suggests that latency differences in activity modulation in area 45a between occasions when the PD option is chosen versus unchosen cannot be explained by eye movement-related activity in FEF. (**f**) fMRI activity associated with success (rewarded=1, unrewarded = -1) during outcome stage after performance of the PD-task (top) and ED-task (bottom) (p < 0.05, FWE cluster-level corrected [z > 3.1]). Anterior cingulate area (ACC; [x,y,z]=[0, 16,6, 11.1]) was active specifically for success in the ED-task. (**g**) Evolution of regression weights across time in ACC, indexing the effect of success (rewarded) from the onset of feedback presentation. The difference in the size of effect between outcome following the PD-option task (light blue at the top panel) and outcome following the ED-option task (dark blue at the top panel) was significant (bottom panel). N = 36 sessions (3 animals). Each grey dot represents single session data. Error bar and shade: s.e.m across sessions.

It was also noted that, once the PD-probability option was chosen, an anatomically distinct area of cortex, the frontal eye field (FEF) (Extended Data Fig. 4g) and lateral intraparietal area (LIP), encoded the PD-probability during the perceptual decision (Extended Data Fig. 5a,b), rather than area 45a where activity was found during the initial judgement phase of each trial (Fig. 3a). In summary, our findings are broadly consistent with the claim that FEF and LIP activity reflect the accumulation of evidence for a perceptual decision^18,19^, but, in addition, they suggest the existence of another mechanism in a different frontal area, area 45a, that is linked to estimating future performance.

In summary, both FEF, active during the second stage of each trial, and area 45a, which encoded future PD-probability during the first stage of each trial lie anterior to the arcuate sulcus. However, area 45a activity was ventral and anterior to activity related to performance of the perceptual decision-making task in the second stage of each trial. The bilateral peaks of activation clusters during the second stage are located within area 8Av, which corresponds to FEF^20^ (Extended Data Fig. 4g). The clusters of activity were non-overlapping with the activation cluster at area 45a for the prospective judgement (Extended Data Fig. 5c). The activity in FEF was found to be negatively modulated in correlation with PD-probability, with a peak latency of around 5 s consistently between the first prospective judgement and second perceptual decision stages (Extended Data Fig. 5b). This suggests that activity in FEF could reflect the degree of effort to be paid during motion direction discrimination; less effort is needed when there is a high PD-probability option and discernment of the motion direction is easy. We then compared this FEF modulation during the prospective judgement stage when the PD-option was chosen or unchosen. We found that there are no clear differences in FEF activity as a function of whether the PD-option was chosen or unchosen as shown below (Extended Data Fig. 5d). Moreover, session-by-session difference in FEF activity (in the effect of PD-probability when the option was chosen versus unchosen) could not explain the latency difference in area 45a (Pearson’s *r* = 0.048, *P* = 0.784, 95% CI −0.290 to 0.375) (Extended Data Fig. 5e). These results suggest that latency differences in activity modulation in area 45a between occasions when the PD-option is chosen versus unchosen cannot be explained by eye movement-related activity in FEF.

**Fig. 4:**
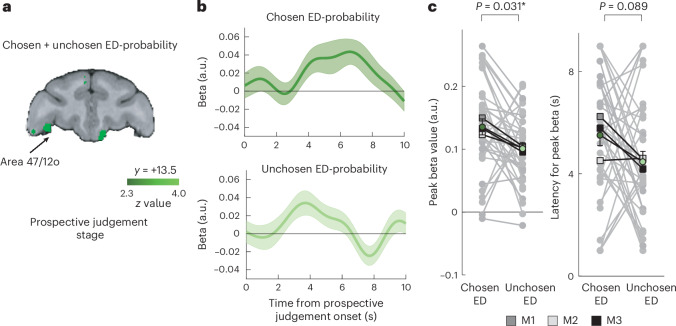
Activity linked to evaluation of ED-probability in area 47/12o. **a**, Activity in 47/12o was modulated significantly (*P* < 0.05, FWE-corrected at voxel level) by the ED-option during the prospective judgement stage in the first part of the trial irrespective of whether animals ultimately chose or not (illustrated at *z* > 2.3 for display purposes). **b**, Although the ED-probability exerted a positive influence on 47/12o activity regardless of whether that option was chosen (top) or rejected (bottom), the modulation was bigger (higher peak) when it was chosen. **c**, Although there was a significant difference in peak signal in area 47/12o when the ED-option was chosen or unchosen (left), there was no difference in latency to the peak signal (right). The tick line indicates the mean across sessions. *N* = 36 sessions (3 animals). Each grey dot and line represents single-session data. The mean values for each animal are connected by a black line. Two-way ANOVA (chosen/unchosen PD options and animals) are used for statistical tests. Error bar and shade: s.e.m. across sessions.

In addition, while still focusing on the first judgement phase of each trial, we searched for brain activity encoding the ED-probabilities associated with other option. Activity was significantly, positively modulated by the ED-probability—the probability of reward cued by the number of dots on the blue stimuli in cortex just lateral to the lateral orbitofrontal sulcus (*P* < 0.05, FWE-corrected, area 47/12o based on Petrides and Pandya^9^; peak activation (*x*, *y*, *z*) = (21.6, 14.5, −1.0), *z* = 5.50; Fig. 4a). No other activity anywhere else in the brain was detected at this threshold in this contrast. The region was close to one previously linked to the encoding of reward probability^21^. The activity in 47/12o was correlated with the ED-probability predicted by a stimulus regardless of whether or not macaques chose that option or the PD-probability option on a given trial (Fig. 4b). Moreover, in contrast to the representation of PD-probability in area 45a, the latency to the maximum peak of the 47/12o modulation was less clearly modulated as a function of whether macaques chose the ED-option or not (main effect of ED-probability option choices, *F*_1,69_ = 3.10, *P* = 0.082, *ηp*^2^ = 0.043, 95% CI 0.000 to 0.132; interaction between the choices and animals, *F*_2,69_ = 0.91, *P* = 0.40, *ηp*^2^ = 0.026, 95% CI 0.000 to 0.115; Fig. 4c and Extended Data Fig. 4e). Instead, however, the degree to which activity was related to ED-probability—the maximum beta relating the fMRI activity to ED-probability at the peak was significantly larger when macaques chose the ED-option as opposed to when it was unchosen (main effect of ED-probability option choices, *F*_1,69_ = 4.85, *P* = 0.031, *ηp*^2^ = 0.066, 95% CI 0.005 to 0.170; interaction, *F*_2,69_ = 0.21, *P* = 0.811, *ηp*^2^ = 0.006, 95% CI 0.000 to 0.079). No clear activation clusters were found in response to ED-probability in left area 47/12o when we conducted analyses that corrected for multiple comparisons across the whole brain. However, when the homotopic left hemisphere region corresponding to right 47/12o (with the same *x* coordinate but with the opposite sign) was examined in a focused manner, just as in the right 47/12o (Fig. 4c), the size of the peak beta values at left 47/12o predicted ED-option choice, and this was true consistently across the three individuals (main effect of ED-probability option choices, *F*_1,69_ = 7.24, *P* = 0.008, *ηp*^2^ = 0.095, 95% CI 0.020 to 0.205; interaction, *F*_2,69_ = 1.30, *P* = 0.27, *ηp*^2^ = 0.036, 95% CI 0.000 to 0.128) (Extended Data Fig. 4f). These observations suggest that evaluation of the ED-option occurs at the same rate irrespective of final choice when animals are choosing between the PD- and ED-options (Fig. 1d), but, instead, the maximum activity level reflected whether or not the final choice would be the environmental contingency option.

In summary, we discovered that areas 45a and 47/12o contribute to carrying PD- and ED-probability information, respectively, during the initial judgement stages of trials when macaques decided which option to pursue. Moreover, the manner in which the information in the activity was translated into a choice of one option or the other differed between areas 45a and 47/12o; selection versus non-selection of the performance-related option was associated with faster as opposed to slower increases in the area 45a activity that encoded PD-probability but the peak activity level did not change. By contrast, the rate at which activity in 47/12o rose did not differ significantly between trials when the ED-option was chosen and trials when it was left unchosen. However, the maximum 47/12o activity level was higher in when the environment-contingent option was chosen as opposed to unchosen.

**Fig. 5:**
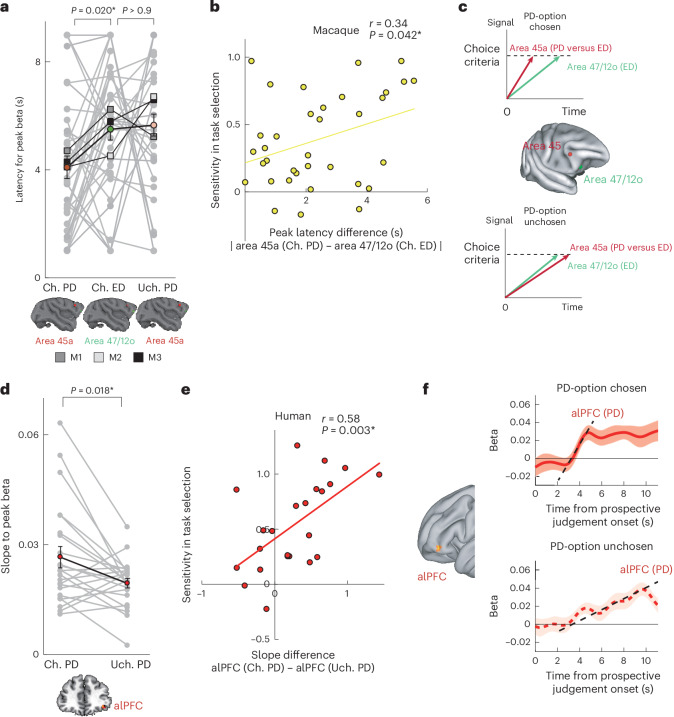
Parallel prospective assessment of PD- and ED-probabilities in areas 45a and 47/12o in macaques. **a**, Comparison of latency to the peak signal for the PD-probability in area 45a when the PD-option was chosen (left), for the ED-probability signal in area 47/12o and for the PD-probability activity in area 45a when it was unchosen in macaques. The PD-probability activity in area 45a peaks before the ED-probability activity in area 47/12o when the PD-option was chosen, but not when the ED-option was chosen. Two-way ANOVA (conditions and animals) demonstrated the main effect of condition (*F*_2,104_ = 4.92, *P* = 0.0091). *P < 0.05 by post hoc multiple comparisons tests with Bonferroni correction (two-sided). **b**, The difference between the latency of the PD-probability-related activity in area 45a on trials when the PD-option was chosen and the latency of the ED-probability-related activity in area 47/12o when the ED-option was chosen is illustrated on the abscissa. Variation in this difference correlated with the accuracy of the first-stage prospective judgements as evaluated by sensitivity in task selection; more separation of the signals from area 47/12o and area 45a leads to more accurate prospective judgements. **c**, A model of prospective judgements in macaques: competition between a decision-making mechanism in area 45a for choosing the PD-option and for the ED-option in area 47/12o. **d**, Comparison of slope to the peak signal for the PD-probability in alPFC when the PD-option was chosen (left) and when it was unchosen (right) in humans. Steepness of slope at alPFC predicts the choice of options alone. A paired *t*-test across participants (two-sided) is applied. **e**, The difference between the slope of the PD-probability-related activity in alPFC on trials when the PD-option was chosen and on trials when it was unchosen correlated with the accuracy of the first-stage prospective judgements as evaluated by sensitivity in task selection. **f**, A model of prospective judgements in humans: a unitary decision-making mechanism in alPFC for choosing the PD-option. *N* = 36 sessions for macaques and *N* = 23 for humans. Each grey dot and line represents single-session data. The mean values for each animal are connected by a black line. Error bars represent s.e.m. across sessions (macaques) or participants (humans).

To examine how the information present in the activity patterns in areas 45a and 47/12o is integrated to choose the more optimal task option on each trial, we compared the latency of the maximum PD- and ED-probability peaks in areas 45a and 47/12o, respectively. The latency of the area 45a PD-probability-related peak was significantly shorter than the latency of the 47/12o ED-probability-related peak when the PD-option was chosen (*t*_34_ = 2.71, *P* = 0.010, *r* = 0.421, 95% CI 0.105 to 0.654; Fig. 5a). By contrast, the latency of the area 45a PD-probability-related peak was not significantly different from the ED-probability-related activity peak in 47/12o when the ED-option was chosen (*t*_34_ = 0.26, *P* = 0.796, *r* = 0.044, 95% CI −0.296 to 0.376). In summary, the time of occurrence of the 47/12o peak encoding ED-probability did not change regardless of which option was finally chosen, but because the activity coding the PD-option rose faster on trials when that option would ultimately be taken, it peaked before the activity encoding ED-probability in 47/12o. Moreover, in addition to these trial-to-trial variations in peak activity latencies, there was also day-to-day variation in peak activity latencies that was related to day-to-day variation in first-stage judgement accuracy; a bigger difference in the latency between area 45a’s PD-probability-related activity and 47/12o’s ED-probability-related activity was associated with higher first-stage judgement efficiency as quantified by meta-*d*′ (Peason’s *r* = 0.34, *P* = 0.042, 95% CI 0.005 to 0.604; Fig. 5b). These findings converge to suggest that competition between decision-making processes in areas 45a and 47/12o underlies the comparison of PD and environmental probabilities to determine which future problem to tackle at the second stage of each trial (Fig. 5c).

It is noteworthy that, in humans, PD-probability was encoded by alPFC (Fig. 5f, left) and the steepness of slope of the alPFC signals (the steepness of the increase in the PD-probability effect; Fig. 5f, right) predicted people’s choices in the prospective judgement stage at the beginning of each trial (Fig. 5d; chosen versus unchosen PD-option, *t*_22_ = 2.54, *P* = 0.018, *r* = 0.476, 95% CI 0.088 to 0.725). Moreover, across participants, variation in the size of this activity pattern was associated with variation in prospective judgement; the bigger the slope difference on trials when the PD-option was chosen and unchosen, the greater the participant’s sensitivity in task selection in the prospective judgement phase (Fig. 5e; Peason’s *r* = 0.58, *P* = 0.0034, 95% CI 0.218 to 0.803). As in macaques, the activity patterns associated with the first metacognitive stage of the trial were not present at the second perceptual decision-making stage. Moreover, in humans, no area encoded only ED-probabilities during the first stage decisions; instead, ED-options also influenced alPFC activity. These observations suggest a unitary decision-making mechanism in alPFC for choosing the PD-option in humans (Fig. 5f), as opposed to separate and competing mechanisms in areas 45a and 47/12o in macaques (Fig. 5c).

Further evidence that animals appreciated the difference between PD- and ED-probabilities was furnished by the presence of distinct activity patterns in the anterior cingulate cortex (ACC) following reward outcomes that were delivered after successful PD- and ED-trials (Extended Data Fig. 5f,g; see also Fig. 3a).

**Extended Data Fig. 6:**
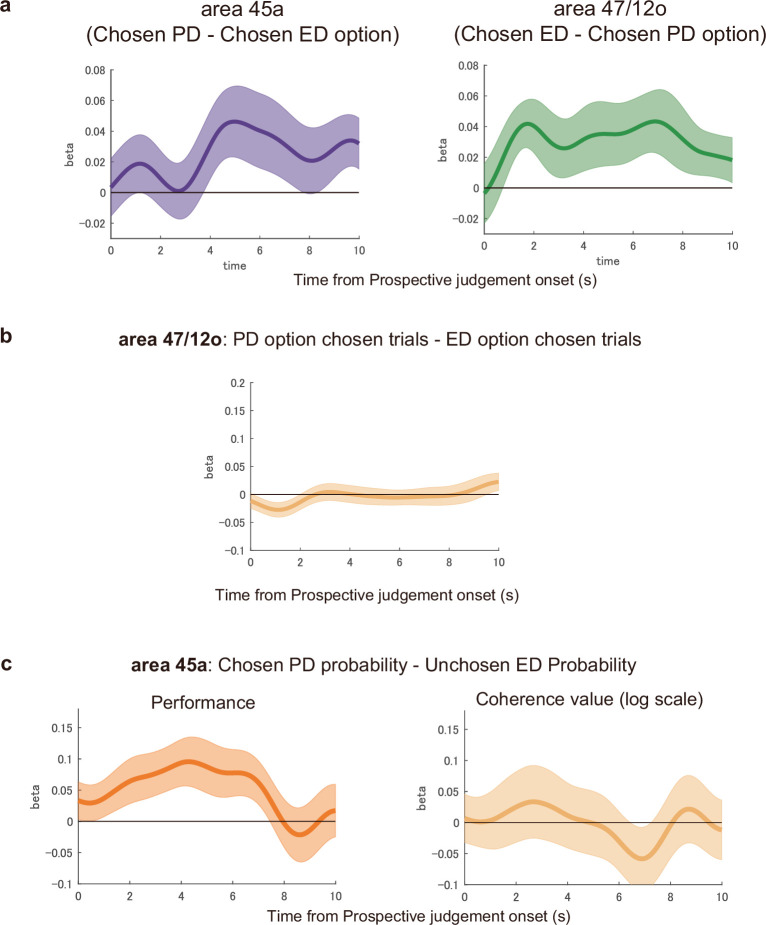
Features of neuromodulation of areas 45a and 47/12o during the prospective judgement stage. (**a**) Evolution of regression weights across time, indexing the difference in PD- and ED-probabilities when each option was chosen, are illustrated for area 45a (left panel) and area 47/12o (right panel). (**b**) Evolution of regression weights for area 47/12o in response to the trials on which the PD-probability option was chosen compared to the trials on which the ED-option was chosen. (**c**) Evolution of regression weights of area 45a based on a multiple regression of expected performance (left) or coherence of the visual stimulus (right). Although performance systematically increases with the coherence level of the PD-probability option, area 45a was active was related to the PD-probabilty rather than directly to the coherence level. Shade: s.e.m across sessions.

In a final set of analyses of the fMRI data, we considered whether the activity that we had found in either area 45a or in area 47/12o could simply be related to the probability of reward associated with a choice or to some linked form of attentional modulation; adjacent areas of macaque ventrolateral prefrontal cortex have previously been linked to attentional selection^22^. If activity in either area 45a or area 47/12o simply reflects reward expectation at the time of choice during the first judgement, then it should correlate similarly with both PD- and ED-probabilities. However, several results demonstrate that this is not the case. First, as demonstrated in Extended Data Fig. 6a, the signal in area 45a was modulated by the PD-probability but not by ED-probability even though PD- and ED-probabilities have a linear relationship with subjective reward probabilities (Fig. 2a) in a similar manner. Therefore, the significantly larger modulation by PD-probability cannot be explained by reward expectation per se, and instead it is necessary to consider how the animal comes to estimate a particular level of reward; it does this by considering its own future performance levels.

Analogously, the signal in area 47/12o cannot be explained by a unitary mechanism of reward expectation. Activity in 47/12o was significantly larger in response to the ED-probability when it was chosen than it was to the PD-probability when it was chosen despite the reward probabilities being matched across these conditions. Again, therefore, activity patterns in area 47/12o also cannot be explained by reward expectation per se. Moreover, the double dissociation between the activity patterns in the two areas, areas 45a and 47/12o, cannot be explained without the existence of dissociable processes for making prospective judgements about PD- and ED-probabilities.

An alternative argument might, however, be made that neural modulations in area 45a or 47/12o simply reflect attention during the first judgement stage of each trial where attentional demands are defined in a different way: as a function of how much attention should be paid to the stimulus array in total, which, in turn, should be predicted by the sum of the subjective probabilities of reward associated with the PD- and ED-options. We estimated the subjective probability (from the proportion of trials on which each PD- or ED-option was chosen during the first judgement phase of each trial; Extended Data Fig. 2a). Consistent with predictions from prospect theory^23^, both PD- and ED-probabilities were underestimated when they were high and overestimated when they were low, but the mapping to subjective probability was different for the two types of option. As demonstrated in Extended Data Fig. 2b, neither area 45a nor area 47/12o was active in correlation with the summed subjective probabilities of both options, which should determine general attentional effects.

In a similar vein, it might be argued that area 47/12o activity simply increases as more dots are perceived on the screen because higher ED-probabilities are associated with more dots. However, an account of area 47/12o activity simply in terms of the dot number perceived would predict that that there would be more activity when the PD-option was chosen (because it contained 100 dots rather than the 10–90 dots contained by the ED-option), but this was not the case (Extended Data Fig. 6b). In the prospective judgement task, task performance systematically improved when the motion coherence increased. To directly test whether raw stimulus coherence or the value (expected reward) of average performance at each motion coherence level provides a better fit to area 45a activity, we conducted a multiple regression analysis using both coherence and performance as independent predictors of the signal time course during the first task-selection stage of each trial. The signal in area 45a was modulated by the difference between PD-probability associated with the chosen option and the ED-probability associated with the unchosen option (Extended Data Fig. 6c, left), but it was not modulated by the difference between the raw coherence level versus the ED-probability associated with the unchosen option (Extended Data Fig. 6c, right).

### Causal effects of disruption of areas 45a and 47/12o on prospection of future performance- and environment-dependent contingencies

**Fig. 6:**
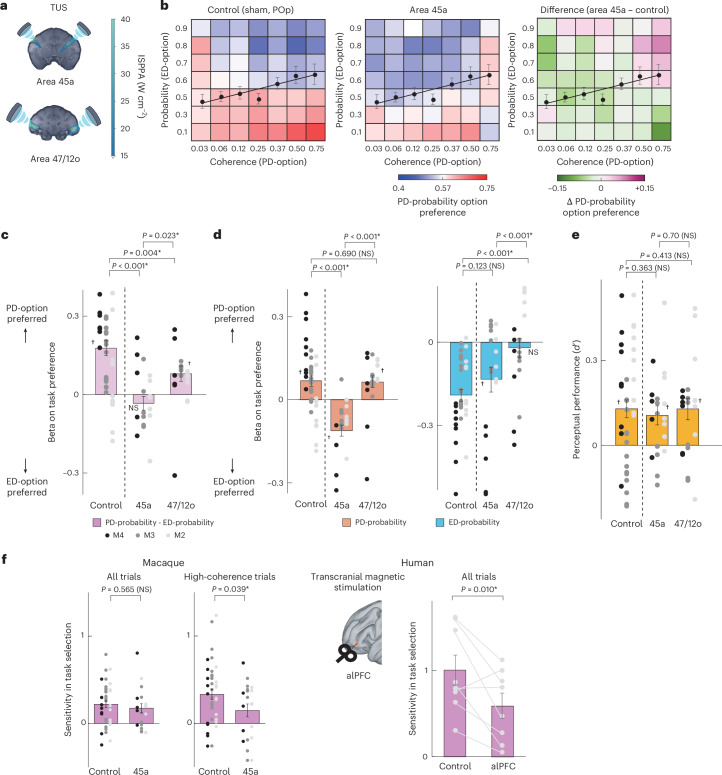
Causal effects of ultrasonic disruption of areas 45a and 47/12o on prospective judgements of future PD- and ED-probabilities. **a**, To test the causal necessity of the activated areas for prospective judgements, we applied TUS to bilateral area 45a (top) or 47/12o (bottom). Estimates of acoustic intensity at the TUS focus by acoustic simulation toolbox (k-Plan) are illustrated. In general, the maximum area of intensity (light green) was in the cortex in the target area (orange circle). The part of area 45a that was targeted in the right hemisphere was in the arcuate sulcus, and the area of maximum intensity was in area 45a in the immediately adjacent inferior convexity (see also Extended Data Fig. 4a). For control, we applied sham stimulation or bilateral POp stimulation, which is not involved in the task. **b**, Preference for choosing the PD-probability option in the prospective judgement stage after area 45a TUS (middle) and control condition (sham and POp stimulation, left). The differences between area 45a TUS and control (right) show that animals chose the PD-option less frequently, even when it was optimal to do so (below the black line), after area 45a TUS. **c**, Beta coefficients summarizing the influence of the difference in ED-probability associated with the left and right options in logistic regression analysis to predict choices of the left option choice in the environment-versus-environment task (lower part of Fig. 1b) for control condition, area 45a stimulation and 47/12o stimulation. After 47/12o stimulation, the performance deteriorated compared with control, but the difference between the effects of area 45a and 47/12o TUS was not significant. **d**, Beta coefficients of PD- and ED-probability (left) or the difference and sum of PD- and ED-probabilities (right) in logistic multiple regression analysis predicting choices of the PD-option as opposed to the ED-option in the control condition, after area 45a TUS and after area 47/12o TUS. The effects of TUS of areas 45a and 47/12o are doubly dissociable from one another, indicating distinct contributions to prospective evaluation of PD- and ED-probability, respectively. **e**, Absence of influence or either area 45a or 47/12o TUS on the perceptual decision-making task in the second stage of trials even when the PD-option was chosen (as evaluated by *d*′). **f**, Interspecies comparison of changes in sensitivity in task selection (PD-option task or ED-option task) due to disruption by area 45a TUS in macaques (left) and by alPFC TMS in humans (right). Area 45a TUS led to reduced rates of choosing the PD-option in macaques, and this reduction was significant when the motion coherence associated with the PD-option was high (see Fig. 1d showing that, at baseline, PD-option selection was clear and reliable when the PD-option had a high motion coherence). On these trials, the effect of area 45a TUS in macaques was comparable with the effect of alPFC TMS in humans on all trials. *N* = 80 sessions (3 animals). Each dot represents single-session data. Two-way ANOVA (TUS conditions and animals) are used for statistical tests of beta. Paired *t*-tests (two-sided) are used for statistical tests of sensitivity in task selection. †*P* < 0.05, *t*-test against zero with Bonferroni correction (two-sided). Error bar: s.e.m. across sessions. NS, not significant.

**Extended Data Fig. 7:**
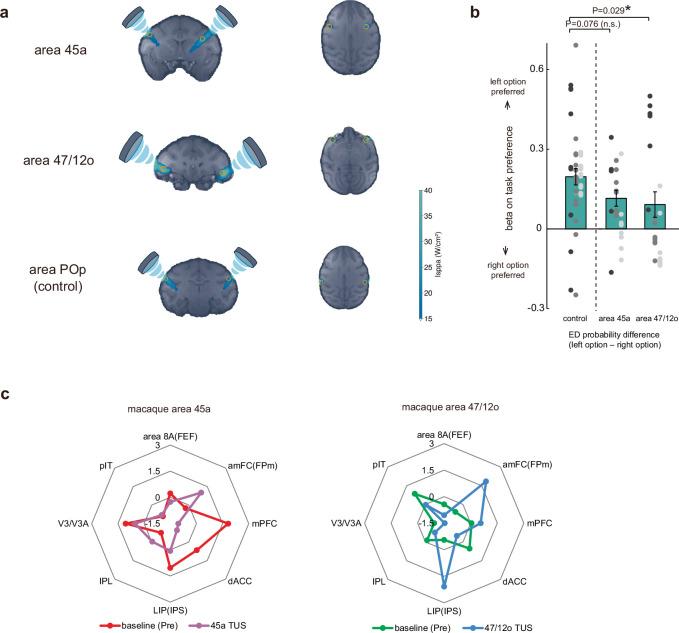
Simulated acoustic intensity of ultrasonic disruption and its causal effects on comparisons of ED-probabilities. (**a**) Simulation of the acoustic intensity of the TUS focus and trajectory in area 45a (top), area 47/12o (middle), and area POp (bottom) by acoustic simulation toolbox (k-Plan, Brainbox, UK). (**b**) Beta coefficients summarizing the influence of the difference in external probability associated with the left and right options in logistic regression analysis to predict choices of the left option choice in the external vs. external task (see Extended Data Fig. 1b). After 47/12o stimulation, the performance deteriorated compared to control. N = 50 TUS sessions. Error bar: s.e.m across sessions. (**c**) (Left) Causal effect of TUS on target brain region activity. There were changes in each region’s fc with eight key task-related regions (Extended Data Table 1) due to TUS targeted to the bilateral area 45a (left panel; see also panel **a**, top row) and area 47/12o (right panel; see also panel **a**, middle row). Fc-fingerprints of two sessions from each of two animals were compared between PRE- and POST-TUS. area 45a TUS decreased fc with mPFC, dACC and IPS whereas 47/12o TUS decreased fc with pIT and FEF and increased fc with amFC(FPm) and LIP(IPS). These dissociations in changed functional connectivity delivered by 45a and 47/12o TUS confirm that TUS could altered activity in each targeted area and that the spatial extent of intervention was sufficiently focal to dissociate areas 45a and 47/12o.

Next, to examine the causal role of areas 45a and 47/12o in predicting future PD- and ED-probabilities, we focally disrupted each of the two areas using transcranial ultrasound stimulation (TUS) (*N* = 80 sessions from three animals; Fig. 6a and Extended Data Fig. 7a). We examined how preference for the PD-probability option was altered by bilateral area 45a or 47/12o TUS in contrast to control (Fig. 6b). For the TUS experiments, we returned to using the two versions of the task in which animals either chose between ED-options (Fig. 1b, bottom, and Extended Data Fig. 1a) or between a PD- and an ED-option (Fig. 1b, top, and Fig. 1c). When animals compared two ED-options, there was a significant difference between 47/12o disruption and control (two-way ANOVA of disruption sites and animals; main effect of disruption sites (47/12o or control), *F*_1,58_ = 4.99, *P* = 0.029, *ηp*^2^ = 0.079, 95% CI 0.006 to 0.196; Extended Data Fig. 7b), and there was no significant difference between area 45a TUS and control (two-way ANOVA of disruption sites and animals; main effect of disruption sites (45a or control), *F*_1,57_ = 3.25, *P* = 0.076, *ηp*^2^ = 0.054, 95% CI 0.000 to 0.161). While this is consistent with the fMRI results that indicated area 47/12o encodes the ED-probability of reward associated with a choice regardless of whether or not that option is ultimately chosen (Fig. 4a,b), it is important to note that there are limitations to the strength of the findings; there was no significant difference between area 47/12o TUS and 45a TUS (two-way ANOVA of disruption sites and animals; main effect of disruption sites (45 or 47/12o), *F*_1,57_ = 0.22, *P* = 0.64, *ηp*^2^ = 0.004, 95% CI 0.000 to 0.077).

By contrast, when we examined the results on the other trials, when animals chose between PD- and ED-options, clear differences were found between area 45a TUS and area 47/12o TUS. We compared the beta coefficients indexing the influence of PD- and ED-probabilities on selection of the PD-option after either area 45a or 47/12o disruption and in the control condition (Fig. 6d). In line with the fMRI results, we found that areas 45a and 47/12o made doubly dissociable contributions to evaluation of the two types of probability. The impact of PD-probability on PD-option preference was significantly affected by area 45a disruption compared with area 47/12o disruption (two-way ANOVA of disruption sites and animals; main effect of disruption site: *F*_1,35_ = 42.4, *P* = 1.6 × 10⁻^7^, *ηp*^2^ = 0.548, 95% CI 0.379 to 0.680), whereas the effect of ED-probability was significantly affected by area 47/12o disruption compared with area 45a disruption (two-way ANOVA of disruption sites and animals; main effect of disruption site: *F*_1,35_ = 21.0, *P* = 5.6 × 10⁻^5^, *ηp*^2^ = 0.375, 95% CI 0.200 to 0.536). The distance between the TUS foci for area 45a and area 47/12o was 12.5 mm in each hemisphere. This double dissociation suggests that the spatial range of the TUS effects on behaviour is much narrower than 12.5 mm.

To compare area 45a TUS, 47/12o TUS and the control condition, in terms of a single index, we examined the influence of the difference between PD- and ED-probabilities on each trial (Fig. 6c) just as we had done previously (Fig. 1e). The impact of the difference between PD- and ED-probabilities was significantly reduced by area 45a disruption compared with area 47/12o disruption (two-way ANOVA of disruption sites and animals; main effect of disruption sites (45a or 47/12o), *F*_1,35_ = 5.62, *P* = 0.023, *ηp*^2^ = 0.138, 95% CI 0.013 to 0.314). These findings suggest that area 45a is causally necessary for accurate prospective judgements regarding which tasks to attempt in the future that are made on the basis of estimates of PD-probability. It is especially concerned with evaluating PD-probability (Fig.6d) but also with comparing estimates of PD-probability with estimates of ED-probability that were also linked to 47/12o; again, these results are consistent with fMRI results (see also Fig. 2a,b).

As the control condition, both sham stimulation (passive control) and parietal opercular cortex stimulation (active control) were applied to all animals. Because the behavioural effects of the two control conditions were comparable, we merged these two conditions as the baseline against which to assess area 45a and 47/12o stimulation effects. Even when only the sham condition is used as a control, the main observations and conclusions remained the same. The positive influence of the ‘difference between PD- and ED-probability’ was significantly decreased by area 45a TUS compared with sham (*F*_1,37_ = 25.62, *P* = 1.1 × 10⁻^5^, *ηp*^2^ = 0.409, 95% CI 0.238 to 0.563). The positive influence of PD-probability in sham was reversed by area 45a TUS (*F*_1,37_ = 63.80, *P* = 1.4 × 10⁻^9^, *ηp*^2^ = 0.633, 95% CI 0.476 to 0.740), and the negative influence of ED-probability was diminished by area 47/12o TUS (*F*_1,38_ = 24.89, *P* = 1.3 × 10⁻^5^, *ηp*^2^ = 0.396, 95% CI 0.228 to 0.550).

Despite these changes in prospective judgements about which tasks to tackle, it is noteworthy that neither area 45a nor 47/12o disruption influenced the subsequent first-order perceptual decisions during the final stage of trials (two-way ANOVA of disruption sites and animals; main effect of disruption sites (45a, 47/12o or control), *F*_2,78_ = 0.35, *P* = 0.70, *ηp*^2^ = 0.009, 95% CI 0.000 to 0.058; Fig. 6e). Areas 45a and 47/12o prospectively evaluate performance-dependent and environment-dependent contingencies, but, subsequently, they are less critical for the execution of the perceptual decision task per se.

It is notable that area 45a TUS did not simply reduce the normal influence of the PD-probability but actually made animals averse to choosing that option. Area 45a TUS appeared to compromise assessment of the PD-probability and, in the absence of a reliable PD-probability estimate, animals may have become averse to selecting the PD-probability option. Before TUS, animals could use the estimate of PD-probability to choose the optimal task option most effectively when the subsequent perceptual task in the final stage of the trial was relatively easy and PD-probability was high (Fig. 1d). Thus, the influence of area 45a disruption was especially prominent for the trials with a high PD-probability. When the PD-probability was low, as noted, the influence of the PD-probability was less substantial even in the absence of TUS, and this did not change after area 45a TUS. Therefore, area 45a disruption caused an avoidance of choosing the high PD-probability option on trials when it was offered without altering the animals’ ambivalence for the low PD-probability option. The net result is the negative modulation of the PD-probability on selection of that option after area 45a disruption. Relatedly, the impairment in task selection due to area 45a disruption compared with control was especially apparent in high-coherence stimulus trials (Fig. 6f, left). On these trials, the effect of area 45a TUS in macaques was comparable to the effect of alPFC transcranial magnetic stimulation (TMS) in humans on all trials (Fig. 6f, right). These observations suggest a similar role between macaque area 45a and human alPFC even though the suggested mechanisms enabling prospective judgements are different between species (see also Fig. 5c,f).

### Interspecies commonalities and differences in prospection between macaques and humans based on functional connectivity network patterns

In the present study, we demonstrated that macaque areas 45a and 47/12o enable prospective evaluation for future planning. We previously found that, in humans, the alPFC (area 47) was active and causally essential for prospective matching of PD- and ED-probabilities^5^. There are many similarities between macaque and human prefrontal cortex, and there are parts of closely adjacent divisions of areas 45a and 47 that resemble one another in the two species^24,25^. However, within this context of broad interspecies similarity, the alPFC is one of the areas that is most distinctive in humans. Compared with many other human prefrontal areas, human alPFC’s pattern of functional connectivity (fc patterns) is less like that of any macaque brain area^6^. Other comparisons of prefrontal connectivity in humans and macaques also suggest the human alPFC region is distinctive^26,27^. The fc pattern is one good approximation of the strength of anatomical connections between area pairs albeit taking into consideration the effects of indirect multisynaptic anatomical connections as well as monosynaptic, direct connections, which are often correlated with one another^28–31^. However, despite the absence of a macaque area that is homologous in every aspect of its fc patterns, our current findings suggest that the combination of areas 45a and 47/12o play a related functional role in macaques and that these areas enable macaques to engage in prospection and metacognition. One possibility is that, while macaque area 45a might resemble human area 45a and macaque area 47/12o might resemble human area 47/12o, perhaps human alPFC has features of both macaque areas 47/12o and 45. Such a possibility would be in line with the suggestion that one way in which a new area can arise in one species in comparison with another is if, in the first species, there is duplication of what is a single area in the second species. In addition, in the first species, there may be a spreading of connections so that the new duplicated area does not just receive the inputs it originally did but also connections from other nearby areas^7,32^. In other words, human alPFC may have arisen from the duplication of a ventrolateral prefrontal area and, in addition to having the connections of macaque area 47/12o, it may also have the connections associated with other nearby areas such as 45a.

**Fig. 7:**
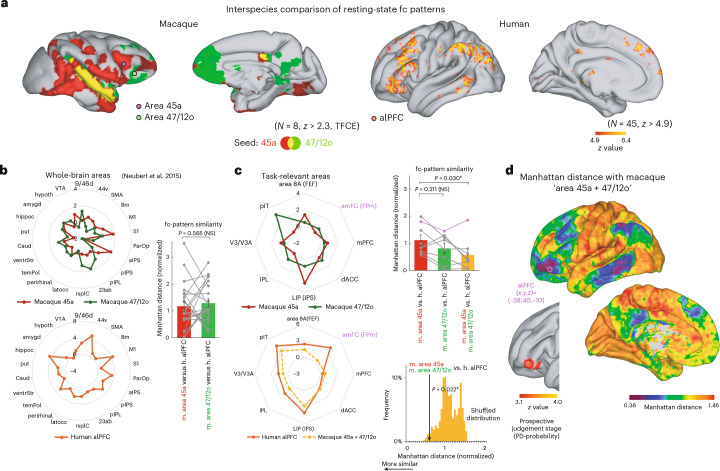
Distributed neural networks linked to macaque areas 45a and 47/12o and human alPFC: interspecies commonalities and differences. **a**, Interspecies comparison of resting-state fc patterns for areas 45a (red) and 47/12o (green) in macaques (*N* = 8) (left) and alPFC (orange) in humans (*N* = 45 from the HCP^67^) (right). **b**, Left: each region’s fc with 22 regions, which were determined based on areas identified by Neubert et al.^6^ and which were irrelevant to performance of the task investigated, is summarized as an fc-fingerprint, separately for macaques (top) and humans (bottom). Right: Manhattan distance between the fc-fingerprint of macaque (m) areas 45a or 47/12o and the fc-fingerprint of human (h) alPFC. The magnitude of functional connectivity was normalized within each area. Smaller Manhattan distance indicates stronger interspecies similarities in the paired areas. **c**, Left: each region’s fc with eight key regions, which were determined based on the brain activity during the current task, especially activity emerging at the decision stage in the second part of each trial (Extended Data Table 1). This is summarized as fc-fingerprints for macaques (top) and humans (solid orange line, bottom). We also illustrate an fc-fingerprint formed by combining across both macaque areas 45a and 47/12o (dashed yellow line). Top right: Manhattan distance between the fc-fingerprint of macaque areas 45a, 47/12o or 45a + 47/12o (inclusive) and the fc-fingerprint of human alPFC. The fc-fingerprint formed by the 45a + 47/12o combination closely matched the fc-fingerprint of human alPFC except for the frontopolar cortex (area 10, FPm) (purple trace). Bottom right: assessment of the statistical significance of the Manhattan distance between macaque area 45a + 47/12o and human alPFC based on the shuffling of fc patterns (1,000 times). The observed Manhattan distance was the bottom 2.2% of the shuffled distribution. **d**, Map of human brain voxels where the connectivity pattern is similar to the connectivity pattern of the combination of macaque areas 45a and 47/12o (the distance between connectivity patterns was the lowest). The most similar voxel was identified at (*x*, *y*, *z*) = (−38, 42, −6) (distance 0.26) within the human alPFC region (*z* = 4.16, *P* < 0.05, cluster-level corrected, peak at (*x*, *y*, *z*) = (−38, 40, −10)). Each grey dot and line represents single area data (**b**,**c**). Paired *t*-tests (two-sided) with Bonferroni correction are used for statistical tests. Error bar: s.e.m. across areas.

**Extended Data Fig. 8:**
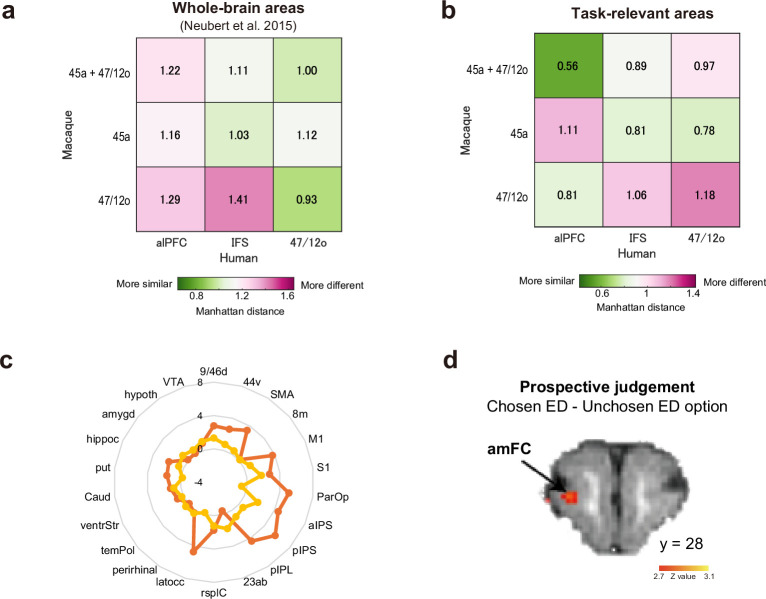
Similarity of functional connectivity patterns in humans and macaques. (**a**) Similarity of functional connectivity patterns between three macaque areas (area 45a, area 47/12o and a combined area 45a-47/12o) and three human areas (alPFC, area 45a in the inferior frontal sulcus [IFS] and area 47/12o). Human IFS and area 47/12o were considered the anatomical homologues of macaque area 45a and area 47/12o, respectively, by Neubert et al.^6^ and this was apparent again in the similarities of their connectional fingerprints. To calculate the Manhattan distance between connectional fingerprints, we examined each region’s functional connectivity with 22 regions identified by Neubert et al.^6^ (see also Fig. 7b). On the basis of these 22 areas, human alPFC and the macaque combination of areas 45a and 47/12o did not resemble one another. (**b**) Similarity of functional connectivity patterns between the macaque and human for the same brain areas as in panel **a**. Now, however, Manhattan distance between connectional fingerprints was calculated based on each region’s functional connectivity with eight key regions, which were determined based on the brain activity during the current task, especially activity emerging at the decision stage in the second part of each trial (Extended Data Table 1) (see also Fig. 7c). Although now the similarity between macaque and human 45/IFS and between macaque and human 47/12o are less clear, the similarity between human alPFC and the. (**c**) fc-fingerprint with task-irrelevant 22 regions, which were determined based on areas identified by Neubert et al.^6^ for the combination of both areas 45a and 47/12o in macaque (yellow) and alPFC in the human (orange). (**d**) The frontopolar (area amFC) signal in macaques is focal and prominent in response to ED-probability rather than PD-probability (Z = 3.19, p = 0.00071) although it did not survive the conservative statistical threshold imposed by cluster-level correction.

Therefore, we directly compared whole-brain fc patterns of macaque areas 45a and 47/12o and human alPFC^29^ (Fig. 7a). Based on fc patterns with task-irrelevant areas^6^, macaque area 45a, which mediates prospective evaluation of PD-probability, was no more or less similar to human alPFC than macaque 47/12o, which encodes prospective ED-probability (Manhattan distance, *t*_21_ = 0.58, *P* = 0.565, *r* = 0.126, 95% CI −0.310 to 0.517; Fig. 7b). Next, we carried out a similar analysis but now focusing on the areas’ fc patterns with only other task-related, as opposed to task-unrelated, areas (Extended Data Table 1), especially areas for perceptual decision-making. The homologies between human and macaque areas for the target regions of fingerprint spider plot, especially where prominent cortical expansion happened and anatomical structures exhibit some interspecies divergence, are critical for the interspecies comparisons of fc-pattens. Using target regions identified by task-based fMRI during performance of similar prospective judgements and perceptual decisions in both macaques and humans ensures their functional correspondence. Again, macaque area 45a was no closer to human alPFC than macaque 47/12o (*t*_7_ = 1.85, *P* = 0.106, *r* = 0.573, 95% CI −0.155 to 0.885). However, the fc-network for the combination of both macaque areas 45a and 47/12o was very similar to the human fc-network centred at alPFC (Fig. 7c left, top and bottom). The fc pattern of the combined macaque areas 45a-47/12o (Methods) matched human alPFC’s fc pattern significantly (*P* = 0.022 against shuffled data, Fig. 7c, bottom right). The fc pattern of macaque area 45a became significantly closer to human alPFC’s fc pattern if the contributions of fc patterns with macaque area 47/12o were also included (*t*_7_ = 3.19, *P* = 0.015, *r* = 0.770, 95% CI 0.244 to 0.944; Fig. 7c, top right). By contrast, the fc pattern of the combined macaque areas 45a-47/12o did not match significantly with adjacent but more ventral and posterior areas in the human brain, human 47/12o’s fc pattern (*P* = 0.33 against shuffled data) or human area 45/IFS’s fc pattern (*P* = 0.11 against shuffled data). These more ventral and posterior human areas, 47/12o and 45/IFS, have fc patterns that are known to be similar to the fc patterns of macaque area 47/12o^6^ and area 45a^7^, respectively (Extended Data Fig. 8a–c provides further demonstrations of similarity between human and macaque 47/12o, between human and macaque 45a and between human alPFC and the combination of macaque 45a and 47/12o). To causally test if functional connectivity networks from the seed at macaque 45a and 47/12o are segregated from each other, we applied bilateral TUS to either area 45a or 47/12o and compared the resting-state functional connectivity networks^33–37^ between pre-TUS and post-TUS scans (four sessions in total from two animals (M2 and M5) for each TUS target). 45a TUS prominently decreased area 45a’s functional connectivity with mPFC, dACC and LIP (IPS) compared with the pre-TUS baseline (Extended Data Fig. 7c, left). By contrast, TUS targeted to area 47/12o prominently decreased 47/12o’s functional connectivity with area 8 A (FEF) and pIT (Extended Data Fig. 7c, right). These dissociations in altered functional connectivity profiles after 45a and 47/12o TUS support the claim that TUS of either 45a or 47/12o modulates separate subnetworks of the brain in macaques. These segregated TUS-evoked changes suggest that the spatial range of the TUS effects was narrower than half the distance between the areas 45a and 47/12o (12.5 mm/2 = 6.25 mm). The nature of the ultrasound waves ensured that the area of high-intensity ultrasound stimulation was in a spindle shape (Extended Data Fig. 7a). However, we applied the ultrasound stimulation perpendicularly to the cortical layer and the most sonicated areas in the longitudinal direction were the targeted cortical areas themselves and then, secondarily, the adjacent white matter regions carrying connections to and from the targeted areas.

In summary, when we compared the fc patterns of human and macaque prefrontal areas with a selective group of other task-related regions, we were able to find a clear similarity between the FC of human alPFC and the combination of macaque areas 45a and 47/12o. However, when we compared the FC of human alPFC and the combination of macaque areas 45a and 47/12o with a larger but less selective group of brain that were task-unrelated (Extended Data Fig. 5d), differences were apparent. While any interpretation of the differences should be made very cautiously, the greater connectivity between human alPFC and several parietal cortex (pIPL, pIPS, aIPS and ParOp) might be consistent with human alPFC having access to wider range of information processed in other brain areas than is the case for the area 45a and 47/12o combination in macaques, and this might contribute to the more profound introspective ability found in humans.

In a final analysis, we looked across the entire human brain to see which human brain area was the best match to the combination of macaque area 45a and 47/12o based on fc patterns with task-related areas. The voxel-based whole-brain analysis calculated the Manhattan distance^6,7,38^ between each human brain voxel’s connectivity pattern and the connectivity pattern associated with macaque area 45a + 12/47o combination. The resulting map specifies how similar every voxel in the human brain is to the combination of areas 45a and 47/12o in the macaque. The blue cluster (Fig.7d) indicates the human brain voxels where the connectivity pattern is most similar to the connectivity pattern of the combination of macaque areas 45a and 47/12o (the distance between connectivity pattern’s was lowest). The most similar voxel was identified at (*x*, *y*, *z*) = (−38, 42, −6) (distance 0.26) within the human alPFC region (*z* = 4.16, *P* < 0.05, cluster-level corrected, peak at (*x*, *y*, *z*) = (−38, 40, −10)) that is responsive to PD-probability during the prospective judgement stage.

In summary, the similarities between the combined roles of macaque areas 45a and 47/12o and human alPFC in prospective judgement may reflect that, in addition to 45a and 47/12o, humans have a new alPFC area that resembles a combination of macaque areas 45a and 47/12o.

**Fig. 8:**
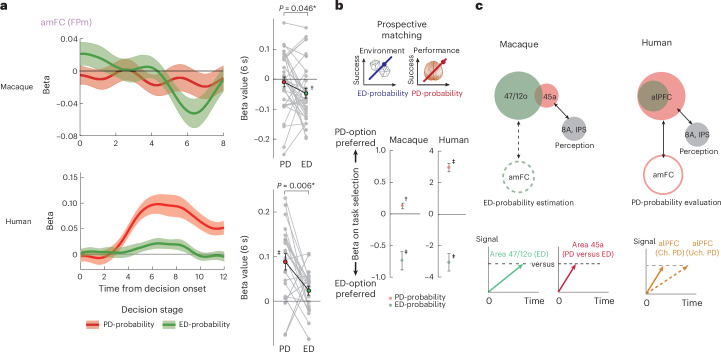
Interspecies differences in the role of the frontopolar cortex and proposed neural circuit model for prospective metacognition. **a**, The evolution of regression weights across time in FPm during the final perceptual decision-making stage in macaques (top) and humans^5^ (bottom) separately for trials when the PD-option (red) and ED-option (green) had been chosen. In humans, FPm more strongly represents PD-probability than ED-probability, whereas, in macaques, FPm more strongly represents ED-probability than PD-probability, as shown in the comparisons of betas 6 s after event onset (right). **b**, Interspecies comparisons of the influence of PD- and ED-probabilities on the selection of the PD-option in macaques (left) and humans^5^ (right). Humans relied more on PD-probability. **c**, A proposed interspecies model based on the results from the present study. A double dissociation between areas 45a and 47/12o was found in macaques, reflecting complementary roles in prospective judgements about different types of probability (PD and ED) (left), whereas in humans, alPFC—strongly connected with FPm—assesses and compares both probability types (right). Each grey dot and line represents single-session data for macaques and single-participant data for humans. Paired *t*-tests (two-sided) are used for statistical tests. ‡*P* < 0.001, †*P* < 0.05, *t*-test against 0 with Bonferroni correction (two-sided).

The only exception to the broad correspondence between human alPFC fc and the fc of the macaque 45a-12/47o combination concerned anterior medial frontal cortex (amFC; area FPm)^4,39–43^. Whereas human alPFC activity exhibits significantly positive correlation with human amFC (*P* < 0.05, corrected by threshold-free cluster enhancement (TFCE)), neither macaque area 45a nor 47/12o exhibits strong connectivity with macaque amFC (see also purple trace in Fig. 7c, right). The activity of amFC was linked to perceptual decision-making in the second decision stage in both macaques and humans. However, the activity patterns it expressed when doing so were different in the two species: in humans, amFC activity is more correlated (positively) with PD- than ED-probability (*t*_21_ = 3.00, *P* = 0.0066; Fig. 8a, bottom), whereas in macaques, amFC activity (Extended Data Fig. 8d) is more strongly correlated with ED-probability (negatively) than PD-probability (*t*_34_ = 2.06, *P* = 0.046; Fig. 8a, top). The strong pattern of interaction between human alPFC, which is linked to second-order prospective judgements at the first stage of each trial, and amFC, which is linked to PD-probability encoding for first-order decisions at the second perceptual decision stage of the trial, which is absent in macaques, may partly explain why prospective judgements about future contingencies in humans are more strongly informed by insight into PD-probability than is the case in macaques (Fig. 8b; see also Fig. 1c).

## Discussion

Humans are able to evaluate their own future performance levels and future ED-probabilities^5,14^. As a result, they are able to make fine judgements about which tasks to tackle based on accurate prospective assessments of how likely they are to succeed. When humans make such prospective judgements, a region in ventral, alPFC is active^5^; alPFC activity increases as a function of how likely the person estimates their future performance is to be successful. Although specialized for evaluating and ultimately choosing options on the basis of assessment of PD-probabilities, human alPFC also reflects information about environmental contingencies. Prospective estimates of both future performance and future environmental contingencies may be integrated in alPFC and influence the tasks that people decide are worth attempting (Fig. 8c, right).

Intriguingly, in humans, the ability to prospectively evaluate future performance levels depends on separate neural mechanisms from those concerned with actual performance of the behavioural task itself; activity in alPFC did not encode decision variables when perceptual decisions were made a few seconds after the prospective evaluation of whether or not to attempt the perceptual decisions. Similarly, disruption of alPFC activity impairs people’s judgements about which decisions to attempt but does not disrupt the subsequent perceptual decision-making itself.

An unusual feature of human alPFC, however, is that it is one of the prefrontal regions that, in terms of the functional networks with which it is associated, is most different to macaque prefrontal cortex. It is important to emphasize that there are fundamental similarities between prefrontal cortex in humans and macaques^24,44^, but, nevertheless, within this picture of broad similarity, the human alPFC is one of the regions that appears more distinct^6,26,27^. This has raised the question of whether primates such as macaques are able to prospectively evaluate their own future performance in the same way as humans and, if so, how—that is, via which neural mechanisms they do so^13^.

Macaques, like humans, are capable of prospective evaluation of their own future behavioural performance as well as of future environmental contingencies that are independent of their own behaviour (Fig. 1). However, while they are able to evaluate their own future performance levels, their ability to do so is weaker than in humans. It is, nevertheless, most apparent when prospective judgements are made about decisions of lower difficulty levels that macaques may assess more comprehensively (Fig.1d, right). At least this is the case in the present experiment, which required prospective evaluation of future performance to be contrasted with prospective evaluation of environmental contingencies.

Moreover, analyses of the brain activity linked to the making of prospective judgements of future performance levels suggested important differences between macaques and humans. Macaque prospective judgements concerning their own performance and environmental contingencies depend on doubly dissociable and complementary activity patterns in areas 45a and 47/12o (Figs. 2, 3 and 8c, left). The region of activity in area 47/12o was near to one that has been linked to probability evaluations in other contexts^21,45^. We note that, while activations sometimes only exceeded the spatial cluster correction threshold in one hemisphere, similar patterns of activity were usually evident in both hemispheres below threshold.

The region of activity in area 45a lay anterior to the ventral branch of the arcuate sulcus. Thus, it was distinct from the FEF just anterior to the bow of the arcuate sulcus where activity was prominent when macaques made the actual perceptual decisions. In area 45a, there was activity linked to prospective metacognitive judgements in the first stages of trials but also, nearby, activity linked to the subsequent perceptual decisions. By contrast, in the FEF, we found only activity linked to the perceptual decisions. TUS disruption of macaque area 45a altered prospective evaluations of future performance levels without compromising how the decisions themselves were made (Fig. 6). By contrast, TUS of 47/12o compromised the assessment of ED-probabilities (Fig. 6). Nevertheless, it may be important that area 45a has monosynaptic connections with cortex in and near both the frontal and supplementary eye fields^25,46,47^. Connections with visual decision-making circuits may enable it to estimate PD-probabilities.

In summary, prospective judgements about future performance in a decision-making task depend on the prefrontal cortex in both humans and macaques, but the critical regions within prefrontal cortex are different. Nevertheless, in both humans and macaques, the prefrontal regions concerned with prospective evaluation of future decisions are distinct from brain regions engaged during perceptual decisions themselves.

Functional divergence in neuroanatomy across species may arise in a number of ways, but they include, in one species, duplication of what is a single area in another species, as well as the spreading of connections that are found in one area in one species to adjacent areas in another species^32^. Such changes appear to have occurred as humans and macaques diverged from a common ancestor approximately 25 million years ago^44^. Neither macaque area 45a nor macaque area 47/12o resembles human alPFC in terms of its functional interaction patterns with other brain areas (Fig. 7). Functional interactions between brain regions reflect the presence of anatomical connections between brain regions^28^, and so the differences in functional connectivity between macaque areas 45a and 47/12o and human alPFC suggest they have different anatomical connections. However, the connectivity pattern associated with the combination of macaque areas 45a and 47/12o closely resembled the connectivity pattern of human alPFC (Fig. 7). In fact, when we examined the connectivity patterns of every voxel in human prefrontal cortex, we found that the connectivity pattern associated with the combination of macaque areas 45a and 47/12o more closely resembled the connectivity pattern in voxels in human alPFC than it did anywhere else in the entire human brain (Fig. 7c). We propose that, since the time of their common ancestor, the cortical region corresponding to area 47/12o in macaques has probably expanded in humans over the course of human evolution. As a result, anatomical projections that may have been confined to area 45a in macaques may extend beyond area 45a into adjacent areas such as area 47/12o in humans. A part of 47/12o might have been ‘colonized’^48^ by these connections and developed as human alPFC, while other human areas that are also successors to an ancestral 47/12o region may not have been colonized by projections associated with area 45a in the macaque. We emphasize that we are not proposing, however, that areas 45a and 47/12o, as they exist in the macaque, have become fused in the human brain. In other words, if alPFC is indeed an evolutionarily novel area unique to humans and with a specialized function, macaques cannot perform the task by activating a single homologue of human alPFC if they do not possess it. Instead, we should expect that, if macaques are able to perform the task at all, then they may well do so via the assistance of activity patterns in more than one of the less specialized brain areas they do possess, such as areas 45a and 47/12o.

Caution is needed in interpreting resting state functional connectivity data as they reflect a mixture of monosynaptic and polysynaptic anatomical connections^28^. However, in this study, by comparing identically derived functional connectivity measures in the two species we are able to establish some evidence that the areas in the two species—areas 45a and 47/12o—are each a part of a similar circuit in both species. An additional caveat when making comparisons of the functional connectivity of two areas across species is that the comparison is based on the assumption that the other areas from which functional connectivity measures are taken—for example, the areas on the circumference of the spider plots in Fig. 7b—are similar across species. In the current study, we have tried to focus on areas where there is evidence for such correspondences, but it is important to consider other lines of evidence for cross-species homology such as the task-related activity patterns and the TUS/TMS-induced patterns of impairment seen when the areas in the two species are stimulated.

The weaker capacity for prospective judgements concerning future performance that is seen in macaques might reflect the absence of a unitary anatomical locus for interactions between activity reflecting estimates of future performance and estimates of environmental contingencies. Instead, whether macaques took the PD-probability option or the ED-option reflected the relative timing of activity in areas 45a and area 47/12o. Activity linked to performance levels ramped up more quickly when macaques took that option than when they rejected it (Figs. 2, 3 and 5a–c). By contrast, the timing of activity linked to evaluation of ED-probabilities in area 47/12o did not change regardless of which choice was made. The combination of different brain activity patterns in the two areas meant that area 45a activity encoding the PD-probability option preceded (followed) the ED-option when the PD-options was chosen (rejected). We did not record the spiking activity of the neurons in these areas directly, but rather we used MRI to measure the haemodynamic responses that were triggered by neural activity. Such haemodynamic responses are, therefore, slower than the triggering neural activity. It should be noted that the slope and latency of the signal time courses that we report (Figs. 2b–d, 3 and 4b,c) reflect the degree of modulation by PD- and/or ED-probabilities on haemodynamic responses rather than simply the raw haemodynamic responses themselves. Thus, an earlier latency of a signal in such an analysis means that there was likely to have been spiking activity in the area that was sensitive to the estimation of that particular type of probability, which could be detected from a very early stage during the prospective judgement.

In humans, the ability to make judgements about one’s own future performance levels has been described as metacognitive in nature^5,13^. A similar mechanism is also used when people make social judgements about individuals other than themselves who have similar or inferior ability levels^14^. By contrast, judgements about the future performance levels of people with abilities that exceed our own cannot be made in the same way and do not depend on alPFC but on a distinct network of areas linked to behavioural observation as opposed to behavioural simulation. It may, therefore, be appropriate to consider the prospective judgements of macaques as metacognitive in nature^1,49^. Analysis of the challenge and inevitable trials (Fig. 2g) suggests that prospective judgements are not simply dependent on identifying the coherence level of an RDK pattern but instead on trial-by-trial assessments of the performance level the macaque is currently capable of. Neither activity in area 45a nor in 47/12o can be explained by reward expectation (Extended Data Fig. 6a) or attention allocation (Extended Data Fig. 2b). Nevertheless, prospective judgements about future performance of the type that we investigate here may be different in nature, not just to the decisions themselves^50^ but also to retrospective judgements^49^. In some cases, retrospective insights into specific instances of decisions that have already been taken and that might be recalled as individual episodic events may be linked to the neural processes first involved in making the decision^51–53^. In some cases, such retrospective judgements may be especially closely linked to the neural mechanism that took the decision. By contrast, the activity patterns found in areas 45a and 47/12o in macaques may provide a clue to the evolutionary neural origin of prospective suppositional thinking in human alPFC^13,54^.

## Methods

### Experimental model and study subject details

All macaque experiments were conducted in accordance with European Union Directive 2010/63/EU of the European Parliament on the protection of animals used for scientific purposes and with the UK Animals (Scientific Procedures) Act 1986 under licences approved by the UK Home Office. The research complies with all relevant ethical regulations for research with non-human animals, including the ARRIVE (Animal Research: Reporting In Vivo Experiments) guidelines.

All human study was approved by the Central Research Ethics Committee (fMRI experiment: MSD-IDREC-R51506/RE002, TMS experiment: R65502/RE001) at the University of Oxford. All participants (fMRI experiment: *N* = 23 (15 female); age (mean ± standard deviation (s.d.)), 28.2 ± 6.7. TMS experiment: *N* = 8 (5 female); age (mean ± s.d.), 25.8 ± 4.4) gave informed consent. Participants received £10 per hour and a bonus based on task performance (accumulated across sessions: £5–£7 per session).

#### Subjects

Four adult male monkeys (*Macaca mulatta*) were tested with behavioural tasks; three (M1, M2 and M3) performed the tasks in experiment 1 and experiment 2 while fMRI data were collected. Three (M2, M3 and M4) performed the task in experiment 3 in which the impact of TUS on behaviour was measured. They were aged between 7 and 10 years and weighed between 11 and 17 kg at the time of the experiments. They lived on a 12-h light–dark cycle, were fed once per day after testing and had ad libitum water access for an average of 15 h per day (and a minimum of 3 h per day).

Monkeys were trained to sit in a sphinx position in an MRI-compatible chair and to make arm-reaching movements towards custom-made infrared touch sensors to obtain a liquid reward from a tube positioned directly in front of their mouths. Initially, they were trained while the MRI-compatible chair was placed in a wooden custom-made mock scanner. After the animals became accustomed to working in this way, they were trained to work in an actual MRI scanner. Task stimulus images were displayed on a screen (24 inches diagonal, 1,000 mm away, centred at the monkey’s eye level). Animals underwent aseptic surgery to implant an MRI-compatible head post (Rogue Research). The monkeys were initially sedated with medetomidine and midazolam, and were then anaesthetized by isoflurane under mechanical ventilation throughout the surgery. Surgical treatments were performed after confirming the disappearance of the pain reflex. During anaesthesia, blood pressure, heart rate, SpO_2_ and end-tidal CO_2_ were continuously monitored to optimize ventilation and gas concentration. The monkeys were given postsurgical analgesics as well as postsurgical prophylactic antibiotics after the surgery for 1 week.

For fingerprint functional connectivity analysis, we performed a series of novel analyses but using data from a different set of eight male monkeys (*M. mulatta*)^55^. Additional human behaviour and fMRI data acquired during the performance of similar tasks^5^ are also re-analysed and presented here. Human functional connectivity analysis was based on preprocessed data provided by the Human Connectome Project (HCP)^56,57^.

### Method details

#### Behavioural tasks

We used Presentation software (Neurobehavioral Systems) to control the experimental task displayed on the screen and to receive triggers from the MRI scanner. Experiments 1, 2 and 3 used the same behavioural task. The two main tasks (performance-versus-environment task and environment-versus-environment task) comprised two stages: each trial comprised a prospective judgement followed by a perceptual decision and a final outcome phase (Fig. 1a). In the prospective judgement stage, animals had to choose one of the two RDK stimuli that were presented simultaneously. One RDK represented an ED-probability task, and the other represented either a similar ED-probability task (environment-versus-environment task) or a PD-probability task (performance-versus-environment task). Either stimulus could appear with the same frequency on the left or on the right of the screen. The PD-probability option contained a full array of dots (100 dots; the ED-probability of reward, indicated by the number of dots, was always 1) in white, but the motion of the dots was ambiguous (3%, 6%, 12%, 25%, 37%, 50% and 75% denote the different coherence levels). The ED-probability task contains a smaller number of dots (10, 30, 50, 60, 70, 80 and 90 indicating ED-probabilities of between 0.1 and 0.9 of reward) in blue, but all dots moved in the same direction (100% coherence), which was always easily discernible by every animal. However, note that the PD-probability task always comprised the full number of dots, and the ED-probability task always utilized 100% coherence, which means that always only one of the two dimensions varied per task type while the other one was fixed. All combinations of the seven ED-probability levels (environment-versus-environment task) or of the seven PD-probability levels and seven ED-probability levels (performance-versus-environment task) were offered during the first stage of each trial—the prospective judgement stage. In the second stage following this prospective judgement, animals were given a chance to perform the task they chose, in which they touched either a left or right sensor box depending on the direction in which most dots moved (the ‘coherent’ dot direction). The number of dots in the ED-probability task indicated the ‘environmental probability’ of receiving a reward when the correct response was made; if there were more (fewer) dots, then the probability of reward was higher (lower). This ED-probability was independent of the animal’s own performance levels. The coherence of the synchronized dot motions in the PD-probability task, however, determined the animal’s performance levels and, therefore, it determined the PD-probability of whether a reward would be received; they were more likely to perform correctly when coherence levels were high as opposed to low. To maximize the chance of reward to be obtained after the second perceptual decision stage, animals were required to compare the ED- or PD-probability of task options offered during the prospective judgement stage.

In the prospective judgement stage, each RDK stimulus was moving downwards. Stimuli were presented continuously until animals made a choice about the task that they wanted to perform in the subsequent perceptual decision stage by reaching with either the left or right arm. To ensure that animals had sufficient time to look at both RDK stimuli, animals were not allowed to respond too quickly (<500 ms); only responses made after the 500-ms deadline determined the second, perceptual decision stage of the trial. This second stage began after a stimulus onset asynchrony (SOA) (experiment 2, 2.5–7 s (Poisson distribution, mean of 3 s); experiment 3, 1 s; note that a longer SOA was needed in experiment 2 to ensure that blood oxygen level-dependent (BOLD) activity, which has a mean haemodynamic response function delay of 2–4 s, could be reliably separated and linked to component elements within each trial, but such long delays were not needed in experiment 3 when the behavioural impact of TUS disruption was investigated). During the SOA, the chosen stimulus moved to the centre of the screen and animals moved from the initial prospective judgement stage of the trial to the subsequent perceptual decision stage of the trial. However, when the stimuli finished moving to the centre of the screen and the second, perceptual decision phase of the trial began, the direction of dot motion was rotated by ±90°. Thus, animals could not know until the second stage perceptual decision whether the stimulus would be moving leftwards or rightwards. The unpredictability of the rotation ensured that animals could not make the second stage perceptual decision until after the prospective judgement stage of the trial was over and the subsequent perceptual decision stage of the trial began. Therefore, animals could not predict the motion direction in the perceptual decision stage from that seen in the prior prospective judgement stage. As a result, first-stage prospective decisions and second-stage perceptual decisions occurred at specific and separate times. Again, however, in the second perceptual stages of trials, stimuli were presented continuously until animals completed their choices. If animals judged the motion direction correctly, a juice reward was given according to the ED-probability indicated by the chosen RDK stimulus. On those occasions when the ED-probability meant a reward was to be given, a visual stimulus was shown and a juice reward was delivered. On those occasions when the ED-probability meant no reward was to be given, a different visual stimulus was shown and no juice reward was delivered. When animals misjudged the motion direction, no reward was given irrespective of the probability. After incorrect judgements of motion direction, exactly the same trial was repeated, but the repeated trial was not included in the analyses.

#### Experimental design

Three animals performed the performance-versus-environment task while undergoing fMRI scanning. Each animal contributed 12 sessions (150 correct trials per session) of data (experiment 2). Each session was typically between 50 min and 1 h 40 min. In separate sessions, the same three animals performed the environment-versus-environment task outside the scanner (experiment 1). Each animal contributed 5 sessions (100 correct trials per session) of data. In experiment 3, three animals performed both the performance-versus-environment task and the environment-versus-environment task within the same session. Both tasks were given in a random order on a trial basis within a session to ensure that both types of trial followed TUS with the same range of intervals. Each animal contributed five to seven sessions in which TUS was applied to area 45a, five to seven sessions in which TUS was applied to area 47/12o, five to seven sessions in which TUS was applied to a parietal opercular region (POp)^33^, and five to seven sham TUS sessions. Before the performance of behavioural tasks, animals were given TUS by following the protocol described below in the ‘TUS’ section.

#### Acquisition of MRI data

During the collection of fMRI data, monkeys were head-fixed in a sphinx posture within an MRI-compatible chair (Rogue Research). MR images were acquired with a horizontal bore clinical 3 T scanner with a 15-channel non-human primate-specific receive coil (RAPID Biomedical). For structural images, T1-weighted magnetization-prepared rapid gradient echo images with a resolution of 0.5 × 0.5 × 0.5 mm, repetition time (TR) 2.5 s, echo time (TE) 4.04 ms, inversion pulse time (TI) 1.1 s and flip angle 8°, were acquired in separate sessions under general anaesthesia. Anaesthesia was induced by intramuscular injection of 10 mg kg^−1^ ketamine, 0.125–0.25 mg kg^−1^ xylazine and 0.1 mg kg^−1^ midazolam and maintained with isoflurane (for details, see Sallet et al.^58^). Anaesthesia was used only for collecting T1-weighted structural images. Functional images were acquired via the Center for Magnetic Resonance Research multiband gradient-echo T2* echo planar imaging (EPI) sequences^59,60^. This was characterized by 1.25-mm isotropic voxels with a TR of 1,282 ms, TE of 25.40 ms, multiband acceleration factor MB of 2, in-plane acceleration factor *R* = 2 and flip angle of 63°.

#### TUS

TUS was performed using a four-element annular array transducer (NeuroFUS CTX-250, 42 mm active diameter, Brainbox) combined with a programmable amplifier (Sonic Concept Inc’s Transducer Power Output System, TPO-105, Brainbox). The transducer was paired with a transparent coupling cone filled with degassed water and sealed with a latex membrane. The water was degassed for 4–5 h before each stimulation session and was replaced after each session. The resonance frequency of the ultrasonic wave was set to 250 kHz. The stimulation protocol was based on previously established protocols in macaques^33,61,62^. We used the following protocol: duty cycle 30%; pulse length 30 ms; pulse repetition interval 100 ms; total stimulation duration 40 s. The pressure field from the transducer was measured in a water tank with a 75-µm-diameter polyvinylidene fluoride needle hydrophone (Precision Acoustics) that had been calibrated at 250 kHz by the National Physical Laboratory (Teddington, UK). The free-field spatial-peak pulse-average intensity (intensity spatial peak pulse average, ISPPA) at 41.2 mm focal depth was 80 W cm^−^^2^, which was consistent with the output given by the transducer manufacturer.

At the beginning of each stimulation session, the animal’s skull was shaved and a conductive gel (SignaGel Electrode; Parker Laboratories) was applied to the skin. The water-filled coupling cone and the gel was used to ensure ultrasonic coupling between the transducer and the animal’s head. Next, the ultrasound transducer/coupling cone was placed on the skull, and a Brainsight Neuronavigation System (Rogue Research) was used to position the transducer so that the focal spot would be cantered on the targeted brain region. There were three stimulation conditions: area 45a, area 47/12o and a control condition. The control condition comprised either stimulation of an area unlinked to any aspect of the task in the parietal opercular cortex or sham stimulation (performance did not differ between the two settings). The area 45a and area 47/12o targets were approximately 30 mm, and the POp was 35 mm, from the surface of the transducer (the exact focal distance depended on the subject, and the sham stimulation followed an identical procedure but sonication was not actually triggered). All targets were sonicated bilaterally for 60 s in total, with 30 s of stimulation applied to a target from each hemisphere. Sonication of each area in one hemisphere was immediately followed by sonication of a homologous target in the contralateral hemisphere. Hemispheres were sonicated in a pseudo-random order. After sonication, monkeys were immediately moved to a testing room for behavioural data collection. Each condition was repeated five to seven times, on separate days, and the order of the stimulation sessions was pseudo-randomized for each animal. The stimulation was always performed at the same time of the day, and there was always a 24-h gap between each session, regardless of it being a real or sham stimulation session.

We used the acoustic simulation toolbox (k-Plan, Brainbox) to simulate the depth and acoustic intensity of the TUS focus in area 45a, area 47/12o and area POp (Fig. 6a and Extended Data Fig. 3a). The stimulations used both the T1-weighted scan, and a pseudo-computed tomography scan, which was generated on the basis of the T1-weighted scan. We estimated the pressure, ISPPA and intensity spatial peak time average intensity (ISPTA) at each target region.

Our simulation revealed a pressure of 0.89 MPa, with an ISPPA of 26.4 W cm^−^^2^ and an ISPTA of 7.9 W cm^−^^2^, in the POp left region, and a pressure of 1.17 MPa, with an ISPPA of 46.2 W cm^−^^2^ and an ISPTA of 13.8 W cm^−^^2^, in the POp right region. Similarly, in the left area 45a region, we estimated a pressure of 0.90 MPa, an ISPPA of 27.3 W cm^−^^2^ and an ISPTA of 8.2 W cm^−^^2^, while in the right area 45a, we estimated a pressure of 1.09 MPa, an ISPPA of 40.3 W cm^−^^2^ and an ISPTA of 12.1 W cm^−^^2^. Finally, in the left area 47/12o region, we estimated a pressure of 1.17 MPa, with an ISPPA of 45.7 W cm^−^^2^ and an ISPTA of 13.7 W cm^−^^2^. In the right area 47/12o, we estimated a pressure of 1.13 MPa, with an ISPPA of 43.2 W cm^−^^2^ and an ISPTA of 12.3 W cm^−^^2^.

### Quantification and statistical analysis

#### Behavioural analysis

To evaluate performance during the prospective judgement stage at the beginning of each trial, we used an analysis based on signal detection theory^63^. Specifically, we classified the prospective judgements into trials in which it was optimal for animals to choose the PD-option and trials in which it would be optimal to choose the ED-option. For each participant, if the probability of reward determined by the ED-option was higher than the probability of reward that would be expected given the baseline level of performance of the PD-option (obtained during the perceptual decision stage), then such trials were categorized as ED-task optimal trials. If not, they were categorized as PD-task optimal trials. Based on the proportion of trials in which they chose the PD-task option when the PD-option was optimal (Hit trials) and when the ED-option was optimal (false alarm (FA) trials), we calculated meta-*d*′ (type-II *d*-prime). We calculated meta-*d*′ separately for trials with different coherence levels in the PD-task to control for the difference in baseline perceptual performance and calculated their average (Fig. 1g).

To evaluate the effects on task selection in the initial prospective judgement phase of each trial of reward probability associated with both the PD- and ED-task options, we used logistic multiple regression analyses as shown below:

$$\mathrm{ln}\left(\frac{y(n)}{1-y(n)}\right)=\alpha +{\beta }_{\mathrm{performance}\,}{x}_{\mathrm{performance}}\left(n\right)+{\beta }_{\mathrm{environment}}{x}_{\mathrm{environment}}\left(n\right).$$

Dependent variable *y*(*n*) denotes the task chosen during the prospective judgement stage (PD-task = 1; ED-task = 0) at trial *n*. Independent variables *x*_performance_(*n*) and *x*_environment_(*n*) denote the reward probability of the PD-task and ED-task at trial *n*, respectively. The reward probability here is denoted by the average performance of the PD-task for each motion coherence level during the second perceptual decision stage for each animal (experiment 2: performance-versus-environment task during MRI scanning; experiment 3: performance-versus-environment task during sham TUS sessions) (Figs. 1d and 5d). When the interaction between PD- and ED-probabilities was also included into the multiple regression analysis model, the beta of interaction was not significantly different form zero (*t*_47_ = −0.42, *P* = 0.67) even though the significant positive beta for PD-probability (*t*_47_ = 3.97, *P* = 2.41 × 10^−4^) and the significant negative beta for ED-probability were maintained (*t*_47_ = -6.90, *P* = 1.1 × 10^−8^)

To evaluate the effects of the difference in reward probability between the PD- and ED-task options, we also used logistic multiple regression analyses as shown below:

$$\begin{array}{ll}\mathrm{ln}\left(\frac{y(n)}{1-y(n)}\right) & =\alpha +{\beta }_{\mathrm{performance}-\mathrm{environment}\,}{x}_{\mathrm{performance}-\mathrm{environment}}\left(n\right)\\ & +{\beta }_{\mathrm{performance}+\mathrm{environment}\,}{x}_{\mathrm{performance}+\mathrm{environment}}\left(n\right).\end{array}$$

Independent variable *x*_perfomance-environment_(*n*) denotes the difference in reward probability between the PD-task and ED-task at trial n (Figs. 1e and 6d).

For the evaluation of performance in the environment-versus-environment task, we also used logistic multiple regression analyses as shown below:

$$\mathrm{ln}\left(\frac{y(n)}{1-y(n)}\right)=\alpha +{\beta }_{\mathrm{left}-\mathrm{right}\,}{x}_{\mathrm{left}-\mathrm{right}}\left(n\right).$$

Dependent variable *y*(*n*) denotes the task chosen during the first ED-probability comparison stage (left task = 1; right task = 0) at trial *n*. Independent variable *x*_left-right_(*n*) denotes the difference in ED-probability between the left task and right task at trial *n* (Extended Data Figs. 1b and 5b). We applied these logistic regression analyses to each session data both for experiment 1 and experiment 3.

#### Reconstruction and preprocessing of MRI data

Offline reconstruction of the raw functional data was performed following the dynamic off-resonance correction method developed by Shahdloo et al.^64^. In summary, standard Nyquist ghost correction and dynamic zeroth-order *B*_0_ correction were applied first. Then, the EPI reference navigator data acquired at every timepoint were compared with navigator data from single-band references to estimate first-order dynamic off-resonance perturbations arising from the awake animal’s body movements. Finally, the off-resonance estimates were used to correct the raw data before reconstruction.

Preprocessing of structural and functional MR images was performed using tools from FMRIB’s Software Library (FSL)^57^, MATLAB (R2022a, Mathworks), Advanced Normalization Tools (ANTs; http://stnava.github.io/ANTs)^65^ and Magnetic Resonance Comparative Anatomy Toolbox (MrCat; http://github.com/neuroecology/MrCat)^57^. Iterative preprocessing of magnetization-prepared rapid gradient echo images followed a macaque-optimized pipeline, including brain extraction (using FSL’s brain-extraction tool; BET), RF bias-field correction, and linear and nonlinear registration (using FSL’s FLIRT and FNIRT, respectively) to the *Macaca Mulatta* McLaren F99 template as reported previously^62^.

Although monkeys were head-fixed during MRI acquisition, incidental limb and body movements caused time-varying distortions in the *B*_0_ magnetic field and, therefore, nonlinear motion artefacts along the phase-encoding direction. To account for this, a low-noise EPI volume was identified for each session and then implemented as a reference to which other volumes were linearly and nonlinearly registered slice by slice along the phase-encoding direction. Aligned and distortion-corrected EPIs were then registered nonlinearly first to monkey-specific high-resolution images, and then to a group template in CARET F99 macaque space using ANTs. Further details of the group template construction are described elsewhere^61^. Finally, the functional images were temporally filtered (high-pass temporal filtering, 3-dB cut-off of 100 s) and spatially smoothed (Gaussian spatial smoothing, full width at half maximum of 2.5 mm).

Three measures were used to detect artefacts in the data: (1) for each slice in each volume, the linear transform (in the *y*-plane) from that slice to the corresponding slice in the mean reference image; (2) the normalized correlation between that slice and the corresponding slice in the mean reference image; and (3) for each volume, the correlation between that volume (mean-filtered across *z*-slices) and the mean reference image after correction. Volumes were removed when they exceeded 2.5 s.d. above the median of each measure. The threshold was chosen to keep the number of censored volumes less than 10% of the total volumes. We also added 13 principal component analysis components describing, for each volume, the warping from that volume to the mean reference image when correcting motion artefacts (that is, they capture signal variability associated with motion induced distortion artefacts), as parametric regressors of non-interest that were not convolved in our general linear models (GLMs).

#### Whole-brain fMRI data analysis

We used FSL fMRI Expert Analysis Tool for first-level analysis. First, data was prewhitened with FSL FMRIB’s Improved Linear Model to account for temporal autocorrelations. Temporal derivatives were included into the model. We used an fMRI-GLM to analyse fMRI data across the whole brain. Results were calculated using FSL’s FLAME 1 + 2. To analyse BOLD changes across animals, a second-level analysis was applied across sessions of experiment 2 (FLAME1 + 2).

We included all three phases of a trial (prospective judgement, perceptual decision and outcome) into the fMRI-GLM. Each phase included a constant regressor. The onset of prospective judgement and perceptual decision phases was 1 s before the animals’ touch sensor response with a fixed duration of 1 s. The onset of outcome phase is at time of reward delivery (success) or visual feedback presentation with a duration of 1 s. Parametric regressors were modelled as stick functions (that is, duration of zero) time-locked to the relevant phase onset as below. All parametric regressors were normalized before inclusion into the analysis. In addition, all GLMs contained one regressor time-locked to all button presses, modelled as a stick function, at the first-level fixed-effect analysis stage.

First, we tested for neural correlates of the PD- and ED-task option probabilities during the prospective judgement stage (Figs. 2a and 3a). We included the following regressors, along with the constant regressor coding the phase of prospective judgement in each trial:

Chosen PD-probability, Chosen ED-probability, Unchosen PD-probability, Unchosen ED-probability,

Chosen PD-probability (after incorrect trials), Chosen ED-probability (after incorrect trials), Unchosen PD-probability (after incorrect trials), Unchosen ED-probability (after incorrect trials)

We set these parametric regressors based PD- and ED-probabilities. PD-probability is denoted by the average performance of the PD-task for each motion coherence condition during the second perceptual decision stage for each animal (experiment 2: performance-versus-environment task during MRI scanning; experiment 3: performance-versus-environment task during sham TUS sessions). Then, each regressor was normalized before inclusion into the analysis (mean of 0 and s.d. of 1). If animals chose the PD-task on trial *n*, then the value of the PD-task option and the ED-task option were coded as chosen PD-probability and unchosen ED-probability, respectively. These variables were time-locked to the onset of the prospective judgement stage when animals chose the PD-task. Chosen ED-task probability and unchosen PD-probability were not defined for those trials. If they chose the ED-task on a trial, then the value of the ED-task option and the PD-task option was coded as chosen ED-task probability and unchosen PD-probability, respectively. These variables were time-locked to the onset of the prospective judgement stage when animals chose the ED-task. Chosen PD-probability and unchosen ED-probability were not defined for those trials. A separate set of parametric regressors was applied to the repetition trials that occurred on trials after incorrect decisions. The independent coding of these trials meant that their effects were regressed out from the analyses.

To capture activity related to making decisions about the directions of stimuli, in the perceptual decision stage of each trial, we included the following regressors:

Chosen PD-probability, Chosen ED-probability,

Chosen PD-probability (after incorrect trials), Chosen ED-probability (after incorrect trials)

Then, each regressor was normalized before inclusion into the analysis (mean of 0 and s.d. of 1). These variables were time-locked to the onset of the perceptual decision-making stage.

To capture activity related to the outcome of each decision, the outcome phase included the following regressors:

Outcome of chosen PD-task (1 (correct) or 0 (incorrect)), Outcome of chosen ED-task (1 (rewarded) or 0 (unrewarded))

Then, each regressor was normalized before inclusion into the analysis (mean of 0 and s.d. of 1). These variables were time-locked to the onset of the outcome stage. The outcome variable was defined for the task chosen: for example, if participants chose the PD-task, then the outcome of the chosen ED-task was not defined.

#### Region of interest fMRI analyses

We used regions of interest (ROIs) with a radius of three voxels (size = 33 voxels) that were centred, separately, on area 45a and area 47/12o. Each ROI comprised a cube centred on two adjacent voxels on either side of the central coordinate together with the central voxel itself. It comprised a cube with a side of 3 voxels (3^3^ = 27 voxels) and 6 additional voxels located outwards from the centre of each face of the cube. In total, signal from 33 voxels was used for each ROI. The selected ROI was transformed from Montreal Neurological Institute space to subject space, and the preprocessed BOLD time courses were extracted for each session. Time courses were averaged across volumes, then normalized and oversampled by a factor of 20 for visualization. ROI-GLM were applied to each timepoint to derive beta weights per timepoint for each regressor. For analyses across conditions, we used the same principle as applied to the whole-brain fMRI-GLM: we averaged across the group. For all ROI analyses, regressors were normalized (mean of 0 and s.d. of 1). We used the same parametric predictors described in the whole-brain fMRI analysis conducted with fMRI-GLM for the prospective judgement stage, perceptual decision stage and outcome stage. We also time-locked the time courses to the same phase onsets as described in the ‘Whole-brain fMRI data analysis’ section.

#### Resting-state fMRI data preprocessing and analysis

Resting-state (rs-)fMRI and anatomical scans were collected for eight healthy macaques (*M. mulatta*) under light inhalational anaesthesia with isoflurane (for detailed information on anaesthesia protocol, monitoring of vital signs, data acquisition and preprocessing; see Neubert et al.^6^). We drew cubic ROIs (3-voxels isotropic) centred on the peak of task-related activations identified by the present study (right area 45a and area 47/12o) and eight task-related areas listed in Extended Data Table 1 and illustrated in Fig. 7c and 23 task-independent peaks used by Neubert et al.^6^ (Fig. 7b). These ROIs were registered from Montreal Neurological Institute space to each subject’s individual rs-fMRI space via the T1-weighted structural image using FNIRT and brain boundary-based registration. Then, the major Eigen time series representing activity in each of the ROIs was calculated. Individual statistical maps were then calculated using a seed-based correlation analysis, which is part of FSL (fsl_sbca), as previously described^6,66^, to infer the functional connectivity of these ROIs with the rest of the brain. We also included nuisance regressors encoding, separately, the time series of signals from white matter and cerebrospinal fluid. Unthresholded group *z*-maps were quantified by extracting the average intensity of each ROI’s functional connectivity *z*-map. As in the macaques, the same pipeline was applied to human data from HCP. Human ROIs were defined by task-related activation peak including left alPFC in the work of Miyamoto et al.^5^ and task-independent peaks used by Neubert et al.^6^.

It is noteworthy that each macaque ROI (the ‘target’ areas in the fc analysis) had a corresponding human ROI, which was determined a priori by interspecies similarity in their task-based activity (Fig. 7c) or interspecies similarity in their rs-fMRI fc patterns with yet other ROIs distributed across the whole brain^6^ (Fig. 7b). We examind the fc patterns of three ‘seed’ regions, in area 45a and in area 47/12o in the macaque, alPFC in humans, with the target areas elsewhere in the brain. A formal comparison between human and macaque coupling patterns was performed by calculating the summed absolute difference (the Manhattan distance^6,38,58^ of the coupling scores) between human alPFC’s connectivity estimates with the other human ROIs and the connectivity estimates of macaque 45a or 47/12o with the corresponding macaque ROIs. The analysis was performed once using areas identified by Neubert et al.^6^ that were identified independently of task-performance (Fig. 7b) and once using task-related areas (Fig. 7c). Before calculating the Manhattan distance between two independent fc patterns, fc values were standarized within each network by mean = 0 and s.d. = 1. It is noted that our main observations were reproduced when maximum and minimum fc values were normalized as 1 and 0, respectively, as in previous studies^6,38,58^. Statistical significance of interspecies similarity between the macaque fc network inclusively formed by fc networks with area 45a and area 47/12o (Fig. 7c, yellow trace) and human fc network with alPFC (Fig. 7c, orange trace), was assessed by permutation testing. To make the combined fc network, we took the larger fc value from either the seed placed at area 45a or at 47/12o for each target area. We shuffled the pairs between human and macaque ROIs and calculated the Manhattan distance based on the randomly generated networks 1,000 times. We tested whether the observed Manhattan distance was significantly smaller than the distance obtained under a null hypothesis based on the frequency distribution of Manhattan distances in a randomly interconnected network (Fig. 7c).

#### Statistics

We corrected *P* values for multiple comparisons when necessary. The statistical tests used were two-sided. For identification of the areas predicting prospective judgement, we applied FWE correction across the whole-brain volume. Error bars in the figures depict standard errors of the mean (s.e.m.).

### Reporting summary

Further information on research design is available in the Nature Portfolio Reporting Summary linked to this article.

## Supplementary information

Reporting Summary

Peer Review File

## Figures and Tables

**Extended Data Table 1:**
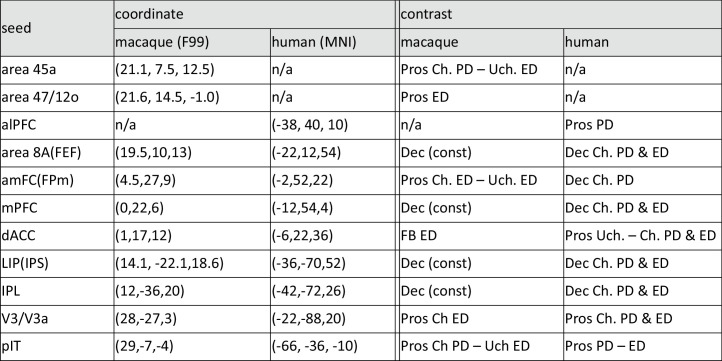
Eight key regions along with macaque areas 45a and 47/12o and human alPFC, which were determined based on the brain activity during the current task

fMRI activity at the coordinates in the table were used as the seed of functional connectivity (FC) analysis for inter-species FC-patterns comparison. *Dec* indicates that the contrast pertains to second stage, perceptual decisions, *Pros* indicates that a contrast pertains to the first part of the trial – the prospective judgement stage and FB indicates that the contrast pertains to outcome feedback stage. Ch.: chosen, Uch: unchosen, PD: Performance-dependent option, ED: Environment-dependent option.

## Data Availability

Data that support the findings of this study are available via RIKEN CBS Data Sharing Platform at https://neurodata.riken.jp/ with an identifier (10.60178/cbs.20260313-001).

## Code availability

Codes that support the findings of this study are available via RIKEN CBS Data Sharing Platform at https://neurodata.riken.jp/ with an identifier (10.60178/cbs.20260313-001) and via GitHub at http://github.com/neuroecology/MrCat.
