# Supplementary material for: Brain activity, disruption and connectivity comparisons identify origins of human metacognition in other primates
Source: Nat Hum Behav. 2026 May 18;10(8):1557–78. doi: 10.1038/s41562-026-02473-w (PMC13485697; doi:10.1038/s41562-026-02473-w)
Supplement: Supplementary file 1 [file 41562_2026_2473_MOESM1_ESM.pdf]

nature portfolio

Corresponding author(s): Kentaro Miyamoto

Last updated by author(s): Mar 30, 2026

Reporting Summary

Nature Portfolio wishes to improve the reproducibility of the work that we publish. This form provides structure for consistency and transparency in reporting. For further information on Nature Portfolio policies, see our Editorial Policies and the Editorial Policy Checklist.

Statistics

For all statistical analyses, confirm that the following items are present in the figure legend, table legend, main text, or Methods section.

n/a	Confirmed	
☐	☒	The exact sample size (n) for each experimental group/condition, given as a discrete number and unit of measurement
☐	☒	A statement on whether measurements were taken from distinct samples or whether the same sample was measured repeatedly
☐	☒	The statistical test(s) used AND whether they are one- or two-sided<br>*Only common tests should be described solely by name; describe more complex techniques in the Methods section.*
☐	☒	A description of all covariates tested
☐	☒	A description of any assumptions or corrections, such as tests of normality and adjustment for multiple comparisons
☐	☒	A full description of the statistical parameters including central tendency (e.g. means) or other basic estimates (e.g. regression coefficient) AND variation (e.g. standard deviation) or associated estimates of uncertainty (e.g. confidence intervals)
☐	☒	For null hypothesis testing, the test statistic (e.g. F, t, r) with confidence intervals, effect sizes, degrees of freedom and P value noted<br>*Give P values as exact values whenever suitable.*
☒	☐	For Bayesian analysis, information on the choice of priors and Markov chain Monte Carlo settings
☒	☐	For hierarchical and complex designs, identification of the appropriate level for tests and full reporting of outcomes
☐	☒	Estimates of effect sizes (e.g. Cohen's d, Pearson's r), indicating how they were calculated

Our web collection on statistics for biologists contains articles on many of the points above.

Software and code

Policy information about availability of computer code

Data collection	We used custom codes running with Presentation software (Neurobehavioral systems) for behavioural experiments.
Data analysis	We used custom codes for analyses of behavioural and fMRI data running with MATLAB R2022a (MathWorks) and FSL (v.6.0; FMRIB, Oxford).

For manuscripts utilizing custom algorithms or software that are central to the research but not yet described in published literature, software must be made available to editors and reviewers. We strongly encourage code deposition in a community repository (e.g. GitHub). See the Nature Portfolio guidelines for submitting code & software for further information.

Data

Policy information about availability of data

All manuscripts must include a data availability statement. This statement should provide the following information, where applicable:

- Accession codes, unique identifiers, or web links for publicly available datasets
- A description of any restrictions on data availability
- For clinical datasets or third party data, please ensure that the statement adheres to our policy

The data and code that support the findings of this study is shared online at https://neurodata.riken.jp/

Research involving human participants, their data, or biological material

Policy information about studies with human participants or human data. See also policy information about sex, gender (identity/presentation), and sexual orientation and race, ethnicity and racism.

Reporting on sex and gender	We reported only the sex (biological attribute) of participants throughout the manuscript. We confirmed that our manuscript did not use either the term sex or gender explicitly in the manuscript.
Reporting on race, ethnicity, or other socially relevant groupings	We did not use any socially constructed or socially relevant categorization variables in our manuscript.
Population characteristics	Human behaviour and fMRI data acquired during the performance of similar tasks (fMRI experiment: N=23; 2 sessions for each participant.TMS experiment: N=8; 2 blocks for each stimulation conditions [alPFC or vertex] in each participant) (Miyamoto et al., 2021) are also re-analysed and presented. Final sample of fMRI experiment: 15 female and 8 male; age (mean ± SD), 28.2 ± 6.7. Final sample of TMS experiment: 5 female and 3 male; age (mean ± SD), 25.8 ± 4.4.
Recruitment	Participants were recruited via Department of Experimental Psychology Oxford Psychology Research (OPR) participant recruitment scheme. There is not any potential biases that are likely to impact results.
Ethics oversight	The study was approved by the Central Research Ethics Committee (fMRI experiment: MSD-IDREC-R51506/RE002, TMS experiment: R65502/RE001) at the University of Oxford. All participants gave informed consent.

Note that full information on the approval of the study protocol must also be provided in the manuscript.

Field-specific reporting

Please select the one below that is the best fit for your research. If you are not sure, read the appropriate sections before making your selection.

☒ Life sciences ☐ Behavioural & social sciences ☐ Ecological, evolutionary & environmental sciences

For a reference copy of the document with all sections, see nature.com/documents/nr-reporting-summary-flat.pdf

Life sciences study design

All studies must disclose on these points even when the disclosure is negative.

Sample size	We did not perform sample-size calculation but we chose the similar sample size as those used in previous studies by Behaviour (Experiment 1: N=3x5 sessions. Experiment 2: N=4 x 12 sessions), fMRI (Experiment 2: N=3 x 12 sessions; final 35 sessions) and TUS (Experiment 3: N=3 x 5-7 sessions for each TUS condition; final, 45 disruption: 20 sessions, 47/12o disruption: 21 sessions, POp disruption: 19 sessions, sham control: 18 sessions).
Data exclusions	For Experiment 2, one session was excluded because of failures in reconstruction of functional images.
Replication	Behaviour of fMRI and bahaviour-only experiments (Experiment 1 and 2) were reproduced in the baseline sham condition of TUS experiment (Experiment 3).
Randomization	In TUS experiment (Experiment 3), the order of the stimulation sessions was pseudo-randomized for each animal. All targets were sonicated bilaterally for 60s in total, with 30s of stimulation applied to a target from each hemisphere. Sonication of each area in one hemisphere was immediately followed by sonication of a homologous target in the contralateral hemisphere. Hemispheres were sonicated in a pseudo-random order.
Blinding	Animals and experimenters who collected TUS data in Experiment 3 did not know the details of disruption sites except for the location.

Reporting for specific materials, systems and methods

We require information from authors about some types of materials, experimental systems and methods used in many studies. Here, indicate whether each material, system or method listed is relevant to your study. If you are not sure if a list item applies to your research, read the appropriate section before selecting a response.

Materials & experimental systems

n/a	Involved in the study
☒	☐ Antibodies
☒	☐ Eukaryotic cell lines
☒	☐ Palaeontology and archaeology
☐	☒ Animals and other organisms
☒	☐ Clinical data
☒	☐ Dual use research of concern
☒	☐ Plants

Methods

n/a	Involved in the study
☒	☐ ChIP-seq
☒	☐ Flow cytometry
☐	☒ MRI-based neuroimaging

Animals and other research organisms

Policy information about studies involving animals; ARRIVE guidelines recommended for reporting animal research, and Sex and Gender in Research

Laboratory animals	Adult monkeys (Macaca mulatta) aged between 7-10 years and weighed between 11-17 kg at the time of the experiments.
Wild animals	The study did not involve wild animals.
Reporting on sex	Only male animals were used for the study. Since social housing without breeding is mandatory, only males are maintained in the facility.
Field-collected samples	The study did not involve samples collected from the field.
Ethics oversight	All experiments were conducted in accordance with European Union Directive 2010/63/EU of the European Parliament on the protection of animals used for scientific purposes and with the United Kingdom (UK) Animals (Scientific Procedures) Act 1986 under licenses approved by the UK Home Office.

Note that full information on the approval of the study protocol must also be provided in the manuscript.

Plants

Seed stocks	*Report on the source of all seed stocks or other plant material used. If applicable, state the seed stock centre and catalogue number. If plant specimens were collected from the field, describe the collection location, date and sampling procedures.*
Novel plant genotypes	*Describe the methods by which all novel plant genotypes were produced. This includes those generated by transgenic approaches, gene editing, chemical/radiation-based mutagenesis and hybridization. For transgenic lines, describe the transformation method, the number of independent lines analyzed and the generation upon which experiments were performed. For gene-edited lines, describe the editor used, the endogenous sequence targeted for editing, the targeting guide RNA sequence (if applicable) and how the editor was applied.*
Authentication	*Describe any authentication procedures for each seed stock used or novel genotype generated. Describe any experiments used to assess the effect of a mutation and, where applicable, how potential secondary effects (e.g. second site T-DNA insertions, mosiacism, off-target gene editing) were examined.*

Magnetic resonance imaging

Experimental design

Design type	task (prospective judgement task); event-related design
Design specifications	150 correct trials per session; 12 sessions per animal; ~12sec/trial; 1 sec/ITI
Behavioral performance measures	Beta coefficients of the PD- and ED-probability in logistic multiple regression analysis to predict choices of the PD-option. Signal detection theory-based sensitivity indices to summarize whether, when making the first judgement, the monkeys chose the optimal option for the second task stage.

Acquisition

Imaging type(s)	functional, Structural
Field strength	3T
Sequence & imaging parameters	Functional images were acquired via the CMMR multiband gradient-echo T2* echo planar imagFor structural images, T1-weighted MP-RAGE images with a resolution of 0.5 × 0.5 × 0.5 mm, TR 2.5 s, TE 4.04 ms, inversion pulse time (TI) 1.1 s, and flip angle 8°, were acquired in separate sessions under general anaesthesiaing (EPI) sequences. This was

characterised by 1.25mm isotropic voxels with a repetition time (TR) of 1282ms, echo time (TE) of 25.40ms, multiband acceleration factor MB=2, in-plane acceleration factor R=2, and flip angle of 63°. For structural images, T1-weighted MP-RAGE images with a resolution of 0.5 × 0.5 × 0.5 mm, TR 2.5 s, TE 4.04 ms, inversion pulse time (TI) 1.1 s, and flip angle 8°, were acquired in separate sessions under general anaesthesia.

Area of acquisition: Any volume censoring was not applied.

Diffusion MRI ☐ Used ☒ Not used

Preprocessing

Preprocessing software: Pre-processing of structural and functional MR images was performed using tools from FMRIB's Software Library (FSL), MATLAB (R2022a, Mathworks), Advanced Normalization Tools (ANTs; http://stnava.github.io/ANTs) and Magnetic Resonance Comparative Anatomy Toolbox (MrCat; http://github.com/neuroecology/MrCat).

Normalization: Iterative pre-processing of MP-RAGE images followed a macaque-optimised pipeline, including brain-extraction (using FSL's brain-extraction tool; BET), RF bias-field correction, and linear and non-linear registration (using FSL's FLIRT and FNIRT, respectively)

Normalization template: Macaca Mulatta McLaren F99 template

Noise and artifact removal: Offline reconstruction of the raw functional data was performed following the dynamic off-resonance correction method developed by Shahdloo et al. In summary, standard Nyquist ghost correction and dynamic zeroth-order B0 correction were applied first. Then, the EPI reference navigator data acquired at every time-point was compared to navigator data from single-band references to estimate first-order dynamic off-resonance perturbations arising from the awake animal's body movements. Finally, the off-resonance estimates were used to correct the raw data prior to reconstruction. A low-noise EPI volume was identified for each session and then implemented as a reference to which other volumes were linearly and non-linearly registered slice-by-slice along the phase-encode direction.

Volume censoring: Any volume censoring was not applied.

Statistical modeling & inference

Model type and settings: univariate design; first level, fixed effect; second level, mixed effect (FLAME 1+2)

Effect(s) tested: Parametric effects of Chosen performance-dependent(PD)-probability, Unchosen PD-probability, Chosen environment-dependent(ED)-probability, Unchosen ED-probability at the onsets of prospective judgement were tested. Neither ANOVA nor factorial design was used.

Specify type of analysis: ☒ Whole brain ☐ ROI-based ☐ Both

Statistic type for inference: cluster-wise (z > 3.1), voxel-wise

(See Eklund et al. 2016)

Correction: FWE

Models & analysis

n/a	Involved in the study
☐	☒ Functional and/or effective connectivity
☒	☐ Graph analysis
☒	☐ Multivariate modeling or predictive analysis

Functional and/or effective connectivity: Pearson correlation
