# Supplementary material for: Brain activity, disruption and connectivity comparisons identify origins of human metacognition in other primates
Source: Nat Hum Behav. 2026 May 18;10(8):1557–78. doi: 10.1038/s41562-026-02473-w (PMC13485697; doi:10.1038/s41562-026-02473-w)
Supplement: Supplementary file 2 [file 41562_2026_2473_MOESM2_ESM.pdf]

nature portfolio

Peer Review File

Brain activity, disruption, and connectivity comparisons identify origins of human metacognition in other primates

Corresponding Author: Dr Kentaro Miyamoto

Version 0:

Decision Letter:

24th September 2025

Dear Dr Miyamoto,

Thank you once again for your manuscript, entitled "Neural recording, disruption, and connectivity comparisons identify origins of human metacognition in other primates", and for your patience during the peer review process.

Your Article has now been evaluated by 3 referees. You will see from their comments copied below that, although they find your work of potential interest, they have raised quite substantial concerns. In light of these comments, we cannot accept the manuscript for publication, but would be interested in considering a revised version if you are willing and able to fully address reviewer and editorial concerns.

We hope you will find the referees' comments useful as you decide how to proceed. If you wish to submit a substantially revised manuscript, please bear in mind that we will be reluctant to approach the referees again in the absence of major revisions. We are committed to providing a fair and constructive peer-review process. Do not hesitate to contact us if there are specific requests from the reviewers that you believe are technically impossible or unlikely to yield a meaningful outcome.

In particular, Reviewer #1 has substantial concerns about the strength of the evidence and robustness of the results. In the revised manuscript, we request a fully transparent presentation of the raw data to allow for a more detailed evaluation.

Finally, your revised manuscript must comply fully with our editorial policies and formatting requirements. Failure to do so will result in your manuscript being returned to you, which will delay its consideration. To assist you in this process, I have attached a checklist that lists all of our requirements. If you have any questions about any of our policies or formatting, please don't hesitate to contact me.

If you wish to submit a suitably revised manuscript, we would hope to receive it within 4 months. I would be grateful if you could contact us as soon as possible if you foresee difficulties with meeting this target resubmission date.

With your revision, please:

• Include a “Response to the editors and reviewers” document detailing, point-by-point, how you addressed each editor and referee comment. If no action was taken to address a point, you must provide a compelling argument. When formatting this document, please respond to each reviewer comment individually, including the full text of the reviewer comment verbatim followed by your response to the individual point. This response will be used by the editors to evaluate your revision and sent back to the reviewers along with the revised manuscript.

• Highlight all changes made to your manuscript or provide us with a version that tracks changes.

Please use the link below to submit your revised manuscript and related files:

Link Redacted

Note: This URL links to your confidential home page and associated information about manuscripts you may have submitted, or that you are reviewing for us. If you wish to forward this email to co-authors, please delete the link to your homepage.

Thank you for the opportunity to review your work. Please do not hesitate to contact me if you have any questions or would like to discuss the required revisions further.

Sincerely,

[redacted]
Nature Human Behaviour

Reviewer expertise:

Reviewer #1: Comparative cognition in monkeys and humans, fMRI, TMS

Reviewer #2: Decision making, non-human primates, computational neuroscience

Reviewer #3: Metacognition, subjective experience, computational neuroscience

REVIEWER COMMENTS:

Reviewer #1 (Remarks to the Author):

The authors propose a very smooth attractive story about rudiments of metacognition (decisions in the future) in the macaque, but the experimental evidence they provide raises many doubts.
1) the fMRI signals are very weak, especially in area 45: see fig 2 and fig 3A,B. the contrast with the standard in the literature eg the papers of D Tsao or W Freiwald is stark. fMRI signals are weak despite the averaging over 4 monkeys. The main text does not mention any single subject data: given the weakness of signals, we need to be reassured that the activations are present in each animal. Nor do the S figures provide any single subject data. But they provide plenty of small wavy signals. The data for area 47/12° are slightly stronger, but the differences in fig 4C between chosen and unchosen ED are very noisy. Again single subject data are needed. One possible problem is that the authors attempted to cramp 3 data sets into a single manuscript for a large audience journal of which most readers are unfamiliar with the pitfalls of fMRI, making it difficult to do justice to the data; S figures are intended to help this.
2) Also it is unclear where exactly the activation is localized: we are given a single coronal section, without any indication of areal borders. 3D brain volumes are very poor to precisely localize activations, flattened cortex with areal borders indicated would do a much better job. This the more pressing that in fig 2a the activation ascribed to area 45 seems to shift from the surface to the depth of sulcus between the chosen and unchosen PD conditions. This is intriguing given that the Lupino group has described two areas 45: 45A and 45B. The latter is in the anterior bank of inferior arcuate sulcus,next to FEF, while 45A is on the surface in front of 45B. Given the difference in anatomical connections (Gerbella et al Cerbr Cortex 2010), these two parts are considered separate areas; Also 45B, and not 45A is more visually responsive (Nelissen et al science 2005). Given the absence of any areal limit indication we are completely in the unknown about which area is activated, if any. A further problem is that the activation is described as area 45,implying bilateral activations, but the activations seems even weaker on the left side. Again we have no single subject data. We need evidence that the location of the activation, if present, is consistent in the 4 monkeys and indeed corresponds to area 45 or one of its parts. same for area 47/12°.
3) In general the localization of activations remains an issue by the reliance of 3D brain volumes or surface views: as an example in fig S3 the location of FEF is unclear.
4) What is the evidence that the TUS impaired the relevant area? The only thing we are provided with (in fig S5) is a simulation. The value of such simulation is weak. At least the authors could have manipulated the focal distance to show that the behavioral effect was maximal when the focal distance matched the location of the target area. It seems that the TUS was delivered bilaterally, how this fit with the activation which is asymmetric (fig 2)
5) The functional connectivity part is extremely weak as the underlying mechanisms of functional connectivity are very diverse, ranging from direct anatomical connections to multi-synaptic connections over 1, 2,… n stages; in addition these intervening stages are not guaranteed to be identical in different species. Hence it is very difficult to derive anything from a similarity in functional connectivity between species as done in fig 7C. A further problem is that this figure assumes homologies between human and macaque areas, for which no evidence is provided. Often this group has used functional connectivity to derive homologies between macaque and human areas, which is then pretty close to circular reasoning. In addition establishment of homologies in the parieto-occipital cortex is particularly hard, given the extension of the intraparietal sulcus into occipital cortex in humans.
6) in the discussion the authors interpret their data to mean that the two areas 45 and 47° of the macaque fuse into a single medial prefrontal area in humans; This is an extremely unlikely scenario; Given the 10 fold increase in cortical surface in humans compared to macaque, it is generally assumed that cortical areas of the macaque split into two and yield two human areas, not the reverse. A more likely scenario (if the data are proven to be correct) would be that area 47/12° of the macaque gives raise the medial prefrontal area of humans, and that the selectivity of macaque 45 to PD signals moved to this human area, because the homologue of 45 (or its parts) is colonized by novel afferents, probably those related to semantic or linguistic processing. Such a scenario would fit the large expansion of cortex in medial prefrontal cortex and the presence of a uniquely human area dACC (based on functional connections but combined with functional responses!) in this region (fig 10, Mantini et al J Neurosci 2013)

A minor point is the lack of clarity of the description of the two behavioral tasks; it took me a long time to understand them and fig 1 did not help, to the contrary.

Guy Orban

Reviewer #2 (Remarks to the Author):

Miyamoto K et al., Neural recording, disruption, and connectivity comparisons identify origins of human metacognition in other primates

The authors describe a study in which they used fMRI to examined prospective decision confidence judgements in macaques and humans. In the task, subjects were shown two stimuli, one in which there were many dots, but the coherence level varied from trial-to-trial (the PD stimulus) and the other in which the number of dots varied (which signaled the probability of receiving a reward for a correct choice) but the coherence level was 1, such that the perceptual inference was easy. The subjects had to choose one of these tasks at the first stage, after which they were given the chosen decision-making task at the second stage. Monkeys and humans showed that they could predict future performance levels at the first stage, and made choices that took into account both their PD performance, and the ED level. The monkeys were not as good at predicting performance for difficult PD trials. Analysis of the imaging data showed that macaque area 45 was related to the PD option, and activity peaked earlier on trials in which the PD option was chosen. Area 47/12o, on the other hand, was related to the ED option. There were no latency differences in area 47 in chosen vs. unchosen ED trials.

In general, this study addresses important questions about not only homology across macaque and human prefrontal cortical areas, but also decision processes within primate prefrontal cortex related to metacognition. I found the paper to be very well written and straightforward. The use of TUS to disrupt processing within each area also substantially increased the impact of the work. I have several questions that should be clarified, however.

Comments

1. For the analysis in Figure 1g, it also seems likely that the animals may be assessing their willingness to exert effort in the decision stage. If they are willing to exert more effort, they will likely do better. On the contrary, it is easy to simply make an easy inference in the decision stage, and then accept the ED outcome. This should at least be discussed.

2. Could eye movements account for latency differences in area 45? If the animals look at the PD option earlier in the trial, or spend more time looking at the PD option when it is ultimately chosen, this would likely drive correlations with the coherence level higher faster. This has been shown for other decision processes in OFC.

3. “First, as demonstrated in Fig. S4a, the signal in area 45 was modulated by the PD-probability in a different way when that option was chosen and when the ED-option was chosen even though, on average, the PD- and ED-options have the same reward probabilities.” However, they do not have the same subjective reward probabilities. I don’t think it’s necessary to redo all analyses with subjective reward probabilities, but somehow this statement should be qualified?

4. “For the TUS experiments we returned to using the two versions of the task in which animals either chose between ED-options…” The ED/ED task was explained in the figure legend, but not in the main text. Some mention of it could be given in the main text of the results. Otherwise it comes out of the blue in this sentence, much later in the results.

5. As the authors know, it is normally assumed that as brains expand, a single area gets larger before it splits into two sub-areas that then can take on disparate functions. The current results are inconsistent with this standard model. It would be worth at least discussing this normal account of homology in the context of the current results.

6. “further” is repeated twice in this sentence: “To further examine judgements in the first stages of trials further we carried out logistic multiple regression analyses…”

Reviewer #3 (Remarks to the Author):

I have reviewed this paper twice before for another journal. The authors have now largely reworked the paper to address previous concerns raised during those rounds of detailed review, and I think the current version of the paper will make a strong contribution to the literature. This version has been somewhat reworked to highlight the contrast between the macaque and human findings on the same task, which is also a strength.

My one remaining recommendation when reading the current version is that some statistical comparisons are needed between macaques and humans if these claims are to be robustly substantiated. Eg. On p.4, the differences in PD-related choices between species should be supported with a test of the condition x species interaction. And again at the top of p.5 – it’s not enough to say that the PD beta was smaller than the ED for macaques but not in humans – we need a formal test of the group x condition interaction.

Version 1:

Decision Letter:

Our ref: NATHUMBEHAV-25084077A

5th March 2026

Dear Dr Miyamoto,

Thank you for submitting your revised manuscript "Neural recording, disruption, and connectivity comparisons identify origins of human metacognition in other primates" (NATHUMBEHAV-25084077A). It has now been seen by the original referees and their comments are below. As you can see, the reviewers find that the paper has improved in revision. We will therefore be happy in principle to publish it in Nature Human Behaviour, pending revisions to satisfy the referees' final requests and to comply with our editorial and formatting guidelines.

We are now performing detailed checks on your paper and will send you a checklist detailing our editorial and formatting requirements within two weeks. Please do not upload the final materials and make any revisions until you receive this additional information from us.

Please do not hesitate to contact me if you have any questions.

Sincerely,

[redacted]
Nature Human Behaviour

Reviewer #1 (Remarks to the Author):

The authors performed pioneering behavioral studies in metacognition in macaques and combined this with fMRI, for comparison with similar data in humans. This is highly commendable. In addition in their revision they performed several control analyses and a control experiment with TUS, which strengthen the results of the first two parts of the manuscript.
In part 1, the authors should be more careful in describing the fMRI signal; This is an hemodynamic signal that only indirectly reflects neural activity (which is electrical); Many readers of Nat Hum Beh may not be aware of this distinction and will be misled by the expression ‘neural activity' which is used in the main text and also the title. It is strongly recommended to use hemodynamic signal or perhaps brain activity but not neural activity.
The measure of hemodynamic vs electrical signals has another important consequence: given the long time constant of the hemodynamic filter: a time course difference in fMRI signal, as reported here, is hard to interpret: an earlier latency of the fMRI signal can indeed reflect an earlier neuronal response (as implied by the authors) but equally well a steeper rising neuronal response. It is important to point out this ambiguity in the discussion.
In part 2, the TUS control experiment (much appreciated) indeed indicates that TUS has the appropriate spatial resolution. But this does not really answer my initial comment. How do we know that the TUS effect is due to the blocking of 45a and not one of the other areas also affected by the TUS (see the cone in figure 6).
While parts 1 and 2 are now acceptable given appropriate rewriting, part 3 remains below the standards and should be removed;
Both in the reply to my comments as in the revised text, the authors claim the correlations across studies between fc and some type of anatomical connection indicate the value of this technique; this highly disputable as for given fc one cannot know which is the underlying anatomy: it can be a monosynaptic connection, but equally well a di- or multi-synaptic connection. In the latter cases even if the number of intervening synapses is equal, they can very well be located in different anatomical structures. Hence the comparison of spider plots as used all the time in part 3 is impossible: equal values do NOT mean the same anatomical connection. In addition differences between ‘spider' plots cannot be assessed as no variability is provided. Furthermore when comparing spider plots between species more problems arise as it has to be assumed the brain regions used in the spider plots are homologous. This is a difficult enterprise and for very few cortical areas, outside primary areas, homology between macaque and human have been proven, MT and its cluster being a nice exception. The authors are aware of the problem and stated that the areas in the spider plots they compare show similar functional activity in their fMRI data. This is insufficient as multiple converging evidence is needed to demonstrate homology. Given the size and the complexity of the manuscript, dropping the third weak part would free space to point out the limitations of the data described in the valuable first and second parts.
Minor comments:
1) Authors should diversify the references rather than auto-quote; In particular an appropriate reference for the division of

area 45 into a and b is needed: Petrides & Pandya (2002) only addressed area 45 in general.
2) In the discussion, migration of afferents to a different cortical area, is NOT the same as fusion of two areas; the authors may have to accept this point explicitly.

Reviewer #2 (Remarks to the Author):

The authors have carefully addressed my concerns. I have no further comments.

Reviewer #3 (Remarks to the Author):

The authors have addressed all my remaining concerns.

Open Access This Peer Review File is licensed under a Creative Commons Attribution 4.0 International License, which permits use, sharing, adaptation, distribution and reproduction in any medium or format, as long as you give appropriate credit to the original author(s) and the source, provide a link to the Creative Commons license, and indicate if changes were made.
In cases where reviewers are anonymous, credit should be given to 'Anonymous Referee' and the source.
The images or other third party material in this Peer Review File are included in the article's Creative Commons license, unless indicated otherwise in a credit line to the material. If material is not included in the article's Creative Commons license and your intended use is not permitted by statutory regulation or exceeds the permitted use, you will need to obtain permission directly from the copyright holder.
To view a copy of this license, visit https://creativecommons.org/licenses/by/4.0/

<u>Response to the editors and reviewers</u>

Reviewer #1 (Remarks to the Author):

The authors propose a very smooth attractive story about rudiments of metacognition (decisions in the future) in the macaque, but the experimental evidence they provide raises many doubts.

1) the fMRI signals are very weak, especially in area 45: see fig 2 and fig 3A,B. the contrast with the standard in the literature eg the papers of D Tsao or W Freiwald is stark. fMRI signals are weak despite the averaging over 4 monkeys. The main text does not mention any single subject data: given the weakness of signals, we need to be reassured that the activations are present in each animal. Nor do the S figures provide any single subject data. But they provide plenty of small wavy signals. The data for area 47/12° are slightly stronger, but the differences in fig 4C between chosen and unchosen ED are very noisy. Again single subject data are needed. One possible problem is that the authors attempted to cramp 3 data sets into a single manuscript for a large audience journal of which most readers are unfamiliar with the pitfalls of fMRI, making it difficult to do justice to the data; S figures are intended to help this.

Response:
Thank you for pointing out that inter-subject consistency of the results is important for assessing the reliability of fMRI signals of macaques. By following the reviewer's suggestions, we have newly provided single subject activation maps of each three monkeys (three out of four participated in fMRI scanning experiments) in a similar manner as Fig 1a of Tsao, Schweers, Moeller, Freiwald (2008, Nat. Neurosci.). As shown in the new Fig. S4a, the group activity at area 45a (see also our response to the reviewer's main point 2 below) in response to chosen PD-probability against unchosen ED-probability (Fig. 2a) is consistent across three animals when analysed separately. On the other hand, as shown in the new Fig. S4b, the group activity at area 47/12o in response to both of the chosen and unchosen ED-probabilities (Fig. 4a) is also consistent across the three animals.

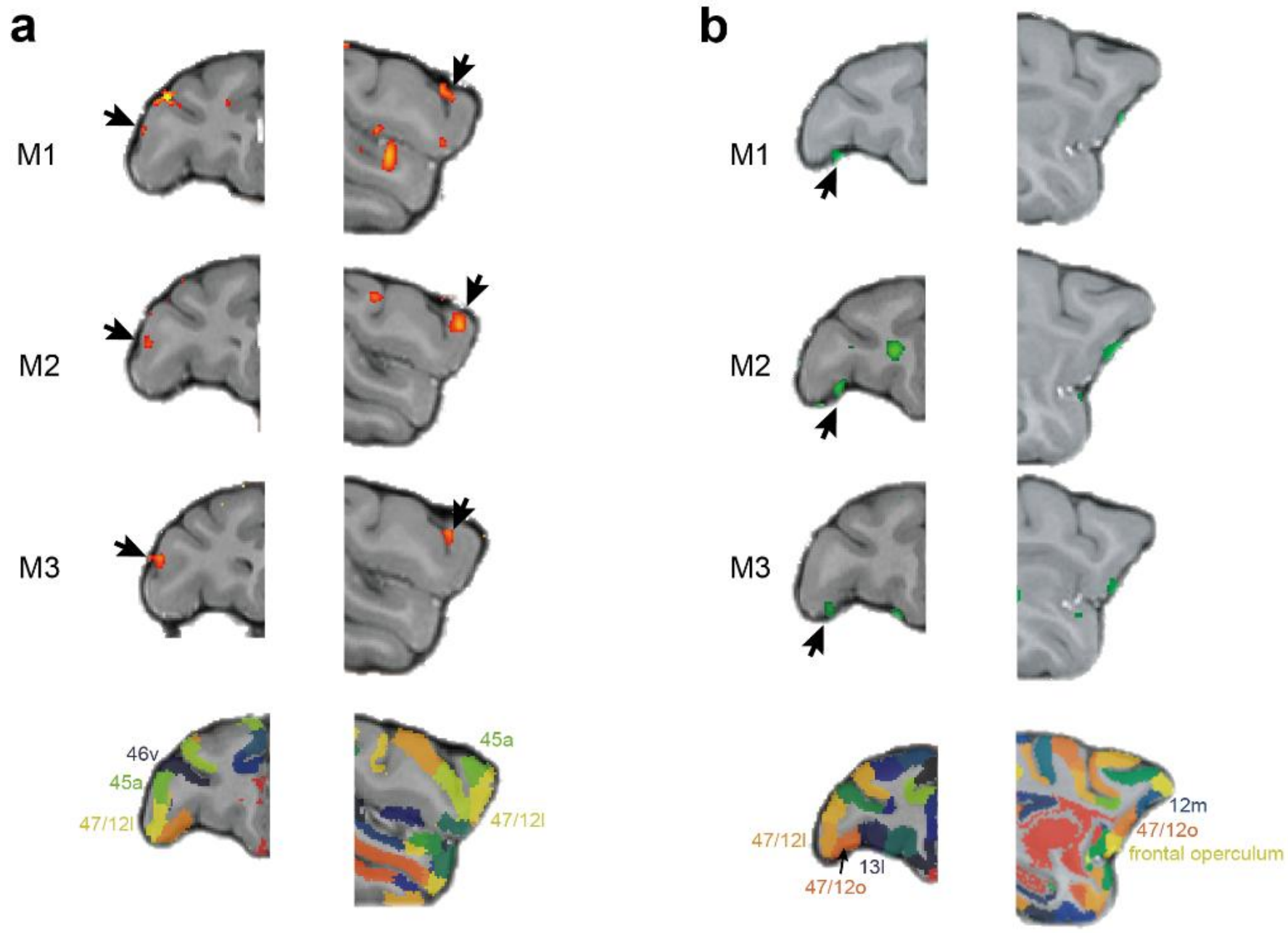

Supplementary Fig. 4. Across-animal consistency of activation peaks in area 45a and 47/12o

(a) Activition clusters of individual animals (M1, M2, M3) in response to 'chosen PD-probability – unchosen ED-probability' during the prospective judgement (p<0.05, FWE cluster-level corrected) on the coronal (left column) and sagittal (right column) slices of the right hemisphere (Z>2.0 for a display purpose) along with the border of anatomical regions (bottom panels). The activation peak was confined to area 45a in all three animals.

(b) Activition clusters of individual animals (M1, M2, M3) in response to 'ED-probability' irrespective of choice during the prospective judgement (p<0.05, FWE voxel-level corrected) on the coronal (left column) and sagittal (right column) slices of the right hemisphere (p<0.025 uncorrected for purpose of display) along with the border of anatomical regions (bottom panels). The activation peak was confined to area 47/12o for all three animals.

A key aspect of the response profile of area 45a during the first prospective judgement stage is that the latency to peak of the regression weights indexing the effect of PD-probability versus ED-probability on neural activity is different when the PD-probability option was chosen and when it was unchosen (original Fig. 2c, right panel). We have newly applied two-way ANOVA of the main effects of two PD-probability option choices (chosen, unchosen) and three individual animals (M1, M2, M3) to the peak latency data and confirmed that the main effect of the PD-option choice was significant ($F_{1,69}$=6.67, p=0.011) in the absence of any interaction with the identity of the individual animal from which the data were recorded ($F_{2,69}$=1.82, p=0.16) (revised Fig. 2c, right panel). This result supports the across-animal consistency of the activity in area 45a as predicting the PD-probability option choice. On the other hand, in line with our previous argument, no significant differences in peak beta value were consistent across animals (main effect of PD-probability option choices, $F_{1,69}$=0.66, p=0.41; interaction, $F_{2,69}$=0.57, p=0.56) (revised Fig. 2c, left panel). In other words, as previously argued, the peak effect of the PD probability option on neural activity in 45a is earlier, but not higher, when it is the option

that the macaque finally chooses.

Based on further analysis of the time course of regression weights shown in Fig. 3b, we found that the difference in latency between PD-option chosen and unchosen trials is prominent and significant in successful (correct) trials but not in failure (error) trials (original Fig. 3c). As described in the first analysis in the previous paragraph, a two-way ANOVA of the main effects of PD-option choices and individual animals revealed a significant main effect of PD-option choices ($F_{1,69}$=5.41, p=0.023) without any interaction with animal identity ($F_{2,69}$=0.70, p=0.50) specifically in success trials but not in failure trials (main effect of PD-probability option choices, $F_{1,69}$=0.52, p=0.47; interaction, $F_{2,69}$=0.21, p=0.81) (revised Fig. 3c).

We have also applied a similar two-way ANOVA to the peak beta values and the peak latencies of area 47/12o in response to chosen and unchosen ED-probability, and confirmed across-animal consistency of the group results demonstrated in original Fig. 4c: in area 47/12o the size of the peak beta values predict ED-option choice (main effect of ED-probability option choices, $F_{1,69}$=4.85, p=0.031; interaction, $F_{2,69}$=0.21, p=0.81) but the peak latencies do not (main effect of ED-probability option choices, $F_{1,69}$=3.10, p=0.082; interaction, $F_{2,69}$=0.91, p=0.40) (revised Fig. 4c). We have newly included these statistics into the manuscript to support the across-animal consistency of our findings as follows. In other words, as previously argued, the peak effect of the PD probability option on neural activity is earlier, but not higher, when it is the option that the macaque finally chooses. However, by contrast, the size of the peak effect of the ED-probability option is higher, but not earlier, in area 47/12o when the ED-probability option is finally chosen. Note that in the revised figures, shown below, the mean values for each individual animal are indicated together with the values for each individual session. This makes it easier to see all the effects of interest are consistent across individuals.

p.6

Activity linked to PD-probability increased more quickly when monkeys chose to pursue the PD-probability option as opposed to when they rejected it (latency to peak: two-way ANOVA of the main effects of two PD-probability option choices [chosen, unchosen] and three individual animals [M1, M2, M3]; main effect of the PD-option choice, $F_{1,69}$=6.67, p=0.011; interaction between the choices and animals, $F_{2,69}$=1.82, p=0.16; Fig. 2c). The latency difference was not explained by the difference in maximum peak height; the maximum beta weights at the peak were comparable regardless of whether the PD-option was chosen or unchosen (main effect of PD-probability option choices, $F_{1,69}$=0.66, p=0.41; interaction, $F_{2,69}$=0.57, p=0.56).

p.7

The difference in the latency between chosen and unchosen PD-probability peaks was significant for successful trials (main effect of PD-probability option choices, $F_{1,69}$=5.41, p=0.023; interaction between the choices and animals, $F_{2,69}$=0.70, p=0.50) but not for unsuccessful trials (main effect of PD-probability option choices, $F_{1,69}$=0.52, p=0.47; interaction, $F_{2,69}$=0.21, p=0.81; Fig. 3d).

p.8

Moreover, in contrast to the representation of PD-probability in area 45a, the latency to the maximum peak of the 47/12o modulation was less clearly modulated as a function of whether macaques chose the ED-option or not (main effect of ED-probability option choices, $F_{1,69}$=3.10, p=0.082; interaction between the choices and animals, $F_{2,69}$=0.91, p=0.40; Fig. 4c). Instead, however, the degree to which activity was related to ED-probability – the maximum beta relating the fMRI activity to ED-probability at the peak was significantly larger when macaques chose the ED-option as opposed to when it was unchosen (main effect of ED-probability option choices, $F_{1,69}$=4.85, p=0.031; interaction, $F_{2,69}$=0.21, p=0.81).

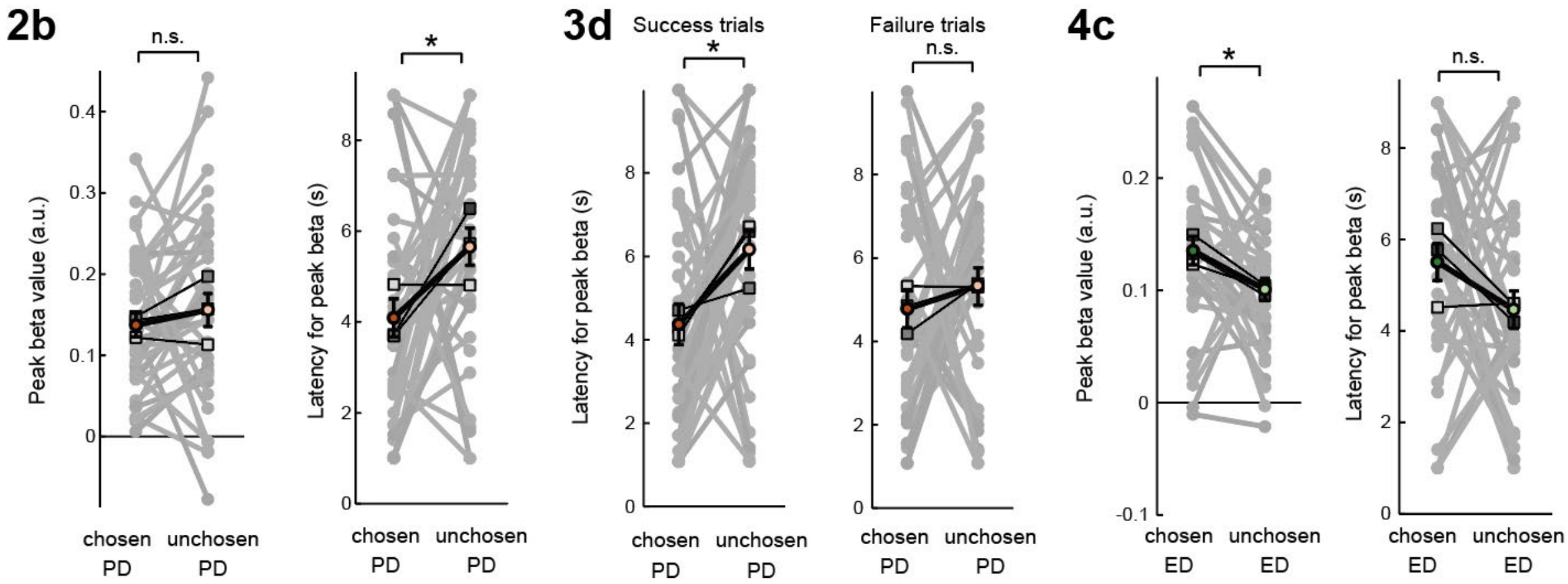

2) Also it is unclear where exactly the activation is localized: we are given a single coronal section, without any indication of areal borders. 3D brain volumes are very poor to precisely localize activations, flattened cortex with areal borders indicated would do a much better job. This the more pressing that in fig 2a the activation ascribed to area 45 seems to shift from the surface to the depth of sulcus between the chosen and unchosen PD conditions. This is intriguing given that the Lupino group has described two areas 45: 45A and 45B. The latter is in the anterior bank of inferior arcuate sulcus,next to FEF, while 45A is on the surface in front of 45B. Given the difference in anatomical connections (Gerbella et al Cerbr Cortex 2010), these two parts are considered separate areas; Also 45B, and not 45A is more visually responsive (Nelissen et al science 2005). Given the absence of any areal limit indication we are completely in the unknown about which area is activated, if any. A further problem is that the activation is described as area 45,implying bilateral activations, but the activations seems even weaker on the left side. Again we have no single subject data. We need evidence that the location of the activation, if present, is consistent in the 4 monkeys and indeed corresponds to area 45 or one of its parts. same for area 47/12°.

Response:

Thank you for the comments. We agree with the reviewer that it is critical to determine whether the activation cluster identified in Fig. 2a belongs to area 45A or 45B as these adjacent areas have different anatomical

connections and functions. First, to clarify the border of anatomical regions, we have registered the 'Cortical Hierarchy Atlas of the Rhesus Macaque (CHARM)' developed by Messinger group (Jung et al., NeuroImage, 2021; Seidlitz et al., NeuroImage, 2018), which discriminates areas 45A and 45B as described by the Luppino group (Contini et al., Eur. J. Neurosci., 2010), to our group template in CARET F99 macaque space. Then we displayed the peak of the activation cluster in response to chosen PD-probability against unchosen ED-probability and the registered anatomical demarcation map on the same coronal slice side-by-side. As shown in the new Fig S3a, the peak of the cluster is located at area 45A. Moreover, we have conducted the same analysis separately for each individual animal, the activation peak was found within area 45A (new Fig. S4a). In a similar manner we have also demonstrated the peak of the cluster in response to both of the chosen and unchosen ED-probabilities (Fig. 4a) is located at area 47/12o (new Fig. S3e).

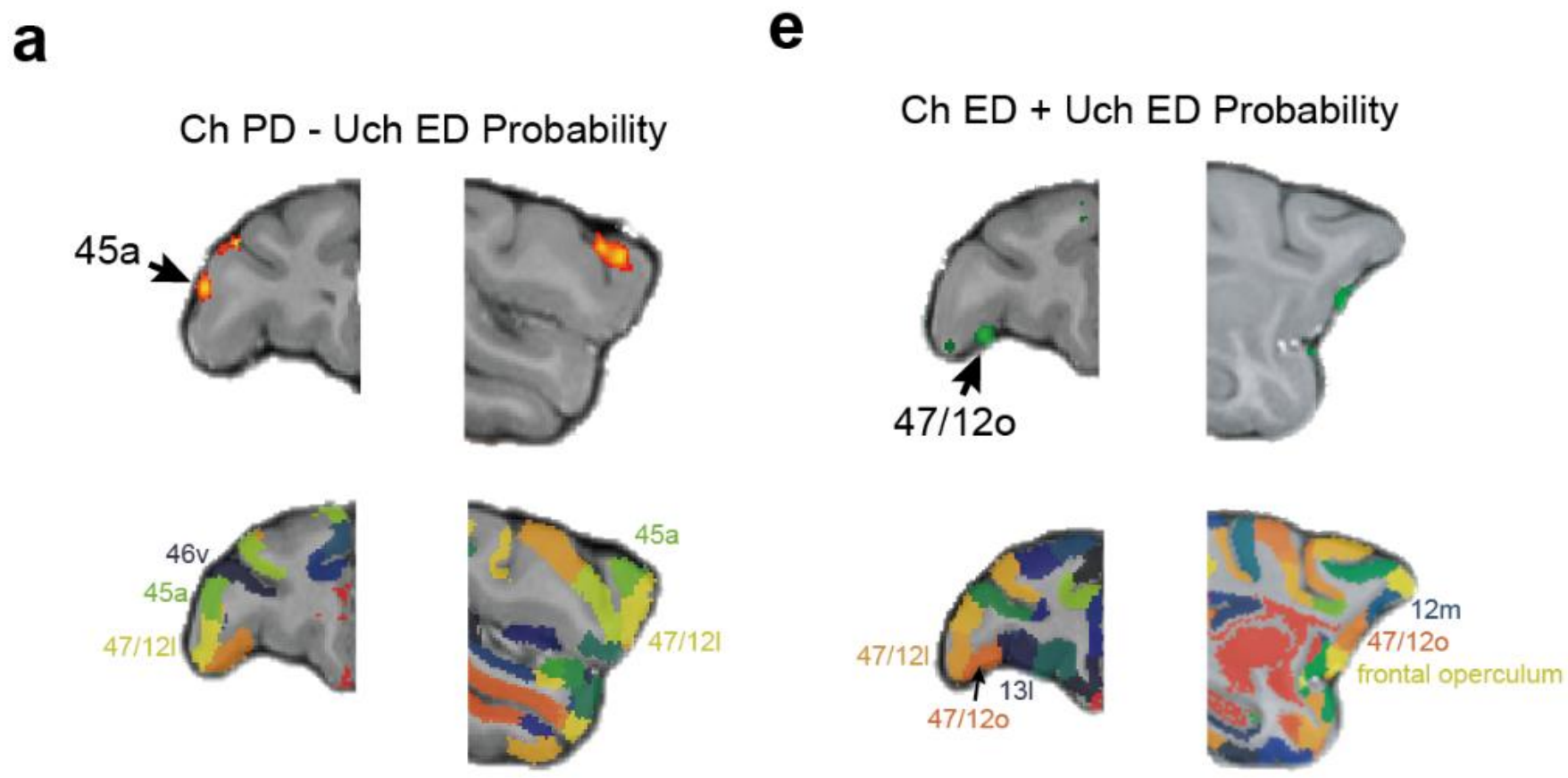

Supplementary Fig. 3. Representation of PD- and ED-probability in bilateral area 45a and 47/12o, respectively during prospective judgements

(a) Group activition clusters in response to 'chosen PD-probability – unchosen ED-probability' during the prospective judgement (p<0.05, FWE cluster-level corrected) on the coronal (top left panel) and sagittal (top right panel) slices of the right hemisphere. To clarify the borders of anatomical regions on the same slices, we have registered the 'Cortical Hierarchy Atlas of the Rhesus Macaque (CHARM)' [67,69] to our group template in CARET F99 macaque space (bottom panels). The activation peak was confined to area 45a (see also Fig. S4c–d) .

(e) Group activition clusters in response to 'ED-probability' irrespective of choice during the prospective judgement (p<0.05, FWE voxel-level corrected) on the coronal (top left panel) and sagittal (top right panel) slices of the right hemisphere along with the borders of anatomical regions (bottom panels). The activation peak was confined to area 47/12o.

In line with the reviewer's suggestion, we have also demonstrated the extent of the activation cluster for 'chosen PD-probability against unchosen ED-probability' contrast on the flattened cortex with areal borders of areas 45a and 45b based on the CHARM atlas (new Fig S4c) as well as on a series of axial slices (new Fig S4d). As seen in the top-row panels on both panel c and d below, the activation cluster of the original contrast (Fig.

2a) was on the posterior convexity anterior to the inferior arcuate sulcus which corresponds to area 45a, rather than in the anterior bank of inferior arcuate sulcus, which corresponds to area 45b. Only the cluster in the right area 45a survived after an FWE cluster-level correction in the original contrast. In response to the reviewer's question about the consistency of activations in response to chosen and unchosen PD probabilities with respect to the anatomical demarcation, we have also demonstrated the activation cluster for the 'chosen + unchosen PD-probability' contrast. As can be seen in the lower-row panels of panel d and the middle-row panels of panel d below, by using a more liberal statistical threshold without correction, it was apparent that, bilaterally, activity in area 45a, on the posterior convexity, anterior to the inferior arcuate sulcus, was found to encode both chosen and unchosen PD probability.

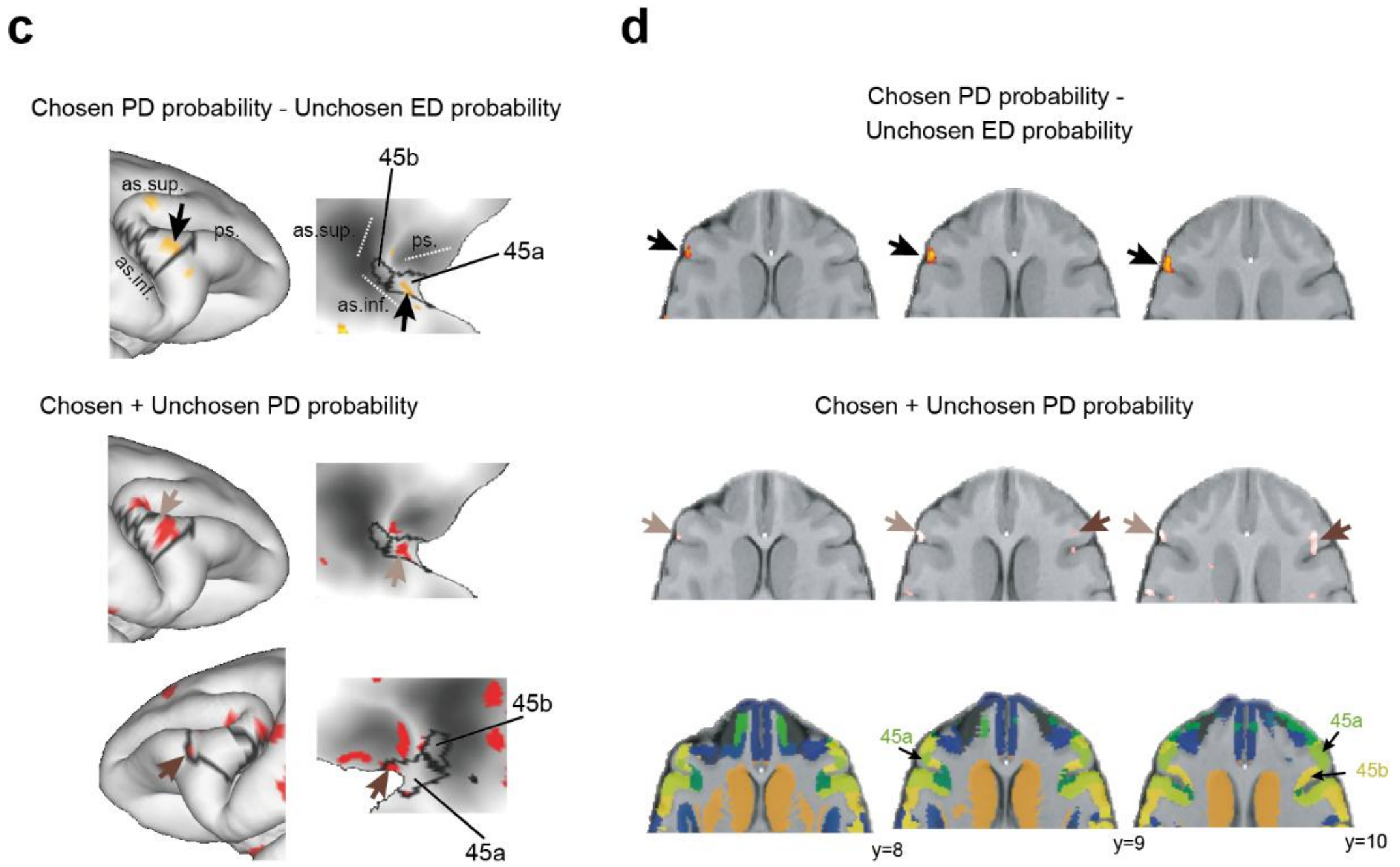

Supplementary Fig. 4. Across-animal consistency of activation peaks in area 45a and 47/12o

(c) Demonstration of group activation clusters on the 3D CARET surface (left column) and flat map (right column) in response to 'chosen PD-probability – unchosen ED-probability' ($p<0.05$, FWE cluster-level corrected) (top panels) and 'PD-probability' irrespective of choice ($p<0.025$ uncorrected for a display purpose) (bottom panels) during the prospective judgement. Activation peak was confined to area 45a rather than area 45b.

(d) Demonstration of group activation clusters in panel c on a series of axial slices.

There is activity in the right area 45a cluster and its homotopic region in the left hemisphere and it changes in proportion to the PD probability irrespective of whether the PD-option is ultimately chosen (although it should be noted, as described in the previous response, that the activity peaks with shorter latency when the PD option is ultimately chosen). We have now focused further analysis on the left activation cluster (left area 45a in new Fig. S3b and S4c–d) and compared the evolution of its activity across time (by examining the regression weights indexing the effect of PD-probability versus ED-probability on neural activity when the PD-option was chosen (new Fig. S3c, top panel) or rejected (new Fig. S3c, bottom)). In other words, the analysis

was conducted in a similar manner to that already performed on right area 45a (and shown in original Fig. 2a). Consistent with right area 45a, the peak beta value in left area 45a was comparable irrespective of whether the PD-option was chosen or not (new Fig. S3d, left panel) whereas the latency for the peak was significantly shorter when the PD-option was chosen than when the PD-option was rejected (new Fig. S3d, right panel). In other words, the same patterns of activity are present in left and right 45a. To test across-animal consistency, we have applied a two-way ANOVA of the main effects of the PD-probability option choices (when chosen versus when unchosen) and three individual animals (M1, M2, M3) in the same manner as was done for right area 45a (see new Fig. S4c). As in the right 45a, we confirmed that the main effect of the PD-option choice was significant ($F_{1,69}$=4.74, p=0.032) in the absence of any interaction with the identity of the individual animal from which the data were recorded ($F_{2,69}$=0.48, p=0.62). Moreover, again as in the right 45a, there were no significant differences in the height of the peak beta values and this was also consistent across animals (main effect of PD-probability option choices, $F_{1,69}$=0.10, p=0.75; interaction, $F_{2,69}$=0.43, p=0.65). In summary, these results confirmed across-animal consistency and bilateral similarity in left and right area 45a activation patterns in response to PD probability. We have included these results in the revised manuscript as below. For area 47/12o in the contralateral hemisphere, we have again demonstrated consistency in its activity patterns across animals below in our response to the reviewer's fourth comment. The revised figure, shown below, now indicates the mean values for each of the three animals as well as the values for each individual session.

p. 7

These characteristic features of the evolution of regression weights across time were consistent with those in the homotopic region in the left hemisphere (left area 45a) (Fig. S3b), of which activity changes in proportion to the PD probability irrespective of whether the PD-option is ultimately chosen (Fig. S3c). As in the right area 45a, we confirmed that the main effect of the PD-option choice was significant ($F_{1,69}$=4.74, p=0.032) in the absence of any interaction with the identity of the individual animal from which the data were recorded ($F_{2,69}$=0.48, p=0.62) (Fig. S3d, right panel). Moreover, again as in the right area 45a, there were no significant differences in the height of the peak beta values and this was also consistent across animals (main effect of PD-probability option choices, $F_{1,69}$=0.10, p=0.75; interaction, $F_{2,69}$=0.43, p=0.65) (Fig. S3d, left panel) .

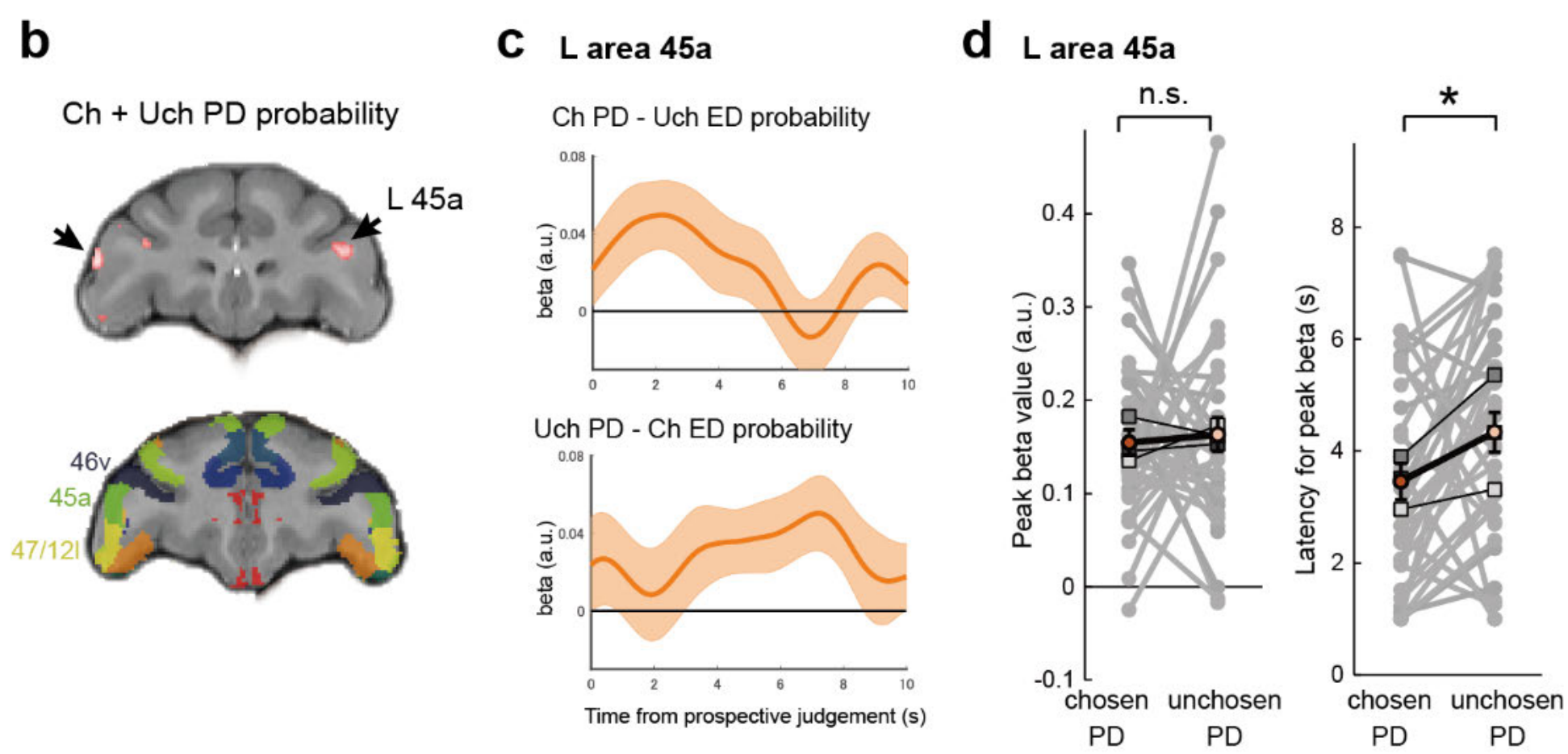

Supplementary Fig. 3. Representation of PD- and ED-probability in bilateral area 45a and 47/12o, respectively during prospective judgements

(b) Group activition clusters in response to 'PD-probability' irrespective of the animal's choice during the prospective judgement (p<0.01) (top panel) and the borders of anatomical regions on the same slices (bottom panel). The bilaretal activation peaks were confined into area 45a (see also Fig. S4c–d) .

(c) Evolution of regression weights across time, indexing the effect of PD-probability versus ED-probability on neural activity when the PD-option was chosen (top) or rejected (bottom) for the left area 45a in panel b. See also Fig 2b for the right area 45a.

(d) The difference in latency for the signal peak in the left area 45a when the PD-probability option was chosen or unchosen (right) was significant during prospective judgments, but there was no difference in the peak signal height (left). The tick line indicates the mean across sessions.

Based on these results, we have revised the nomenclature of anatomical areas. Specifically, we have replaced 'area 45' with 'area 45a' or 'area 45b', as appropriate given the context throughout the manuscript.

In response to the comment, we also replaced all displays on 3D brain volumes with the 3D CARET-based surface to illustrate the results more precisely (for example, see revised Fig 7 below).

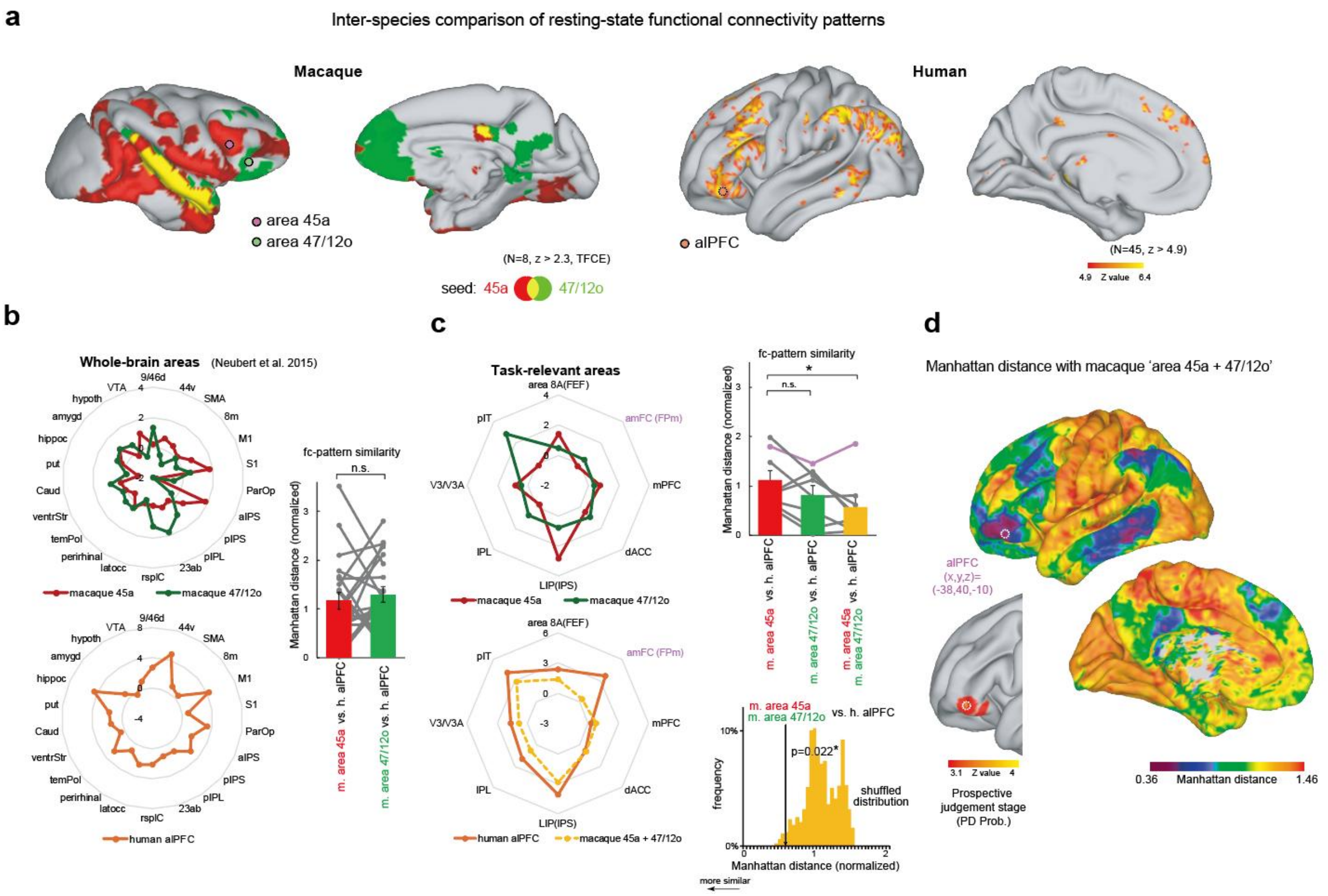

Fig. 7 Distributed neural networks linked to macaque areas 45a and 47/12o and human alPFC: inter-species commonalities and differences.

3) In general the localization of activations remains an issue by the reliance of 3D brain volumes or surface views: as an example in fig S3 the location of FEF is unclear.

Response:

In response to the reviewer's criticisms, we have now omitted all figure panels including 3D brain volumes or surface views. We have converted each of them into an appropriate display format depending upon its purpose. To clarify the location of the cluster activated during the second perceptual decision stage of each trial, we have newly made a figure panel demonstrating the activation map and anatomical demarcation map on the same axial and sagittal slices (new Fig. S3g). We confirmed that the bilateral peaks of activation clusters at that time point are located within area 8Av, which corresponds to FEF (Amiez and Petrides, 2009). We have newly discussed this point in the revised manuscript as follows.

p.7

In summary, both FEF, active during the second stage of each trial, and area 45a, which encoded future PD-probability during the first stage of each trial lie anterior to the arcuate sulcus. However, area 45a activity was ventral and anterior to activity related to performance of the perceptual decision-making task in the second stage of each trial. The bilateral peaks of activation clusters during the second stage are located within area 8Av, which corresponds to FEF[17] (Fig. S3g). The clusters of activity were non-overlapping with the activation cluster at area 45a for the prospective judgement (Fig. S5g).

g

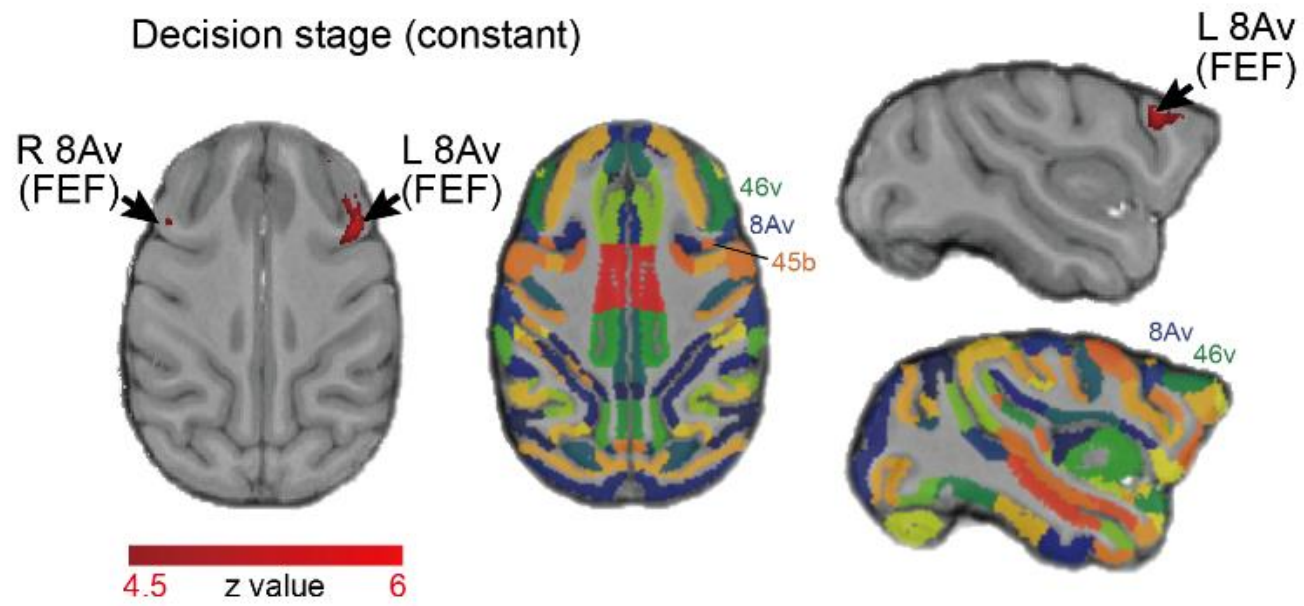

Supplementary Fig. 3. Representation of PD- and ED-probability in bilateral area 45a and 47/12o, respectively during prospective judgements

(g) The location of activation peaks ($p<0.05$, FWE voxel-level corrected) associated with the main effect (constant) of perceptual decision was identified in bilateral area 8Av, which corresponds to the frontal eye field (FEF). The activation clusters did not overlap with those of area 45a or 47/12o.

4) What is the evidence that the TUS impaired the relevant area? The only thing we are provided with (in

fig S5) is a simulation. The value of such simulation is weak. At least the authors could have manipulated the focal distance to show that the behavioral effect was maximal when the focal distance matched the location of the target area. It seems that the TUS was delivered bilaterally, how this fit with the activation which is asymmetric (fig 2)

Response:

Thank you for pointing out these issues. In our TUS experiments (Experiments 3), we targeted bilateral area 45a or bilateral area 47/12o in separate sessions. The distance between the TUS foci for area 45a and area 47/12o was 12.5 mm in each hemisphere. As we described in the original manuscript, *the impact of PD-probability on PD-option preference was significantly affected by area 45 disruption compared to area 47/12o disruption (two-way ANOVA of disruption sites and animals; main effect of disruption site:* $F_{1,35}=42.4$, $p=1.6\times10^{-7}$*), whereas the effect of ED-probability was significantly affected by area 47/12o disruption compared to area 45 disruption (two-way ANOVA of disruption sites and animals; main effect of disruption site:* $F_{1,35}=21.0$, $p=5.6\times10^{-5}$*)*. This double dissociation in itself suggests that the spatial range of the TUS effects on behaviour is much narrower than 12.5 mm.

To directly test the impacts of TUS targeted for areas 45a and 47/12o on the neural activity of these areas, we have carried out an additional experiment in which we applied bilateral TUS to either area 45a or 47/12o and compared the resting-state functional connectivity networks between pre-TUS and post-TUS scans. If the TUS directly changes activity in the stimulated area, then, as in previous studies we expect that the relative influence of activity in interconnected areas on the stimulated area will change and correlation in the activity in the stimulated area and interconnected area (Khalighinejad et al., Neuron, 2020; Fouragnan et al., Nat, Neurosci., 2019; Folloni et al., Neuron, 2019; Priestley et al., Sci. Adv., 2025; Verhagen et al., eLife, 2019) will change. This should happen regardless of any combination of excitatory or inhibitory effects that the TUS exerts. This is indeed what we found when we collected data from two sessions from each of two animals for each target area (4 sessions in total from 2 animals [M2 and M5] separately for bilateral area 45a TUS and bilateral area 47/12o TUS). As demonstrated in the new Fig. S7c, TUS targeted to area 45a prominently decreased area 45a's functional connectivity with mPFC, dACC and LIP (IPS) compared to the pre-TUS baseline (left panel). In contrast, TUS targeted to area 47/12o prominently decreased area 47/12o's functional connectivity with area 8A (FEF) and pIT (right panel). These dissociations in changed functional connectivity delivered by 45a and 47/12o TUS back up the claim that TUS does indeed truly change the activity of the targeted areas and the spatial extent of intervention was focal enough to dissociate between areas 45a and 47/12o.

c

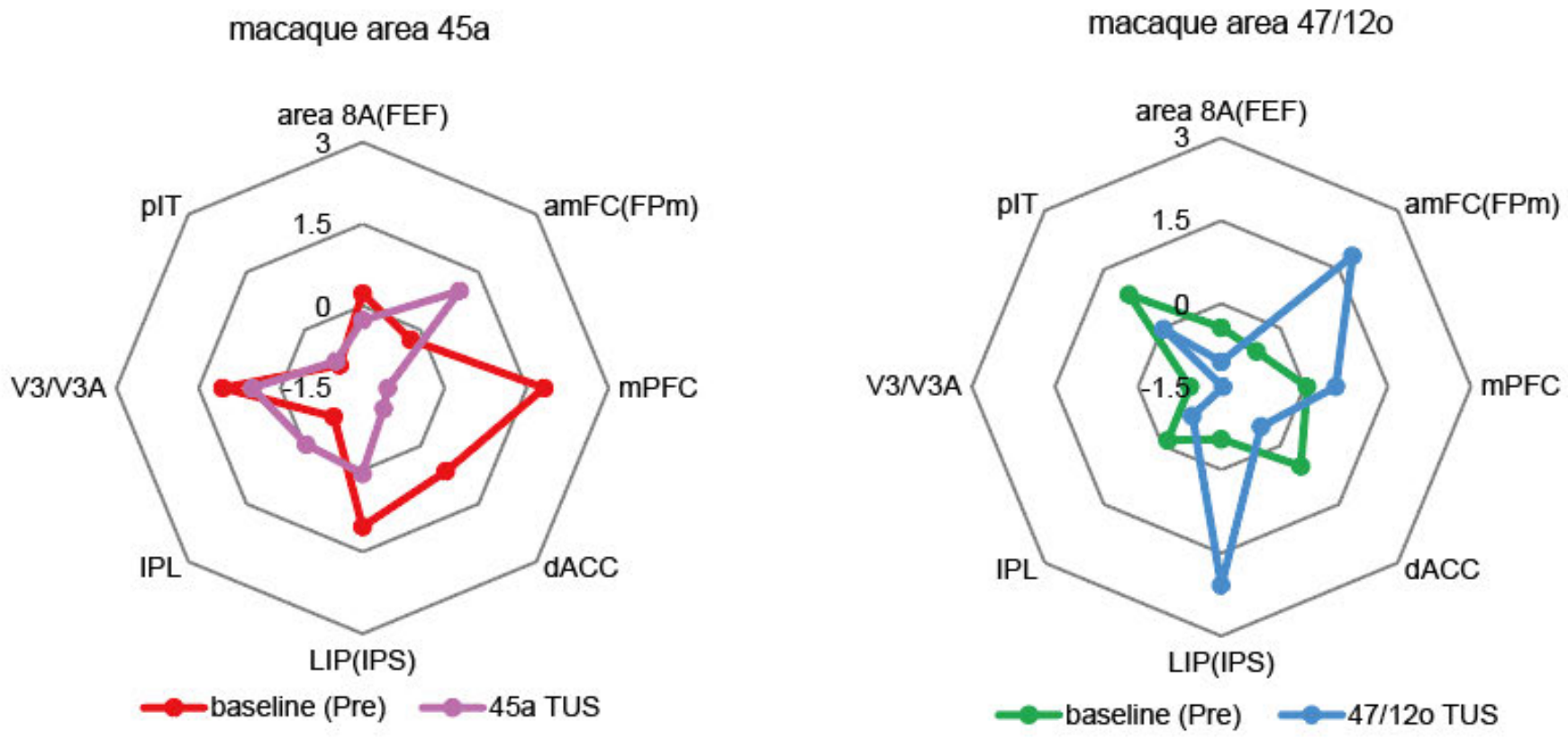

Supplementary Fig. 7. Simulated acoustic intensity of ultrasonic disruption and its causal effects on comparisons of ED-probabilities

(c) (Left) Causal effect of TUS on target brain region activity. There were changes in each region's fc with eight key task-related regions (Table S1) due to TUS targeted to the bilateral area 45a (left panel; see also panel a, top row) and area 47/12o (right panel; see also panel a, middle row). Fc-fingerprints of two sessions from each of two animals were compared between PRE- and POST-TUS. area 45a TUS decreased fc with mPFC, dACC and IPS whereas 47/12o TUS decreased fc with pIT and FEF and increased fc with amFC(FPm) and LIP(IPS). These dissociations in changed functional connectivity delivered by 45a and 47/12o TUS confirm that TUS altered activity in each targeted area and that the spatial extent of intervention was sufficiently focal to dissociate areas 45a and 47/12o.

We have discussed these points in the revised manuscript as below:

p.11

In line with the fMRI results, we found that area 45a and 47/12o made doubly dissociable contributions to evaluation of the two types of probability. The impact of PD-probability on PD-option preference was significantly affected by area 45a disruption compared to area 47/12o disruption (two-way ANOVA of disruption sites and animals; main effect of disruption site: $F_{1,35}$=42.4, p=1.6×10^{-7}), whereas the effect of ED-probability was significantly affected by area 47/12o disruption compared to area 45a disruption (two-way ANOVA of disruption sites and animals; main effect of disruption site: $F_{1,35}$=21.0, p=5.6×10^{-5}). The distance between the TUS foci for area 45a and area 47/12o was 12.5 mm in each hemisphere. This double dissociation suggests that the spatial range of the TUS effects on behaviour is much narrower than 12.5 mm.

p.13

To causally test if functional connectivity networks from the seed at macaque 45a and 47/12o are segregated with each other, we applied bilateral TUS to either area 45a or 47/12o and compared the resting-state functional connectivity networks between pre-TUS and post-TUS scans[30-34] (4 sessions in total from 2 animals [M2 and M5] for each TUS target). 45a TUS prominently decreased area 45a's functional connectivity with

mPFC, dACC and LIP (IPS) compared to the pre-TUS baseline (Fig. S7c, left panel). In contrast, TUS targeted to area 47/12o prominently decreased area 47/12o's functional connectivity with area 8A (FEF) and pIT (Fig. S7c, right panel). These dissociations in impaired functional connectivity due to 45a and 47/12o TUS support that 45a and 47/12o drives separate subnetworks of brain in macaques.

In the original fMRI analysis, by using conventional statistical criteria with FWE correction, significant activity in area 45a and area 47/12o was identified; after correction activity was only above threshold in the right hemisphere. As we have discussed above in our response to the reviewer's second comment, left area 45a was responsive to both chosen and unchosen PD-probability options in a similar manner to right area 45a once a more liberal statistical threshold was used (in other words, one applied without correction). We found that area 45a in the left hemisphere was active with a different latency when the PD option was either the chosen or unchosen option, just as was the case in the right hemisphere, and all these effects were consistent across individuals (see revised Fig. 2c and S3d). Thus, we used the peak of the left area 45a cluster as a TUS target in the left hemisphere. When we turn to area 47/12o, it is true that no clear activation clusters were found in response to ED probability in left area 47/12o but when we focused on the homotopic left hemisphere region corresponding to right 47/12o (with the same x coordinate but with the opposite sign). As shown in the new Fig. S3f below, similarly to right 47/12o (revised Fig. 4c), the size of the peak beta values at left 47/12o predicted ED-option choice and this was true consistently across the three individuals (main effect of ED-probability option choices, $F_{1,69}$=7.24, p=0.089; interaction, $F_{2,69}$=1.30, p=0.27). Thus, we used this homotopic 47/12o area as a TUS target in the left hemisphere. We have described these points in more detail in the revised manuscript as below:

p.8

No clear activation clusters were found in response to ED probability in left area 47/12o when we conducted analyses that corrected for multiple comparisons across the whole brain. However, when the homotopic left hemisphere region corresponding to right 47/12o (with the same x coordinate but with the opposite sign) was examined in a focused manner, just as in the right 47/12o (Fig. 4c), the size of the peak beta values at left 47/12o predicted ED-option choice and this was true consistently across the three individuals (main effect of ED-probability option choices, $F_{1,69}$=7.24, p=0.089; interaction, $F_{2,69}$=1.30, p=0.27) (Fig. S3f). These observations suggest that evaluation of the ED-option occurs at the same rate irrespective of final choice when animals are choosing between the PD- and ED-options (Fig. 1d) but, instead, the maximum activity level reflected whether or not the final choice would be the environmental contingency option.

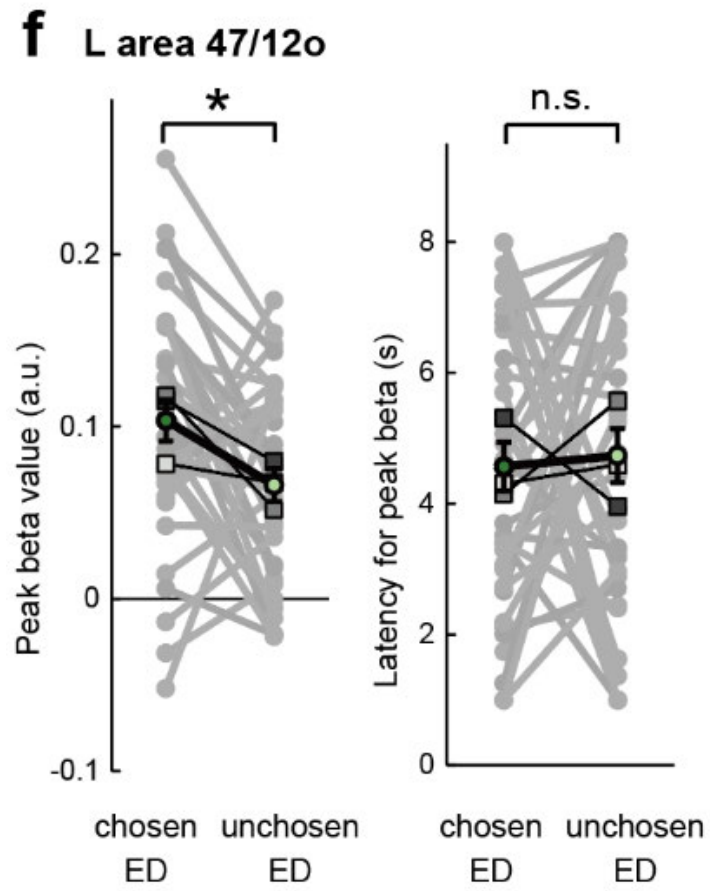

Supplementary Fig. 3. Representation of PD- and ED-probability in bilateral area 45a and 47/12o, respectively during prospective judgements

(f) Unlike in right area 47/12o, no clear activation clusters were found in response to ED probability in left area 47/12o when we corrected for multiple comparisons across the whole brain. However, when we focused on the homotopic left hemisphere region corresponding to right 47/12o ([x,y,z]=[-21.6, 14.5, -1.0]) we found that, like right 47/12o, there was a significance difference in peak signal in the left area 47/12o when the ED-option was chosen or unchosen (left). Again, as in right area 47/12o, there was no difference in latency to the peak signal in left 47/12o (right). The tick line indicates the mean across sessions.

5) The functional connectivity part is extremely weak as the underlying mechanisms of functional connectivity are very diverse, ranging from direct anatomical connections to multi-synaptic connections over 1, 2,… n stages; in addition these intervening stages are not guaranteed to be identical in different species. Hence it is very difficult to derive anything from a similarity in functional connectivity between species as done in fig 7C. A further problem is that this figure assumes homologies between human and macaque areas, for which no evidence is provided. Often this group has used functional connectivity to derive homologies between macaque and human areas, which is then pretty close to circular reasoning. In addition establishment of homologies in the parieto-occipital cortex is particularly hard, given the extension of the intraparietal sulcus into occipital cortex in humans.

Response:

As the reviewer suggests, the underlying mechanisms of functional connectivity are very diverse, ranging from direct anatomical connections to multi-synaptic connections. It is true also that caution is needed in many aspects of the analyses. We note that this is a topic on which we have written on more than one occasion (O'Reilly et al., PNAS, 2013; Mars et al., Annual Reviews of Neuroscience, 2021). However, while there are indeed theoretical reasons why functional connectivity may not reflect monosynapatic connectivity, we know that in practice, there are important points of correspondence. For example, it has previously been found that there is a systematic relationship between resting-state functional connectivity and anatomical connection strength based on a database of macaque direct axonal projection patterns (Bakker et al., 2012): area pairs

with bilateral anatomical connections yield stronger functional connectivity than the pairs with unilateral anatomical connection; area pairs with unilateral connections yield stronger functional connectivity than the pairs without anatomical connections; the number of indirect anatomical connections between directly unconnected areas is positively correlated with the strength of the functional connectivity (Adachi et al., Cereb. Cortex, 2012). We also found that subnetwork (communities) divisions determined by resting-state functional connectivity among task-related (memory retrieval) areas reproduced those based on CoCoMac axonal projection patterns (Miyamoto et al. Neuron 2013). In summary, these findings suggest that functional connectivity is one good approximation of the strength of anatomical connections between area pairs albeit taking into consideration the effects of indirect multi-synaptic anatomical connections as well as monosynaptic, direct connections, which are often correlated with one another.

As the reviewer also points out, homologies between human and macaque areas especially for the target regions of fingerprint spider-plot are critical for the analysis. To prevent potential circular reasoning, in the present study, we defined these areas based on task-based fMRI during performance of prospective judgements and perceptual decisions in both macaques and humans. The GLM contrasts identifying peak coordinates for each inter-species functional homologue area were summarized in the revised Table S1. By adopting this task fMRI-based approach, we defined the putative functional homologue areas also in the parieto-occipital cortex (e.g., LIP, IPL, V3/V3a) where prominent cortical expansion happened and anatomical structures exhibit some divergence between humans and macaques. As demonstrated in the original Fig. 7c, inter-species comparisons of functional connectivity-pattern fingerprints based on these functionally defined ROIs were more robust in identifying the functional homologue of human alPFC within the macaque brain as area 45a+47/12o than the conventional approach using a priori ROIs defined independently of the task (shown in original Fig. 7b). We have discussed these points in the revised manuscript as below:

p.12

Compared with many other human prefrontal areas, human alPFC's pattern of functional connectivity (fc-patterns) is less like that of any macaque brain area [6]. Other comparisons of prefrontal connectivity in humans and macaques also suggest the human alPFC region is distinctive [23,24]. The fc-pattern is one good approximation of the strength of anatomical connections between area pairs albeit taking into consideration the effects of indirect multi-synaptic anatomical connections as well as monosynaptic, direct connections, which are often correlated with one another[25-28].

pp.12-13

Next, we carried out a similar analysis but now focusing on the areas' fc-patterns with only other task-related, as opposed to task-unrelated, areas (Table S1), especially areas for perceptual decision making. The homologies between human and macaque areas for the target regions of fingerprint spider-plot, especially where prominent cortical expansion happened and anatomical structures exhibit some inter-species divergence, are critical for the inter-species comparisons of fc-pattens. Using target regions identified by task-based fMRI during performance of similar prospective judgements and perceptual decisions in both macaques and humans

ensures their functional correspondence. Again, macaque area 45a was no closer to human alPFC than macaque 47/12o (t_7=1.85, p=0.10). However, the fc-network for the combination of both macaque areas 45a and 47/12o was very similar to the human fc-network centered at alPFC (Fig. 7c left, upper and lower panels).

Supplementary Table 1. Eight key regions along with macaque areas 45a and 47/12o and human alPFC, which were determined based on the brain activity during the current task

seed	coordinate		contrast	
	macaque (F99)	human (MNI)	macaque	human
area 45a	(21.1, 7.5, 12.5)	n/a	Pros Ch. PD – Uch. ED	n/a
area 47/12o	(21.6, 14.5, -1.0)	n/a	Pros ED	n/a
alPFC	n/a	(-38, 40, 10)	n/a	Pros PD
area 8A(FEF)	(19.5,10,13)	(-22,12,54)	Dec (const)	Dec Ch. PD & ED
amFC(FPm)	(4.5,27,9)	(-2,52,22)	Pros Ch. ED – Uch. ED	Dec Ch. PD
mPFC	(0,22,6)	(-12,54,4)	Dec (const)	Dec Ch. PD & ED
dACC	(1,17,12)	(-6,22,36)	FB ED	Pros Uch. – Ch. PD & ED
LIP(IPS)	(14.1, -22.1,18.6)	(-36,-70,52)	Dec (const)	Dec Ch. PD & ED
IPL	(12,-36,20)	(-42,-72,26)	Dec (const)	Dec Ch. PD & ED
V3/V3a	(28,-27,3)	(-22,-88,20)	Pros Ch ED	Pros Ch. PD & ED
pIT	(29,-7,-4)	(-66, -36, -10)	Pros Ch PD – Uch ED	Pros PD – ED

fMRI activity at the coordinates in the table were used as the seed of functional connectivity (FC) analysis for inter-species FC-patterns comparison. Dec indicates that the contrast pertains to second stage, perceptual decisions, Pros indicates that a contrast pertains to the first part of the trial – the prospective judgement stage and FB indicates that the contrast pertains to outcome feedback stage. Ch.: chosen, Uch: unchosen, PD: Performance-dependent option, ED: Environment-dependent option.

6) in the discussion the authors interpret their data to mean that the two areas 45 and 47° of the macaque fuse into a single medial prefrontal area in humans; This is an extremely unlikely scenario; Given the 10 fold increase in cortical surface in humans compared to macaque, it is generally assumed that cortical areas of the macaque split into two and yield two human areas, not the reverse. A more likely scenario (if the data are proven to be correct) would be that area 47/12° of the macaque gives raise the medial prefrontal area of humans, and that the selectivity of macaque 45 to PD signals moved to this human area, because the homologue of 45 (or its parts) is colonized by novel afferents, probably those related to semantic or linguistic processing. Such a scenario would fit the large expansion of cortex in medial prefrontal cortex and the presence of a uniquely human area dACC (based on functional connections but combined with functional responses!) in this region (fig 10, Mantini et al J Neurosci 2013).

The reviewer is correct that it is extremely unlikely that areas that are distinct and separate in the macaque

have fused together into single areas in the human brain during the course of speciation. Moreover, this is not the argument that we are making. Rather the scenario for which we argue is one that parallels the types of processes that have been documented elsewhere during the course of speciation (Krubitzer and Seelke, PNAS, 2012). What we propose is, first, that since the time of their common ancestor, the cortex that corresponds to 47/12o in the macaque is, in humans, likely to have expanded during the course of human evolution. Second, we argue that anatomical projections that may have been confined to area 45a in the macaque may extend beyond area 45a into adjacent areas such as area 47/12o. As the reviewer puts it, a part of 47/12o is "colonized" by these connections. We, indeed, think that, in humans, there are other areas that are successors to an ancestral 47/12o region and that some of these are not colonized by projections associated with area 45a in the macaque. In summary, we think that the argument we are making has key points of convergence with that put forward by the reviewer and we have tried to make these clearer in the revised manuscript as shown below:

p.15

We propose that since the time of their common ancestor, the cortex that corresponds to 47/12o in the macaque is, in humans, likely to have expanded during the course of human evolution. As a result, anatomical projections that may have been confined to area 45a in the macaque may extend beyond area 45a into adjacent areas such as area 47/12o in humans. A part of 47/12o might have been 'colonized'[47] by these connections and developed as human alPFC, while other human areas that are also successors to an ancestral 47/12o region may not have been colonized by projections associated with area 45a in the macaque.

Another way to think about this argument we are making is that if alPFC is indeed an evolutionarily novel area unique to humans and with a specialized function, which has arisen during brain expansion, then how should we expect macaques' brain to be active when the macaques are performing a task that in humans depends on alPFC? The macaques cannot perform the task by activating a single homologue of human alPFC if they do not possess it. Instead, we should expect that if the macaques are able to perform the task at all, then they may well do so via the assistance of activity patterns in more than one of the less specialized brain areas they do possess. We have also tried to make this argument in the revised manuscript as shown below:

p.15

In other words, if alPFC is indeed an evolutionarily novel area unique to humans and with a specialized function, macaques cannot perform the task by activating a single homologue of human alPFC if they do not possess it. Instead, we should expect that if macaques are able to perform the task at all, then they may well do so via the assistance of activity patterns in more than one of the less specialized brain areas they do possess, such as areas 45a and 47/12o.

A minor point is the lack of clarity of the description of the two behavioral tasks; it took me a long time to understand them and fig 1 did not help, to the contrary.

Guy Orban

We have tried to make the descriptions of the behavioural tasks clearer in the revised manuscript as follows. However, we note that the tasks are complex ones that are designed to isolate specific cognitive processes.

p.18

… All combinations of the seven ED-probability levels (environment-vs-environment task) or of the seven PD-probability levels and seven ED-probability levels (performance-vs-environment task) were offered during the first stage of each trial – the prospective judgment stage. In the second stage following this prospective judgement, animals were given a chance to perform the task they chose, in which they touched either a left or right sensor box depending on the direction in which most dots moved (the 'coherent' dot direction). The number of dots in the ED-probability task indicated the 'environmental probability' of receiving a reward when the correct response was made; if there were more (fewer) dots then the probability of reward was higher (lower). This ED-probability was independent of the animal's own performance levels. The coherence of the synchronized dot motions in PD-probability task, however, determined the animal's performance levels and, therefore, it determined the PD-probability of whether a reward would be received; they were more likely to perform correctly when coherence levels were high as opposed to low. To maximize the chance of reward to be obtained after the second perceptual decision stage, animals were required to compare the ED- or PD-probability of task options offered during the prospective judgement stage.

Reviewer #2 (Remarks to the Author):

Miyamoto K et al., Neural recording, disruption, and connectivity comparisons identify origins of human metacognition in other primates

The authors describe a study in which they used fMRI to examined prospective decision confidence judgements in macaques and humans. In the task, subjects were shown two stimuli, one in which there were many dots, but the coherence level varied from trial-to-trial (the PD stimulus) and the other in which the number of dots varied (which signaled the probability of receiving a reward for a correct choice) but the coherence level was 1, such that the perceptual inference was easy. The subjects had to choose one of these tasks at the first stage, after which they were given the chosen decision-making task at the second stage. Monkeys and humans showed that they could predict future performance levels at the first stage, and made choices that took into account both their PD performance, and the ED level. The monkeys were not as good at predicting performance for difficult PD trials. Analysis of the imaging data showed that macaque area 45 was related to the PD option, and activity peaked earlier on trials in which the PD option was chosen. Area 47/12o, on the other hand, was related to the ED option. There were no latency differences in area 47 in chosen vs. unchosen ED trials.

In general, this study addresses important questions about not only homology across macaque and human prefrontal cortical areas, but also decision processes within primate prefrontal cortex related to metacognition. I found the paper to be very well written and straightforward. The use of TUS to disrupt processing within each area also substantially increased the impact of the work. I have several questions that should be clarified, however.

Comments

1. For the analysis in Figure 1g, it also seems likely that the animals may be assessing their willingness to exert effort in the decision stage. If they are willing to exert more effort, they will likely do better. On the contrary, it is easy to simply make an easy inference in the decision stage, and then accept the ED outcome. This should at least be discussed.

Response:

Thank you for pointing out this issue. In the analysis in Figure 1g, it is important that we compare the performance in the PD-option task between challenge and inevitable trials only when the animal actively chose the PD-option against the ED-option during the preceding prospective judgement stage. The probability of reward to be acquired by taking the PD-option choice at a given coherence level is determined by both the objective visual features of the PD-option stimuli (trial-by-trial difference in the appearance of dot motion patterns) and the animal's subjective internal state fluctuating across trials that includes mental conditions and willingness to perform the task. Therefore, we agree with the reviewer that assessments of willingness to exert

effort on the offered PD-option might influence the animals' PD-option choice. However, the proportion of PD-option choice in ED-option optimal trials on the session basis, which reflects the general willingness, within a session, to intentionally choose the economically suboptimal PD-option, did not predict session-based improvement in PD-option task performance in challenge trials compared to inevitable trials (r=0.10, p=0.46; new Fig. S1d). Thus, it is unlikely that animals' assessments of their general willingness to exert effort in the decision stage fully explains the better performance of the PD-option task in challenge trials. A key component of what drives the PD-option choices must be related to trial-specific prospection regarding subtle variation in their likely ability to perform a PD-option at a specific time. We have newly discussed these points in the revised manuscript as follows:

p.6

This behavioural result suggests monkeys assess their own likely performance levels in the first stage of each trial to predict PD-probability that is specific to the second stage of each trial. Notably, the proportion of PD-option choice in ED-option optimal trials on the session basis, which reflects the general willingness, within a session, to intentionally choose the economically suboptimal PD-option, did not predict session-based improvement in PD-option task performance in challenge trials compared to inevitable trials (r=0.10, p=0.46; Fig. S1d). Thus, it is unlikely that animals' assessments of their general willingness to exert effort in the decision stage fully explains the better performance of the PD-option task in challenge trials. A key component of what drives the PD-option choices must be related to trial-specific prospection regarding subtle variation in their likely ability to perform a PD-option at a specific time.

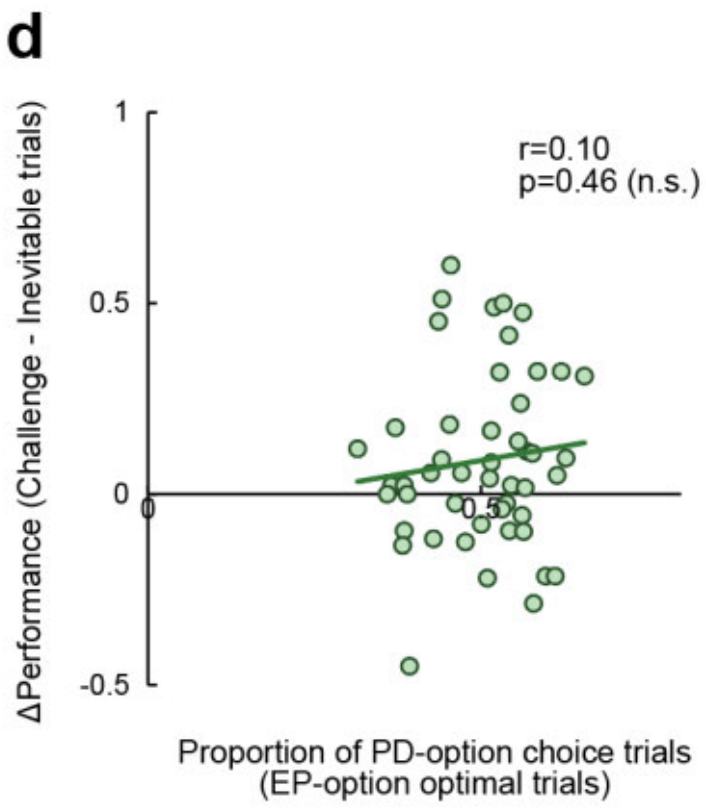

Supplementary Fig. 1. Across-animal and inter-species consistency of prospective judgements about future PD-probabilities and ED-probabilities

(d) Proportion of PD-option choice in ED-option optimal trials in each session does not predict the improvements in perceptual performance seen in challenge trials compared to inevitable trials in the same sessions (see Fig. 1g). This suggests that animals' general willingness to exert effort in the decision stage could not explain the improvements seen when animals picked the PD option on challenge trials.

2. Could eye movements account for latency differences in area 45? If the animals look at the PD option earlier in the trial, or spend more time looking at the PD option when it is ultimately chosen, this would

likely drive correlations with the coherence level higher faster. This has been shown for other decision processes in OFC.

Response:

During the fMRI experiment, we monitored the animals by using an eye tracker but did not record eye movement data for offline analyses. To answer the reviewer's question, we have focused on the left frontal eye field area (FEF) identified as having a constant effect during the perceptual decision stage (revised Fig. S5a [original Fig. S3a]; in other words, FEF activity was time-locked to the second perceptual decision stage of each trial regardless of the values of any specific, parametrically varied task features). The FEF is known to have an important role in eye movement. As seen in revised Fig. S5b (original Fig. S3b), when we carried out additional analyses, activity in FEF was found to be negatively modulated in correlation with PD-probability, with a peak latency of around 5s consistently between the first prospective judgement and second perceptual decision stages. This suggests that activity in FEF could reflect the degree of effort to be paid during motion direction discrimination; less effort is needed when there is a high PD-probability option and discernment of the motion direction is easy. We then compared this FEF modulation during the prospective judgement stage when the PD option was chosen or unchosen. We found that there are no clear differences in FEF activity as a function of whether the PD option was chosen or unchosen as shown below (new Fig. S5c). Moreover, session-by-session difference in FEF activity (in the effect of PD probability when the option was chosen versus unchosen) could not explain the latency difference in area 45a (in response to another reviewer's comments, we now call 'area 45' in the original manuscript 'area 45a') ($r=0.048$, $p=0.78$) (new Fig. S5d). These results suggest that latency differences in activity modulation in area 45a between occasions when the PD option is chosen versus unchosen cannot be explained by eye movement-related activity in FEF. We have newly discussed these points in the revised manuscript as follows:

p.7-8

The activity in FEF was found to be negatively modulated in correlation with PD-probability, with a peak latency of around 5s consistently between the first prospective judgement and second perceptual decision stages (Fig. S5b). This suggests that activity in FEF could reflect the degree of effort to be paid during motion direction discrimination; less effort is needed when there is a high PD-probability option and discernment of the motion direction is easy. We then compared this FEF modulation during the prospective judgement stage when the PD option was chosen or unchosen. We found that there are no clear differences in FEF activity as a function of whether the PD option was chosen or unchosen as shown below (Fig. S5c). Moreover, session-by-session difference in FEF activity (in the effect of PD probability when the option was chosen versus unchosen) could not explain the latency difference in area 45a ($r=0.048$, $p=0.78$) (Fig. S5d). These results suggest that latency differences in activity modulation in area 45a between occasions when the PD option is chosen versus unchosen cannot be explained by eye movement-related activity in FEF.

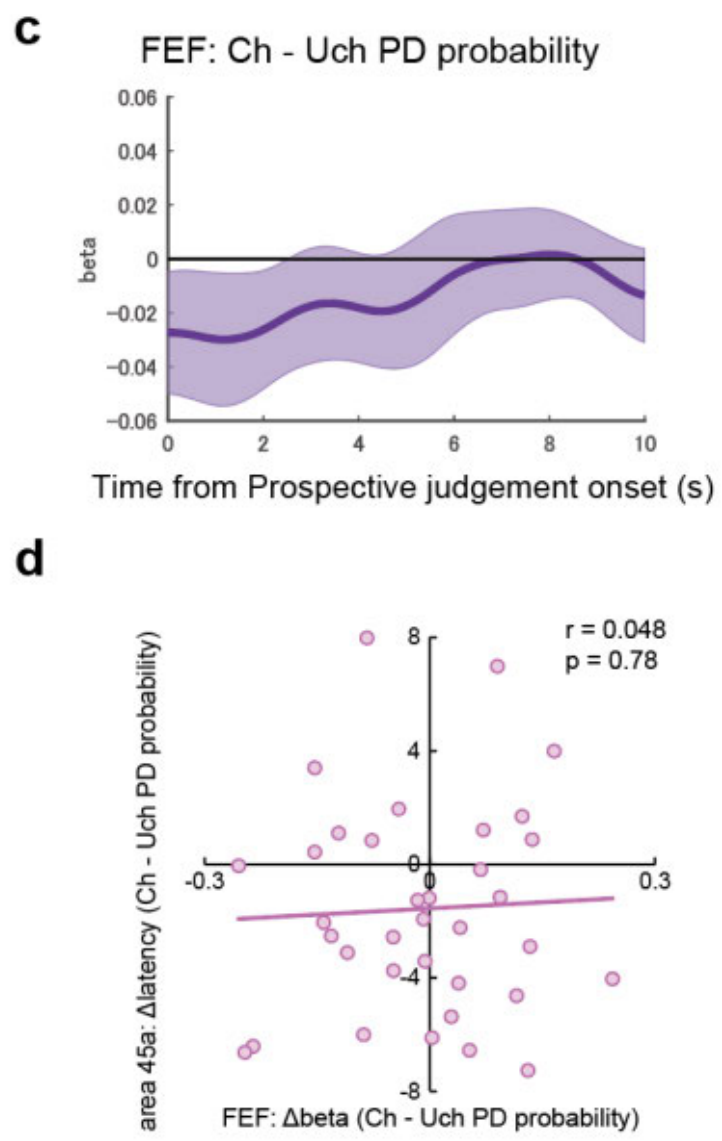

Supplementary Fig. 5. Activity correlated with PD- and ED-probabilities during the second stage of trials when perceptual decisions are made and outcomes (reward / error) occur.

(c) Comparison of FEF modulation during the prospective judgement stage when the PD option was chosen or unchosen.

(d) Session-by-session difference in FEF activity (for the effect of PD probability when the option was chosen versus unchosen) could not explain the latency difference in area 45a (r=0.048, p=0.78). The result suggests that latency differences in activity modulation in area 45a between occasions when the PD option is chosen versus unchosen cannot be explained by eye movement-related activity in FEF.

3. "First, as demonstrated in Fig. S4a, the signal in area 45 was modulated by the PD-probability in a different way when that option was chosen and when the ED-option was chosen even though, on average, the PD- and ED-options have the same reward probabilities." However, they do not have the same subjective reward probabilities. I don't think it's necessary to redo all analyses with subjective reward probabilities, but somehow this statement should be qualified?

Response:
Thank you for carefully assessing our statement. The reviewer is right that the subjective reward probability was different between PD-option and ED-option as demonstrated in the original Fig. S2a. As we have already shown in the original Fig. S2b, the signal in area 45a was not modulated by subjective probability irrespective of PD- or ED-options. On the other hand, original Fig. S4a (now revised Fig. S6a) demonstrates that the signal in area 45a is activated specifically in correlation with PD probability but not with ED probability, in contrast to the way in which activity is modulated in area 47/12o which is activated in correlation with ED probability but not with PD probability. Based on these findings, we revised the sentence as follows:

p.9

First, as demonstrated in Fig. S6a, the signal in area 45a was modulated by the PD-probability but not by ED-probability even though PD- and ED-probabilities have a linear relationship with subjective reward probabilities (Fig. 2a) in a similar manner.

4. "For the TUS experiments we returned to using the two versions of the task in which animals either chose between ED-options…" The ED/ED task was explained in the figure legend, but not in the main text. Some mention of it could be given in the main text of the results. Otherwise it comes out of the blue in this sentence, much later in the results.

Response:

Thank you for this comment. In the original manuscript, we explained the ED/ED task as follows (p.3): *First, we examined if the animals (N=15 sessions from three animals tested outside the MRI scanner) could compare two options with different levels of future ED-probabilities and choose the better one (Fig. S1a)*. By following this comment raised by the reviewer, we have updated the sentence as follows:

p.3

First, we examined if the animals (N=15 sessions from three animals tested outside the MRI scanner) could compare two options with different levels of future ED-probabilities and choose the better one. In the task, animals were given two ED-probability task options which contained a different number of dots to each other in a blue color moving downward. After they chose the left or right ED-probability task option, the chosen option moved to the center of the screen and all the dots included in the option moved either leftward or rightward. If they judged the dot direction correctly (the task was easy and they rarely failed this perceptual decision), animals were given a reward in accordance with the probability indicated by the number of dots in the chosen ED-option (Fig. S1a).

5. As the authors know, it is normally assumed that as brains expand, a single area gets larger before it splits into two sub-areas that then can take on disparate functions. The current results are inconsistent with this standard model. It would be worth at least discussing this normal account of homology in the context of the current results.

Response:

The reviewer is correct that it is extremely unlikely that areas that are distinct and separate in the macaque have come together into single areas in the human brain during the course of speciation. However, this is not the argument that we are making (although with hindsight, we can see that perhaps we were not clear enough in our writing). Rather the scenario for which we argue is one that parallels the types of processes that have been documented elsewhere during the course of speciation (Krubitzer and Seelke, PNAS, 2012). What we propose is, first, that since the time of their common ancestor, the cortex that corresponds to 47/12o in the

macaque is, in humans, likely to have expanded during the course of human evolution. Second, we argue that anatomical projections that may have been confined to area 45a in the macaque may extend beyond area 45a into adjacent areas such as area 47/12o. In other words, in humans, a part of 47/12o is "colonized" by the connections from the adjacent area 45 in a manner reminiscent to that described by Krubitzer and Seelke (PNAS, 2012). We, think that, in humans, there are other areas that are successors to an ancestral 47/12 region and that some of these are not colonized by projections associated with area 45 in the macaque. We have tried to make our argument clearer in the revised manuscript as shown below:

p.15

We propose that since the time of their common ancestor, the cortex that corresponds to 47/12o in the macaque is, in humans, likely to have expanded during the course of human evolution. As a result, anatomical projections that may have been confined to area 45a in the macaque may extend beyond area 45a into adjacent areas such as area 47/12o in humans. A part of 47/12o might have been 'colonized'[47] by these connections and developed as human alPFC, while other human areas that are also successors to an ancestral 47/12o region may not have been colonized by projections associated with area 45a in the macaque.

Another way to think about this argument we are making is that, if alPFC is indeed an evolutionarily novel area unique to humans and with a specialized function, which has arisen during brain expansion, then how should we expect macaques' brain to be active when the macaques are performing a task that in humans depends on alPFC. The macaques cannot perform the task by activating a single homologue of human alPFC if they do not possess it. Instead, we should expect that if the macaques are able to perform the task at all, then they may well do so via the assistance of activity patterns in more than one of the less specialized brain areas they do possess. We have also tried to make this argument in the revised manuscript as shown below:

p.15

In other words, if alPFC is indeed an evolutionarily novel area unique to humans and with a specialized function, macaques cannot perform the task by activating a single homologue of human alPFC if they do not possess it. Instead, we should expect that if macaques are able to perform the task at all, then they may well do so via the assistance of activity patterns in more than one of the less specialized brain areas they do possess, such as areas 45a and 47/12o.

6. "further" is repeated twice in this sentence: "To further examine judgements in the first stages of trials further we carried out logistic multiple regression analyses…"

Response:
Thank you. We have corrected.

Reviewer #3 (Remarks to the Author):

I have reviewed this paper twice before for another journal. The authors have now largely reworked the paper to address previous concerns raised during those rounds of detailed review, and I think the current version of the paper will make a strong contribution to the literature. This version has been somewhat reworked to highlight the contrast between the macaque and human findings on the same task, which is also a strength.

Thank you very much for highly evaluating our study.

My one remaining recommendation when reading the current version is that some statistical comparisons are needed between macaques and humans if these claims are to be robustly substantiated. Eg. On p.4, the differences in PD-related choices between species should be supported with a test of the condition x species interaction. And again at the top of p.5 – it's not enough to say that the PD beta was smaller than the ED for macaques but not in humans – we need a formal test of the group x condition interaction.

Response:

Thank you for this suggestion. To formally compare the size of beta coefficients between PD- and ED-options across species, we calculated the ratio of the unsigned beta for the ED-option against the PD-option for each of the 48 sessions performed by macaques and by the 23 human participants. The ratio was significantly larger than 1 (i.e., the size of the beta for the ED-option was larger than that for PD-option) in macaques (t_{47}=3.85, p=3.4x10^{-4}) whereas it was not significantly different from 1 in humans (t_{22}=1.31, p=0.20). Moreover, the ratio was significantly different between macaques and humans (t_{69}=2.32, p=0.023). These statistical analyses have support the claim that the PD beta was smaller than the ED beta in macaques but not in humans. We have newly discussed this result in the revised manuscript as follow:

p.5

In summary, macaques do appear to be reliably able to make prospective judgements about future PD-probabilities. Nevertheless, they are not as good at doing this as humans. In macaques, the unsigned beta for PD-probability was significantly smaller than the unsigned beta for environmental-dependent probability (t_{47}=-3.66, p=0.00063). In contrast, in humans, the unsigned beta was comparable between PD- and ED-options (t_{22}=-0.98, p=0.33). To formally compare the size of beta coefficients between PD- and ED-options across species, we calculated the ratio of the unsigned beta for the ED-option against the PD-option for each of the 48 sessions performed by macaques and by the 23 human participants. The ratio was significantly larger than 1 (i.e., the size of the beta for the ED-option was larger than that for PD-option) in macaques (t_{47}=3.85, p=3.4x10^{-4}) whereas it was not significantly different from 1 in humans (t_{22}=1.31, p=0.20). Moreover, the ratio was significantly different between macaques and humans (t_{69}=2.32, p=0.023). These observations statistically support our argument that the strength of influence of PD-probability is relatively smaller on macaque decisions compared to human decisions.

<u>**Responses to the editors and reviewers**</u>

Reviewer #1:

Remarks to the Author:

The authors performed pioneering behavioral studies in metacognition in macaques and combined this with fMRI, for comparison with similar data in humans. This is highly commendable. In addition in their revision they performed several control analyses and a control experiment with TUS, which strengthen the results of the first two parts of the manuscript.

In part 1, the authors should be more careful in describing the fMRI signal; This is an hemodynamic signal that only indirectly reflects neural activity (which is electrical); Many readers of Nat Hum Beh may not be aware of this distinction and will be misled by the expression 'neural activity' which is used in the main text and also the title. It is strongly recommended to use hemodynamic signal or perhaps brain activity but not neural activity.

Response:

Thank you for highly evaluating our revision. According to the reviewer's suggestion, to clarify that we recorded fMRI signals rather than neuronal spiking activities, we have replaced the term 'neural activity' with hemodynamic signal or brain activity throughout the manuscript when we refer to the signal recorded by functional MRI. The title of the manuscript is now "*Brain activity, disruption, and connectivity comparisons identify origins of human metacognition in other primates*."

The measure of hemodynamic vs electrical signals has another important consequence: given the long time constant of the hemodynamic filter: a time course difference in fMRI signal, as reported here, is hard to interpret: an earlier latency of the fMRI signal can indeed reflect an earlier neuronal response (as implied by the authors) but equally well a steeper rising neuronal response. It is important to point out this ambiguity in the discussion.

Response:

Thank you for pointing out the issue. As the reviewer suggests, steeper rising neuronal responses could be the cause of the observed earlier latency of the fMRI signal as well as the earlier neural responses. It is also noteworthy that the time courses of signals we demonstrated throughout the manuscript are not the raw hemodynamic signals but evolution of regression weights across time, indexing PD- and/or ED-probabilities, on the hemodynamic signal; therefore, the slope in the time course reflects the degree of modulation by these probabilities on hemodynamic signals. Thus, if the spiking activities were sensitive to the estimation of PD- and/or ED-probabilities during the prospective judgement, the modulation could be detected from a very early stage of the spike-triggered hemodynamic response, even though they are much slower and more sluggish compared to the source neuronal signals. By following the reviewer's suggestion, we have newly summarized

these arguments in the Discussion section as follows:

The weaker capacity for prospective judgements concerning future performance that is seen in macaques might reflect the absence of a unitary anatomical locus for interactions between activity reflecting estimates of future performance and estimates of environmental contingencies. Instead, whether macaques took the PD-probability option or the ED-option reflected the relative timing of activity in areas 45a and area 47/12o. Activity linked to performance levels ramped up more quickly when macaques took that option than when they rejected it (Figs. 2, 3, 5a-c). By contrast, the timing of activity linked to evaluation of ED-probabilities in area 47/12o did not change regardless of which choice was made. The combination of different neural activity patterns in the two areas meant that area 45a activity encoding the PD-probability option preceded (followed) the ED-option when the PD-options was chosen (rejected). We did not record the spiking activity of the neurons in these areas directly but rather we measured the hemodynamic responses that were triggered by neural activity. Such hemodynamic responses are, therefore, slower than the triggering neural activity. It should be noted that the slope and latency of the signal time courses that we report (Figs. 2b–d, 3, 4b–c) reflect the degree of modulation by PD- and/or ED-probabilities on hemodynamic responses rather than simply the raw hemodynamic responses themselves. Thus, an earlier latency of a signal in such an analysis means that there was likely to have been spiking activity in the area that was sensitive to the estimation of that particular type of probability, which could be detected from a very early stage during the prospective judgement.

In part 2, the TUS control experiment (much appreciated) indeed indicates that TUS has the appropriate spatial resolution. But this does not really answer my initial comment. How do we know that the TUS effect is due to the blocking of 45a and not one of the other areas also affected by the TUS (see the cone in figure 6).

Response:

As we discussed in the previous version of our manuscript, the distance between the TUS foci for area 45a and area 47/12o, which was sufficient to produce a double dissociation in both behavioral changes and in resting-state functional connectivity network changes, was 12.5 mm in each hemisphere. These segregated TUS-evoked effects suggest that the spatial range of the TUS effects was narrower than 6.25mm (=12.5mm/2) and therefore TUS targeted to area 45a could not effect neighboring brain areas more than 6.25mm away. Due to the directionality of the ultrasound wave, the area of high intensity ultrasound stimulation is in a spindle shape or a cone, as the reviewer points out. We would like to emphasize that we applied the ultrasound stimulation perpendicularly to the cortical layer. This means that the most sonicated areas in the longitudinal direction were the cortical region that was targeted and then, secondarily, the white matter which is carrying axons to and from that targeted region. How sensitive that white matter is to the TUS is still a matter of research interest but if TUS exerts a major effect on this white matter, then it would mainly be affecting the projections into and out of the targeted region. We have discussed these points in the Results section as follows:

To causally test if functional connectivity networks from the seed at macaque 45a and 47/12o are segregated from each other, we applied bilateral TUS to either area 45a or 47/12o and compared the resting-state

functional connectivity networks[30-34] between pre-TUS and post-TUS scans (4 sessions in total from 2 animals [M2 and M5] for each TUS target). 45a TUS prominently decreased area 45a's functional connectivity with mPFC, dACC and LIP (IPS) compared to the pre-TUS baseline (Fig. S7c, left panel). In contrast, TUS targeted to area 47/12o prominently decreased 47/12o's functional connectivity with area 8A (FEF) and pIT (Fig. S7c, right panel). These dissociations in altered functional connectivity profiles after 45a and 47/12o TUS support the claim that TUS of either 45a or 47/12o modulates separate subnetworks of the brain in macaques. These segregated TUS-evoked changes suggest that the spatial range of the TUS effects was narrower than half the distance between the areas 45a and 47/12o (12.5mm/2=6.25mm). The nature of the ultrasound waves ensured that the area of high intensity ultrasound stimulation was in a spindle shape (Fig. S7a). However, we applied the ultrasound stimulation perpendicularly to the cortical layer and the most sonicated areas in the longitudinal direction were the targeted cortical areas themselves and then, secondarily, the adjacent white matter regions carrying connections to and from the targeted areas.

While parts 1 and 2 are now acceptable given appropriate rewriting, part 3 remains below the standards and should be removed;
Both in the reply to my comments as in the revised text, the authors claim the correlations across studies between fc and some type of anatomical connection indicate the value of this technique; this highly disputable as for given fc one cannot know which is the underlying anatomy: it can be a monosynaptic connection, but equally well a di- or multi-synaptic connection. In the latter cases even if the number of intervening synapses is equal, they can very well be located in different anatomical structures. Hence the comparison of spider plots as used all the time in part 3 is impossible: equal values do NOT mean the same anatomical connection. In addition differences between 'spider' plots cannot be assessed as no variability is provided. Furthermore when comparing spider plots between species more problems arise as it has to be assumed the brain regions used in the spider plots are homologous. This is a difficult enterprise and for very few cortical areas, outside primary areas, homology between macaque and human have been proven, MT and its cluster being a nice exception. The authors are aware of the problem and stated that the areas in the spider plots they compare show similar functional activity in their fMRI data. This is insufficient as multiple converging evidence is needed to demonstrate homology.
Given the size and the complexity of the manuscript, dropping the third weak part would free space to point out the limitations of the data described in the valuable first and second parts.

Response:
As the reviewer suggests, the strength of functional connectivity does not correspond one-on-one with that of anatomical connections. Some strong functional connectivity is sometimes due to a monosynaptic connection, but equally due to di- or multi-synaptic connection. In fact, work from our own laboratory confirms that this is the case; O'Reilly and colleagues (PNAS, 20213) showed that significant functional connectivity remained between two homotopic areas in the two hemispheres when the direct monosynaptic connection between them, running via the corpus callosum, was transected. The functional connectivity between the areas on

decreased to zero when all other polysynaptic pathways between the areas, running via the anterior commissure, were also transected. The reviewer is correct to say that caution is needed in interpreting resting state functional connectivity for this reason but, we note, in this case we are comparing identical measures in the two species. Thus, it is possible to illustrate the comparison as shown in figure 7 and to note that the circuit level similarities between the areas in the two species may reflect a mixture of monosynaptic and polysynaptic connection routes in both species. As the reviewer also suggests, to establish the inter-species homology of brain areas in general, multiple converging evidence is ideal. However, in the present study, we aimed to reveal the functional homologies of brain network to enable prospective metacognition between humans and monkeys. Therefore, our interpretation of the spider plots is not simply based on just the spider plots themselves but, in addition, it is also based on the other findings that we report in the manuscript: the same areas have distinct and specific patterns of activity during the prospective judgement task and, when stimulated with TUS, they are associated with distinct patterns of behavioral change. For the above reasons, the functional connectivity metrics are assumed to provide an incomplete but nevertheless useful approximation to the known anatomy generated with track tracing studies. Thus, we have kept the connectivity section in the manuscript but together with a note of warning that the inter-species relationship between the areas should be interpreted with some caution and that the circuit level similarities between the areas across species may reflect a mixture of monosynaptic and polysynaptic connections as follows:

Caution is needed in interpreting resting state functional connectivity data as they reflect a mixture of monosynaptic and polysynaptic anatomical connections[27]. However, in this study, by comparing identically derived functional connectivity measures in the two species we are able to establish some evidence that the areas in the two species – areas 45a and 47/12o – are each a part of a similar circuit in both species. An additional caveat when making comparisons of the functional connectivity of two areas across species is that the comparison is based on the assumption that the other areas from which functional connectivity measures are taken – for example, the areas on the circumference of the spider plots in figure 7b – are similar across species. In the current study we have tried to focus on areas where there is evidence for such correspondences but it is important to consider other lines of evidence for cross-species homology such as the task-related activity patterns and the TUS / TMS-induced patterns of impairment seen when the areas in the two species are stimulated.

Minor comments:

1) Authors should diversify the references rather than auto-quote; In particular an appropriate reference for the division of area 45 into a and b is needed: Petrides & Pandya (2002) only addressed area 45 in general.

Response:

Thank you for the suggestion. We have expanded citations of macaque functional MRI studies. Especially for the division of area 45 into 45a and 45b, we have also cited Gerbella et al. (Cerab. Cortex 2010) and Wardak et al. (Cerab. Cortex 2010) as follows:

Introduction

We then present evidence from recording and causal intervention studies for the existence, in the macaque brain, of two specialized zones, 47/12o [9] at the border between ventrolateral prefrontal cortex and orbitofrontal cortex and area 45a [10,11] in ventrolateral prefrontal cortex that are, respectively, concerned with evaluation of future contingencies linked to potential states of the environment and future behaviours that might be performed in those states.

Results

Activity covarying with changes in the decision variable guiding the first stage judgement was most prominent in ventrolateral prefrontal area 45a (based on Gerbella et al. [10], MNI coordinates, [x,y,z]=[21.1, 7.5, 12.5], Z = 3.75).

2) In the discussion, migration of afferents to a different cortical area, is NOT the same as fusion of two areas; the authors may have to accept this point explicitly.

Response:

We are not advocating any argument for areal fusion; we do not believe that at any point, that we have raised the possibility that areas that are separate in the macaque become fused in the human brain. In preparing this latest revision of the manuscript, we have, nevertheless, followed the reviewer's suggestion and we have explicitly clarified that migration of afferents to a different cortical area would potentially form a new area rather than fusing two existing areas as follows:

We propose that since the time of their common ancestor, the cortex that corresponds to 47/12o in the macaque is, in humans, likely to have expanded during the course of human evolution. As a result, anatomical projections that may have been confined to area 45a in the macaque may extend beyond area 45a into adjacent areas such as area 47/12o in humans. A part of 47/12o might have been 'colonized'[49] by these connections and developed as human alPFC, while other human areas that are also successors to an ancestral 47/12o region may not have been colonized by projections associated with area 45a in the macaque. We emphasize that we are not proposing, however, that areas 45a and 47/12o, as they exist in the macaque, have become fused in the human brain. In other words, if alPFC is indeed an evolutionarily novel area unique to humans and with a specialized function, macaques cannot perform the task by activating a single homologue of human alPFC if they do not possess it. Instead, we should expect that if macaques are able to perform the task at all, then they may well do so via the assistance of activity patterns in more than one of the less specialized brain areas they do possess, such as areas 45a and 47/12o.

Caution is needed in interpreting resting state functional connectivity data as they reflect a mixture of monosynaptic and polysynaptic anatomical connections. However, in this study, by using the identical measures

in the two species, such direct comparisons between human alPFC and macaque areas 45a and 47/12o were enabled.

Reviewer #2:

Remarks to the Author:

The authors have carefully addressed my concerns. I have no further comments.

Response:

Thank you for highly evaluating our revision.

Reviewer #3:

Remarks to the Author:

The authors have addressed all my remaining concerns.

Response:

Thank you for highly evaluating our revision.
